# Enzyme-mediated alkynylation enables transcriptome-wide identification of pseudouridine modifications

**DOI:** 10.1038/s41467-026-70597-8

**Published:** 2026-03-23

**Authors:** Yuru Wang, Kinga Pajdzik, Yutao Zhao, Chang Ye, Talbot Stone, Li-Sheng Zhang, Wen Zhang, Mahdi Assari, Wenxin Zhao, Caraline Sepich-Poore, Qing Dai, Ke Wang, Minkui Luo, Tao Pan, Chuan He

**Affiliations:** 1 Department of Medicinal Chemistry, University of Utah, Salt Lake City, UT USA; 2 Department of Biochemistry and Molecular Biology, The University of Chicago, Chicago, IL USA; 3 Department of Chemistry, The University of Chicago, Chicago, IL USA; 4 Howard Hughes Medical Institute, The University of Chicago, Chicago, IL USA; 5 Institute for Biophysical Dynamics, The University of Chicago, Chicago, IL USA; 6 Chemical Biology Program, Memorial Sloan Kettering Cancer Center, New York, NY USA; 7 Department of Pharmacology, Weill Cornell Medical College, Cornell University, New York, NY USA; 8 Present Address: Department of Applied Biology and Chemical Technology, The Hong Kong Polytechnic University, Hong Kong, China; 9 Present Address: Department of Chemistry, The Hong Kong University of Science and Technology, Hong Kong, China

**Keywords:** Genetic mapping, RNA, RNA modification, Transferases, Sequencing

## Abstract

Pseudouridine (Ψ) is one of the most abundant chemical modifications and plays important roles in RNA function. Advances in our understanding of Ψ have been hindered by a limit of robust methods to precisely and sensitively map their distributions in cellular RNAs. Here, we present ELAP-seq (Enzymatic Labeling and Pull-down for Sequencing) for Ψ detection, which leverages a naturally occurring N1-methyl pseudouridine methyltransferase from *Methanocaldococcus jannaschii* (Mj1640). This enzyme promiscuously converts Ψ to N1-methyl-Ψ (m1Ψ) or installs a propargyl group at the same location in vitro under a mild condition, exhibiting high sensitivity and specificity, and is also functional inside cells. ELAP-seq enriches Ψ-containing RNA fragments and enables single-nucleotide-resolution Ψ detection with markedly enhanced signal-to-noise ratio and reduced sequencing and computational demands. Using ELAP-seq, we identify thousands of candidate Ψ sites in human HeLa and HEK 293 T transcriptomes, validating many previously identified sites as well as reporting additional ones. This versatile enzymatic platform expands the toolkit for sensitive labeling and detection of Ψ, advancing the study of RNA modification biology.

## Abstract (web summary)

Understanding the biological functions of the RNA modification pseudouridine relies on robust methods to map its distribution. Here, the authors identify a methyltransferase that specifically labels this modification and develop a pulldown approach to enrich modified RNAs, enabling transcriptome-wide profiling of pseudouridine sites.

## Introduction

Chemical modifications of RNA are critical to most RNA functions^1^. Among the over 150 types of RNA chemical modifications, pseudouridine (Ψ) is one of the most abundant, and exists in multiple types of RNA species^2,3^. Ψ is formed via the isomerization of uridine, a reaction catalyzed either by various stand-alone Ψ synthases (PUS) or by the DKC1 complex guided by H/ACA box small nucleolar RNAs^3,4^. The enzymatic cores of the Ψ synthases are conserved, and their mutations have been associated with human diseases, including autoimmune digestive disorders and intellectual disability^5–7^. The presence of Ψ in RNA can alter RNA properties, including structure, stability, alternative splicing, and translation^8–12^, and Ψ modifications can have a profound effect on cellular activities such as cell proliferation and stem cell differentiation^13,14^. Ψ has also been found to exist in viral RNAs and may play roles in viral infection and antiviral defense pathways^15^. Furthermore, Ψ and its derivative N^1^-methylpseudouridine (m^1^Ψ) are among the most critical nucleotide substitutions that notably promote efficacies of mRNA vaccines^16^. The correlation of Ψ with human diseases, along with its therapeutic potentials in developing RNA-based drugs, have inspired enormous interest to investigate their natural presence and biological functions in cells.

While Ψ has been extensively studied in the context of abundant rRNA, tRNAs, and ncRNAs for decades, its significance in less abundant mRNAs remains poorly understood, largely due to a lack of robust methods for sensitive and precise detection of Ψ modifications in mRNA species. Ψ shares the same base pairing property as uridine, making it indistinguishable in Sanger and next-generation sequencing. Existing detection strategies largely rely on chemical treatments that convert Ψ into forms generating stop, deletion, or mutation signatures at or near the modification sites during reverse transcription^17–21^. Notable recent examples include RBS-seq, BID-seq, and PRAISE, which utilize bisulfite treatment, and BACS, which employs N1-specific Michael addition of acrylonitrile, for qualitative or quantitative transcriptome-wide Ψ mapping^17,21–24^. While accurate, these methods require deep sequencing due to a lack of enrichment, which leads to high sequencing cost and demanding computing capacity and may also result in missing sites in lowly expressed genes and/or with lower modification fractions. In addition, methods based on bisulfite treatment rely on nucleotide deletion signatures, making it difficult to determine the exact location of the modification among consecutive uridines. An enrichment-based method using CMC addition has been developed, but prolonged chemical treatment led to severe RNA degradation, therefore requiring a high level of RNA input, and incomplete CMC release from non-specific addition to unmodified nucleobases could lead to a non-negligible rate of noise signals, especially under the enrichment conditions^20^. Meanwhile, direct Ψ detection via nanopore sequencing has emerged, yet it remains limited by relatively low resolution, high RNA input requirement, and complex data analysis pipelines^25,26^. Current transcriptome-wide studies have reported hundreds to a few thousands of modification sites. However, LC-MS/MS experiments suggest that Ψ abundance in mRNA is comparable to *N*^*6*^-methyladenosine (m^6^A, 0.2–0.4% in HeLa and HEK 293 T cell lines)^20^, implying that potentially more Ψ sites are to be discovered. Thus, new methods using independent strategies are highly desired to cross-validate previously reported modification sites as well as identify additional modification sites. Furthermore, existing labeling strategies are restricted to isolated RNAs, meaning that there is no feasible way to label Ψ inside cells so far.

To provide additional approaches for both in vitro and potential cell-based manipulation of Ψ, in this study, we examine several natural Ψ-specific methyltransferases for in vitro reactivity and focus on a N1-methyltransferase from *Methanocaldococcus jannaschii* that naturally targets Ψ in tRNA^27^. We find that this enzyme can install a methyl or propargyl group onto the N1 position of Ψ in various sequence contexts independent of RNA structures. The labeling strategy also exhibits higher specificity and sensitivity compared to currently available antibodies. This discovery provides a simple and mild one-step reaction to specifically yet broadly label Ψ under physiological conditions, and can potentially be used to modulate Ψ inside human cells, making it a highly promising and versatile strategy to detect and modulate Ψ. To showcase the utility of this labeling strategy, we leverage it to develop a method that enriches Ψ-containing transcripts for transcriptome-wide Ψ profiling. We validated the method by applying it to detect known Ψ modifications in rRNA and then used it to identify candidate Ψ modification sites in the transcriptomes of HeLa and HEK 293 T cell lines. Thousands of candidate modification sites are identified for each cell line, which validate many previously reported sites while also revealing additional ones, providing insights into features of Ψ distribution and function in human cells. Altogether, our study provides a tool that allows studies of Ψ modification both in vitro and potentially in cells.

## Results

### A Ψ-specific methyltransferase that labels Ψ modifications

Currently, labeling tools to investigate RNA Ψ modification are limited. To address this gap, we explored natural enzymes that specifically act on Ψ. Natural enzymes often serve as valuable starting points to develop biotechnologies thanks to their exceptional substrate specificity, catalytic efficiency, and ability to function inside cells^28^. A plethora of proteins and enzymes exist that selectively recognize and act on RNA modifications; exploiting these biomolecules offers a promising direction for the modulation of RNA modifications^28^.

**Fig. 1:**
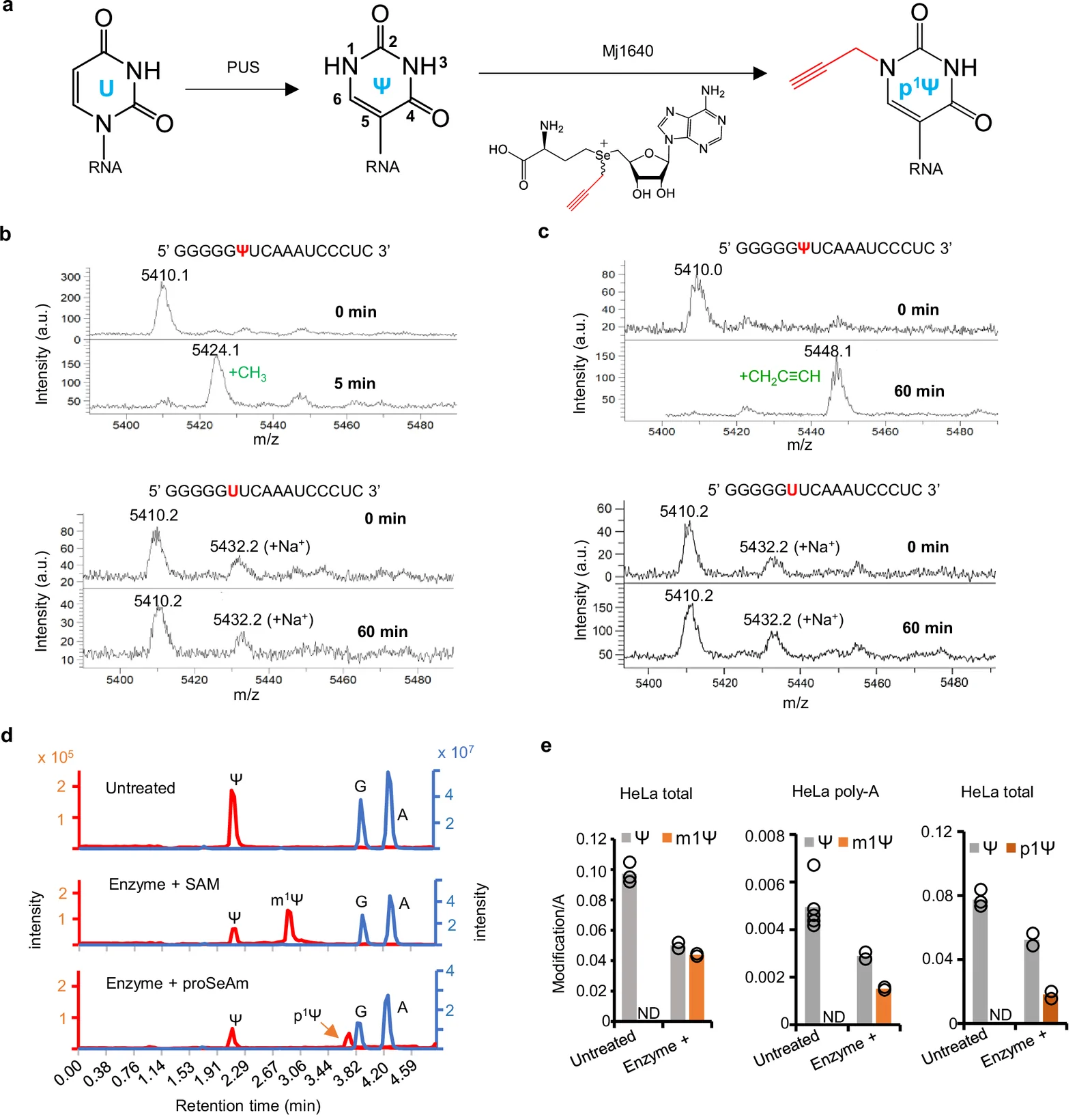
Methyltransferase Mj1640 can label Ψ specifically and promiscuously in vitro. **a** Enzymatic conversions of uridine to Ψ and Ψ to *N*^*1*^-propargylΨ (p^1^Ψ). **b** MALDI-TOF MS showing that Mj1640 specifically methylates Ψ (top) but not U (bottom) in 17-mer synthetic RNA oligonucleotides. **c** MALDI-TOF MS shows that Mj1640 can transfer a propargyl group onto Ψ in the synthetic RNA oligonucleotide by making use of Propargyl-SeAm co-factor. **d** LC-MS/MS signals of nucleosides Ψ, *N*^*1*^-methylΨ (m^1^Ψ), and p^1^Ψ in digested total RNA with and without enzymatic treatment. **e** Quantification of efficiencies of the enzymatic addition of the methyl or the propargyl group to Ψ in cellular total or poly-A RNA. For HeLa total RNA methylation, *n* = 3 and *n* = 2 for the untreated and enzyme+ conditions, respectively. For HeLa poly-A RNA methylation, *n* = 5 and *n* = 2 for the untreated and enzyme+ conditions, respectively. For the HeLa total propargylation, *n* = 3 and *n* = 2 for the untreated and enzyme+ conditions, respectively. Source data of (**d** and **e**) are provided as a Source Data file.

One major physicochemical difference between uridine and Ψ is an extra hydrogen donor at the N1 position of the Ψ nucleobase (Fig. 1a). We reasoned natural enzymes specifically reacting at this position would facilitate selective labeling of Ψ over uridine. *N*^1^-methylpseudouridine (m^1^Ψ) is a modification naturally found in tRNA of most archaea and is formed via methylation at the N1 position of Ψ, a reaction catalyzed by SPOUT-class methyltransferases^29–32^. We screened a few SPOUT-class methyltransferases that catalyze this conversion and focused our attention on an enzyme named Mj1640 from *M. jannaschii*, which is responsible for methylation of Ψ at position 54 in the T-arm of tRNAs (Supplementary Fig. 1a)^27^. Although the methylation was reported to occur naturally only on Ψ54 in tRNA, Wurm et al. reported that the enzymatic catalysis is highly efficient in vitro on a 17-mer RNA probe resembling the T-arm of *M. jannaschii* tRNA^Trp^ at 65 °C^27^. We reasoned that the relatively high reaction temperature may have disrupted the secondary structure of the 17-mer RNA probe, thus the enzyme might be more promiscuous for labeling Ψ independent of local RNA structure.

We proceeded and tested activity of recombinant Mj1640 protein (Supplementary Fig. 1b–e) in vitro on the same 17-mer synthetic RNA oligonucleotide as by Wurm et al.^27^. Encouragingly, the Ψ-RNA was methylated to > 90% in five minutes as evaluated by MALDI-TOF MS, whereas no reaction was observed for a control RNA with a U replacing the Ψ after over a one-hour incubation (Fig. 1b), validating the excellent specificity of the reaction towards Ψ. Importantly, a synthetic 8-mer oligo containing a Ψ site was also efficiently methylated (Supplementary Fig. 1d), whereas the control oligo with U was not, demonstrating that the enzymatic catalysis does not rely on RNA secondary structure. Sequence preference of this enzyme was also studied and will be shown later.

In addition, we found that when expressed in human HEK 293 T cells, the Mj1640 enzyme is functional to convert Ψ in endogenous transcripts to m^1^Ψ (Supplementary Fig. 3b–d). Altogether, our study presents an enzymatic strategy for labeling Ψ both in isolated RNAs and inside cells.

### Mj1640 can transfer a propargyl group to Ψ in RNA

Next, we tested whether the Mj1640 enzyme could use the SAM derivatives allylic-SAM or propargylic *Se*-adenosyl-*L*-selenomethionine (propargyl-SeAm) to label Ψ sites with chemical groups suitable for further manipulation. To our delight, Mj1640 efficiently utilized both as cofactors to label the 17-mer Ψ-RNA ( > 80% in 1 h) and the 8-mer Ψ-RNA, but not the control U-RNAs (Fig. 1c and Supplementary Fig. 1d, e).

To evaluate the ability of the enzymatic reaction to label Ψ modifications in biological RNA, we assessed the labeling efficacy on RNA extracted from HeLa and HEK293T cell lines using quantitative LC-MS/MS. We detected a dramatic decrease of Ψ signal and the emergence of *N*^*1*^-methylpseudourisine (m^1^Ψ) or *N*^*1*^-porpargyl-pseudouridine (p^1^Ψ) signal after the enzymatic treatment with either SAM or propargyl-SeAm as the co-factor (Fig. 1d and Supplementary Fig. 2a). With optimized protein purification and enzymatic reaction conditions (see the “Methods” section), the Ψ in vitro methylation efficiency was around 50% for total RNA, and 33–48% and 50–60% for poly-A RNA of HeLa and HEK293T cells, respectively (Fig. 1e and Supplementary Fig. 2a–c). The overall 33–60% m^1^Ψ/Ψ ratio indicates that a considerable proportion of Ψ modifications were successfully labeled. Additionally, total RNA and poly-A RNA were labeled with the propargyl group at ~25–30% of all Ψ (Fig. 1e and Supplementary Fig. 2d–g). This slightly lower labeling percentage compared to methylation is likely due to a weaker binding affinity of the enzyme to propargyl-SeAm than to SAM rather than a biased labeling. These results demonstrated the feasibility of using Mj1640 to label Ψ in biological RNA. It is important to note that the enzymatic reaction condition is mild, resulting in largely intact mRNAs with ~80% recovery rate (Supplementary Fig. 3a). This observation highlights one advantage of this enzymatic labeling strategy over chemical labeling approaches that typically cause RNA degradation.

### Mj1640 labeling assay exhibits superior sensitivity and specificity compared to antibodies

Pseudouridine has clear relationship with several diseases; however, the potential of both PUS activity and global modification signatures as biomarkers for disease diagnosis has yet to be effectively evaluated. The click chemistry enabled by our enzymatic assay allows flexible attachment of different functional groups for signal readout, suggesting its potential suitability for diagnostic or detection applications.

To evaluate this potential, we characterized the specificity, sensitivity, and quantitative performance of the enzymatic labeling strategy by clicking either a fluorophore or biotin onto the modification for fluorescence- or chemiluminescence-based dot blot analyses. We observed a strong linear correlation between the amount of modified RNA and the detected signal (Supplementary Fig. 4a–c), and high specificity was achieved in both synthetic and total RNA samples (Supplementary Fig. 4a–c). Importantly, the chemiluminescence-based dot blot assay can detect as little as 0.05 ng of synthetic RNA oligos (one modification in 33-mer RNA oligonucleotides) (Supplementary Fig. 4c), demonstrating substantially higher specificity and sensitivity than currently available antibodies (Supplementary Fig. 4d, e). Together, these preliminary results indicate that this enzymatic labeling strategy has strong potential to serve as the basis for diagnostic and detection tool development.

### Development of the enzymatic labeling and pull-down for sequencing (ELAP-seq) method for detecting Ψ in the transcriptome

**Fig. 2:**
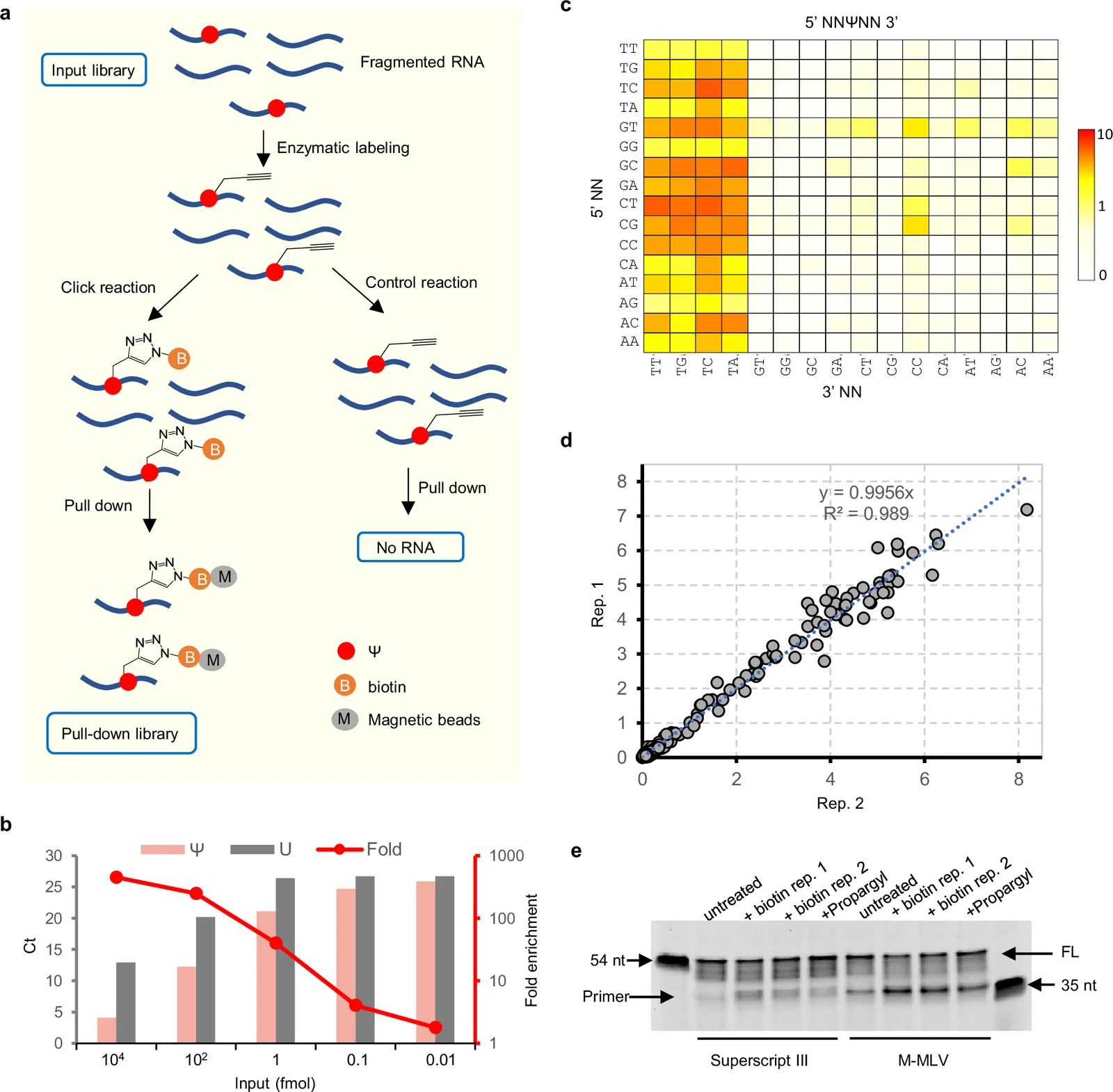
The development of the ELAP-seq method. **a** ELAP-seq library preparation workflow. **b** Pull-down and qPCR of RNA oligonucleotides bearing Ψ or U after enzymatic treatment and biotin addition reveal high signal-to-noise ratios. **c** Heatmap shows various degrees of enrichment for synthetic RNA oligonucleotides covering 256 types of sequence motifs surrounding the Ψ modification (5′-NNΨNN) as evaluated by ELAP-seq. **d** Correlation between two independent replicates on degrees of enrichment for synthetic RNA oligonucleotides with 5′-NNΨNN motifs. Each dot represents one sequence motif. **e** A reverse transcription assay to evaluate RT reaction conditions. With low dNTP concentration, Superscript III and M-MLV RT were arrested by *N*^*1*^-propargylΨ modification with (“+ biotin”) or without (“+ Propargyl”) clicking on a biotin molecule. The bands in the first lane and the last lane serve as molecular weight markers. This experiment was repeated independently once with similar results. Source data of (**b**–**e**) are provided as a Source Data file.

To apply the enzymatic labeling strategy to study Ψ modification in the transcriptome, we developed a method coupling Enzymatic Labeling and Pull-down for Sequencing (ELAP-seq) (Fig. 2a). Compared to methods using direct sequencing, ELAP-seq enriches the modified RNA for sequencing, which provides the advantages of effectively increasing signal-to-noise ratios, reducing sequencing and computational cost, and allowing for more comprehensive mapping of Ψ even in lowly expressed mRNA species or those with low stoichiometry. The enzyme’s exclusive specificity towards Ψ is critical to ensure the specific enrichment of Ψ-modified RNA, thereby reliable identification of the modification.

In this protocol, biological RNA such as total RNA or mRNA is first fragmented to 60–200 nucleotides, followed by enzymatic treatment to label the Ψ with a propargyl group (Fig. 2a). An aliquot of fragmented RNA set aside prior to enzymatic treatment is used to directly prepare a sequencing library to control for signals from factors other than Ψ existing in the starting sample (input library) (Fig. 2a). The enzymatically labeled RNA is then split into two parts, with one part undergoing a copper-catalyzed azide–alkyne cycloaddition (CuAAC or click) reaction to add on a biotin molecule while the other part undergoing a mock click reaction (control library). Pull-down is then performed with streptavidin-coated magnetic beads to enrich RNA fragments containing Ψ modifications, followed by library construction and Illumina sequencing (pull-down library) (Fig. 2a). The control library is expected to yield no detectable DNA product under the same conditions used for pull-down library construction and is not subjected to sequencing.

To evaluate the signal-to-noise ratio of the ELAP strategy, we treated synthetic RNA oligonucleotides bearing either a Ψ or U in the center with enzymatic labeling, biotin addition, and pull-down sequentially, and then quantified the amount of the resulting RNA material via RT-qPCR. With an optimized pull-down buffer condition, the Ψ-RNA was enriched for ~ 450-fold relative to the U-RNA with input ranging from 0.01 fmol to 10 pmol (Fig. 2b), demonstrating an excellent signal-to-noise ratio.

### ELAP-seq reveals sequence preference of Mj1640 and detects Ψ at single base resolution

Before applying ELAP-seq to biological samples, we first used it to comprehensively evaluate the sequence context preference of the Mj1640 enzyme by building sequencing libraries from a mixture of 33-mer synthetic standard RNA oligonucleotides containing NNΨNN sequences (*N* signifies any nucleoside), which cover 256 sequence contexts surrounding Ψ. As expected, the control library yielded no sufficient material to produce a sequencing library in the end unless being over-amplified, highlighting the low background. Interestingly, sequencing data from the pull-down libraries showed that RNAs with 5′-ΨU-3′ context were preferentially enriched while marginal sequence preferences were observed at other nearby sites (Fig. 2c). In contrast, control RNA oligonucleotides containing NNUNN sequences, whose sequencing libraries were built with overamplification, showed no such specific enrichment of any RNA (Supplementary Fig. 5), further demonstrating the high specificity of this method.

To further validate the observed sequence preference, we synthesized 10-mer RNA oligonucleotides containing degenerate A, U, C, and G (denoted as N) at one of the four positions within two nucleotides upstream or downstream of the target Ψ site, and evaluated the enzymatic reaction efficiency on these oligonucleotides using MALDI-TOF MS. In agreement with the ELAP-seq results, Mj1640 does not show obvious selectivity for nucleobases at the −2, −1, and +2 positions but exhibits preference for uridine over other nucleobases at the +1 position (Supplementary Fig. 6). Importantly, the strong concordance between the ELAP-seq result and the MALDI data underscores the reliability of the ELAP-seq approach.

These results are consistent with a previous report that Mj1640 requires 5′-Ψ (U/ Ψ)−3′ context for optimal catalysis^29^. Earlier research indicates that ~30% of Ψ sites in mRNA are found in the 5′-ΨU-3′ context^19,20^, the preferred sequence context of Mj1640, as supported by our observation that the Mj1640 converted Ψ in cellular mRNA to m^1^Ψ at a level of 30–60% (Fig. 1e and Supplementary Fig. 2). Despite this sequence preference, our enrichment protocol allows pulling down oligonucleotides of almost all sequence contexts to various extents (Fig. 2c). Meanwhile, two independent pull-down experiments of the standard oligos resulted in highly consistent results (Linear correlation with R^2^ = 0.98) (Fig. 2d), demonstrating excellent reproducibility of the pull-down protocol.

Pull-down of fragmented RNA for sequencing typically yields Ψ signals at a resolution of 60–200 nt. To achieve detection of Ψ at single base resolution, we found that a 500× lower dNTP concentration than recommended in the commercial manual led to significant arrest of superscript III or M-MLV at the alkynylated Ψ site with or without the addition of a biotin molecule (Fig. 2e). Collectively, the enrichment of RNA fragments containing Ψ in various sequence contexts, coupled with the reverse transcription (RT) stop signature at the labeled sites, would enable ELAP-seq to specifically identify Ψ modification sites at single base resolution.

### Validating ELAP-seq by detecting known Ψ modifications in rRNA

**Fig. 3:**
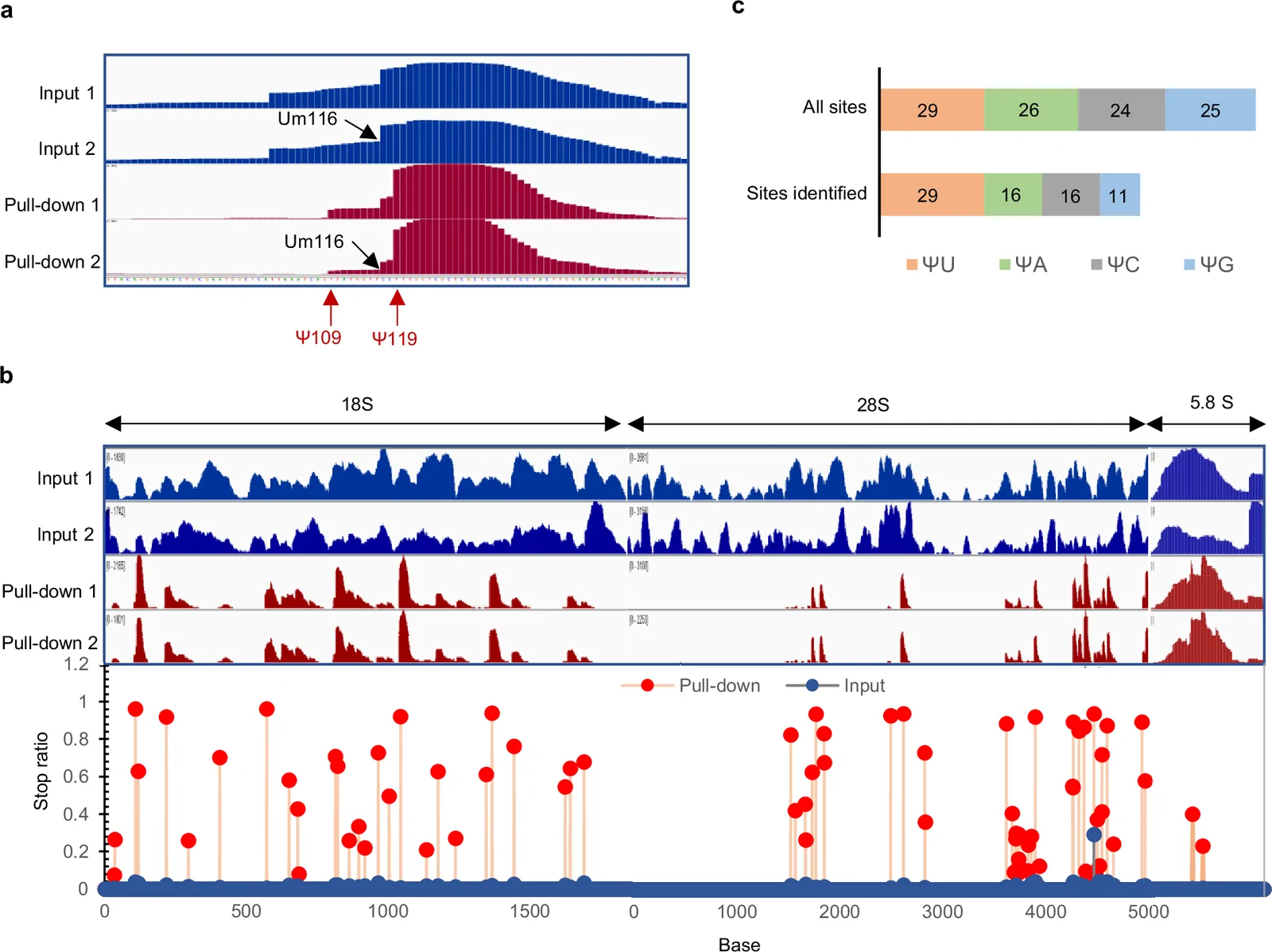
ELAP-seq detects known Ψ modification sites in rRNA. **a** IGV snapshot shows distinct RT stop patterns between Ψ and N_m_. Stop signatures at Ψ are generated only after enzymatic treatment, whereas N_m_ signatures exist in both the input and the pull-down samples. **b** IGV snapshot showing the input and pull-down maps on rRNA genome (top) and RT stop sites representing Ψ modifications in rRNA (bottom). Data of 5.8S uses combined reads from superscript III and IV libraries. **c** A summarization of identified rRNA sites in different sequence contexts. Source data of (**b**) are provided as a Source Data file.

Next, we tested the efficacy of ELAP-seq in the context of biological samples. We first evaluated its ability to detect known Ψ modifications in ribosomal RNA^33^. We used superscript III with the low dNTP concentration that facilitates the generation of stop signatures as shown in Fig. 2e. As expected, reverse transcription mediated by superscript III was substantially arrested after putting an adenosine opposite the Ψ, generating a drastic stop signature right at the site of the modification (Fig. 3a). Importantly, Ψ modifications were specifically identified based on the stop signatures uniquely generated after enzymatic treatment (Fig. 3a and Supplementary Fig. 7b), whereas Nm sites in rRNA were effectively filtered away based on the same stop signature in the input library (Fig. 3a and Supplementary Fig. 7b, c).

We followed the filtering criteria described in the “Methods” section to call modification sites. Notably, we required that a site be identified in two independent biological replicates in order to be included in our final list of modification sites. Our method successfully identified 72 out of 104 known Ψ sites in rRNA (69.2% sensitivity) at single base resolution, covering 29/29 sites in the 5′ ΨU 3′ context and 41/75 sites in other contexts (Fig. 3b, c). Our method also led to stop signatures at U1615 of 18S, whose originalities are unclear, as well as U31 and U630 of 18S and U1603 of 28S, which likely originated from off-target mapping of short intronic reads to rRNA genes (95% accuracy) (Supplementary Data 5). The known Ψ sites undetected by our method were primarily due to the clustering of these sites in rRNA (Supplementary Fig. 7d), leading to low stop signature below the detection limit of our method.

### ELAP-seq identifies thousands of candidate Ψ sites from the human transcriptome

**Fig. 4:**
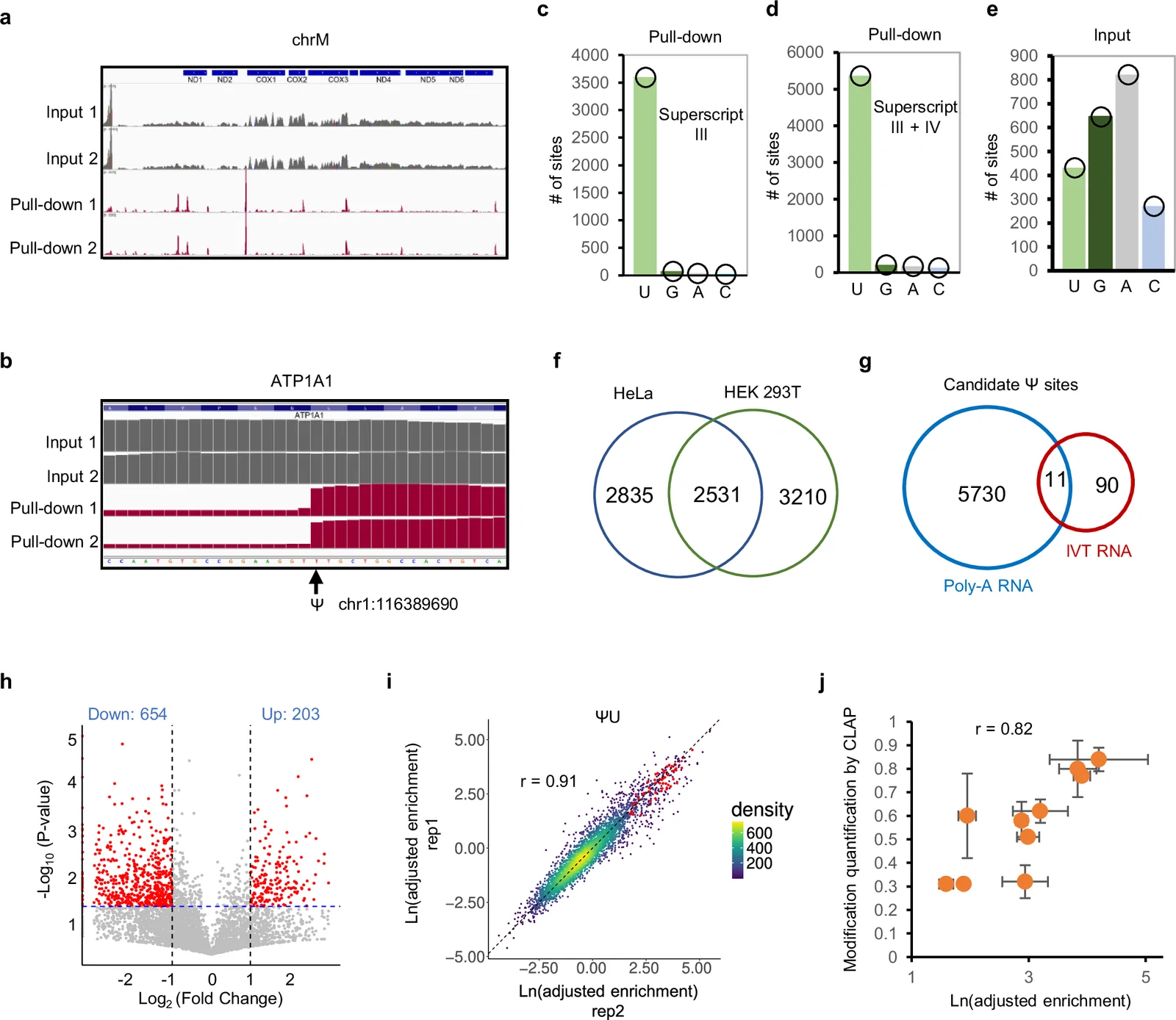
ELAP-seq identified thousands of candidate Ψ sites from HeLa and HEK 293 T cell lines. **a** IGV snapshot showing reads mapping to the mitochondrial chromosome as an example of the pull-down profile. **b** An example of a Ψ modification site determined based on prominent stop signatures in the pull-down samples. **c**, **d** Base distribution of RT stop sites identified from HeLa poly-A RNAs using libraries generated with Superscript III alone (**c**) or combined libraries generated with Superscript III and Superscript IV (**d**). **e** Base distribution of RT stop sites identified in the input samples of HeLa poly-A RNAs. In (**c–e**), circle represents the data point, *n* = 1. **f** Overlap of candidate Ψ sites identified from HeLa and HEK 293 T cell lines. **g** Overlap of candidate Ψ sites between the poly-A RNA samples and the in vitro transcribed (IVT) RNA samples. **h** Volcano plot of sites with differential enrichment levels between siCtrl and siDKC1. Fold Change is the ratio of the average enrichment level for a site in siDKC1 samples divided by that in siCtrl samples. Red dots represent sites with over twofold change in enrichment values and *p*-values < 0.05 in one-sided Student’s *t*-test. One-sided statistical testing was used because the knockdown was expected to reduce modifications. **i** Logarithmic form of the adjusted enrichment level, which indicates relative modification extent for sites in the ΨU contexts. Red dots denote known modification sites on rRNA. **j** Correlation of relative modification levels estimated by ELAP-seq to the absolute modification levels quantified by CMC-RT and ligation-assisted PCR analysis of Ψ modification (CLAP) for 10 mRNA Ψ sites. Data are presented as mean values +/− SD, *n* = 3. Source data of (**c**–**e** and **h**–**j**) are provided as a Source Data file.

We next applied ELAP-seq to poly-A RNAs purified from HeLa and HEK 293 T cell lines (Supplementary Fig. 9a). Supporting the specific enrichment of modified RNAs, we found selective enrichment of peaks in the pull-down samples, as exemplified by the maps on the mitochondrial chromosome (Fig. 4a). In addition, candidate Ψ modification sites are clearly indicated by prominent stop signatures uniquely existing in the pull-down samples and absent in the input samples (Fig. 4b).

We applied the same stop ratio and read coverage parameters that were successfully used for detecting rRNA modification sites to identify candidate modification sites in mRNA (See Supplementary Fig. 8 and the “Methods” section). Similarly, only sites that are identified in two independent biological replicates are included in the final list. With our pipeline, we identified 3738 RT stop sites from HeLa poly-A RNA libraries constructed with superscript III, among which 3600 sites were located at U (96.3%), 138 at non-U (3.6%) (Fig. 4c). Similarly, in HEK 293 T cells, 3473 RT stop sites were identified, with 3331 at U (95.9%), 142 at non-U (Supplementary Fig. 9b). The U sites showing stop signatures were further defined as candidate modification sites. Here, we evaluated stops across non-U nucleotides to provide a measure of specificity and potential false positives. This is based on the rationale that, if no specific labeling and enrichment of pseudouridine occurs, RT stop would occur randomly with similar frequencies at A, U, C, and G. Thus, the small fraction of non-U stops provides an empirical estimate of background RT termination events and the predominant enrichment of the stop signatures specifically at U compared to any other nucleotide (64-fold), which was not seen in the control input samples (Fig. 4e), strongly supports the specific detection of pseudouridine^34^.

While Superscript III with low dNTP produces prominent RT stop signature, we noticed that the coverage at some sites in mRNA is below the reads coverage cutoff, although both replicates share strong stop pattern. To achieve higher read coverage, we also used superscript IV with the commercially recommended concentration of dNTP (0.5 mM final) to generate libraries in parallel from the same pull-down product. Interestingly, superscript IV also generated stop signatures at Ψ, although to a lesser extent (Supplementary Fig. 7a), suggesting that the on-bead RT reaction used in our library construction protocol further facilitated the generation of the stop signature (Supplementary Fig. 7a). To rescue additional sites by increasing the reads coverage, we combined data from superscript IV and III for further analysis. We first tested this analysis strategy on rRNA and verified that, with slightly more stringent cutoff in stop ratio (See “Methods” section), this strategy did not introduce additional false positives. Additionally, this analysis enabled the identification of the two modification sites in 5.8S rRNA, which were previously missed due to low coverage (Supplementary Fig. 7e).

Applying the same analysis on mRNA identified an additional 1628 and 2410 candidate sites from HeLa and HEK293T poly(A) RNA, respectively, yielding a total of 5366 candidate sites for HeLa and 5741 for HEK (Fig. 4d and Supplementary Fig. 9b). Of these, 2, 531 candidate Ψ sites are shared between HEK293T cells and HeLa cells, accounting for ~46% of all candidate sites in each cell line (Fig. 4f). We note that some sites passing the filter in one cell line but found in only one replicate in the other cell line were not included. Therefore, the actual number of shared sites between the two cell lines may be higher than reported here.

The Pearson correlations for normalized read coverage RPM of identified Ψ sites between two pull-down replicates is higher than 0.98 (Supplementary Fig. 10a). We performed a third biological replicate, and the three replicates repeatedly identified 3820 modification sites in HEK293T (and a similar number identified for HeLa cell line) (Supplementary Fig. 10b, c). Notably, RPM values for 81% of the 3820 sites shared by all three replicates exhibit excellent coefficient variation values of between 0 and 0.25 across the three pull-down experiments (Supplementary Fig. 10e), suggesting high consistency in pull-down efficiency across biological replicates. The sites identified by only one replicate exhibited significantly lower numbers of stopped reads, with most of them falling below the detection threshold in the other replicates (Supplementary Fig. 10d). This indicates that the discrepancies mainly arise from sites with near-threshold stopped reads counts. In this study, we define candidate modification sites as those detected in at least two independent replicates, which effectively enriches for sites with robust signals.

### Validation of modification sites

To validate the authenticity of the candidate Ψ sites, we used a modification-free HEK mRNA transcriptome prepared via in vitro transcription (IVT) of cDNA of poly-A RNA as a negative control. We first confirmed that the IVT libraries covered gene bodies similar to the native biological poly-A RNA (Supplementary Fig. 11a, b). With enzymatic labeling and pull-down, ELAP-seq could only identify 412 sites from the IVT RNA libraries, with 101 sites located at U (Supplementary Fig. 11c). Of the 101 candidate Ψ sites detected from the IVT RNA libraries, 11 overlapped with the 5741 candidate Ψ sites detected in native biological poly-A RNA samples (Fig. 4g) and were excluded from the final list. This demonstrates that the signals we identified in native RNAs are not artifacts from the labeling or sequencing processes but true modification sites.

We also performed siRNA knockdown for one of the pseudouridine synthases, DKC1, whose expression perturbation has been found to affect a large fraction of modification sites on mRNA^21^. We confirmed the successful RNA and protein knockdown by RNA-seq and western blot, respectively (Supplementary Fig. 12a). Enrichment levels, which were used to compare differential modifications between conditions (see the “semi-quantification capacity of ELAP-seq” section below), showed great correlations between the two biological replicates under both conditions (Supplementary Fig. 12b, c). We identified 654 sites downregulated upon DKC1 depletion (*p*-value < 0.05 and Log_2_(Fold Change) < −1) (Fig. 4h and Supplementary Data 6), including 524 sites newly reported in this study. The changes in expression of genes harboring these downregulated sites are mild and unlikely to substantially affect the efficiency of pull-down of this modification or the reliability of the detected Ψ downregulation events (Supplementary Fig. 12d–g). Notably, the DKC1-dependent sites determined by PRAISE are also predominantly downregulated in our dataset, supporting the reliability of the results (Supplementary Fig. 12h). More studies are required to identify modification sites responsive to knockdown of other pseudouridine synthases.

Lastly, we set out to validate individual candidate Ψ sites using a low-throughput CMC-based RT-qPCR method (Supplementary Fig. 13a). Among the 20 sites producing reliable qPCR signals, 17 were successfully validated, including 9 sites not previously reported (Supplementary Fig. 13b). The ones not successfully validated likely have low levels of modification or labeling that are below the detection limit of the CMC RT-qPCR method without enrichment.

To better evaluate the reliability of individual candidate Ψ sites, we aimed to categorize them into different confidence levels. The non-U sites, which represent noises, exhibited markedly lower stop ratios compared to the U sites (Supplementary Fig. 14a). To define higher-confidence sites, we set a higher threshold for stop ratio (0.3, the median stop ratio of non-U sites) (Supplementary Fig. 14b). Additionally, sites detected in a greater number of replicates exhibited a reduced rate of false positives (Supplementary Fig. 14c). We thereby classified candidate Ψ sites into the following categories: (1) highest-confidence (stop ratio > 0.3 in every replicate in which it is detected and detected in all three replicates, *n* = 3428 sites); (2) higher-confidence (stop ratio > 0.3 in every replicate it is detected, or detected in all three replicates, *n* = 1704 sites); and (3) lower-confidence (stop ratio <0.3 in at least one replicated it is detected, and detected in only two replicates, *n* = 618 sites) (Supplementary Fig. 14d and Supplementary Data 1 and 2).

### Semi-quantification capacity of ELAP-seq

To evaluate the capacity of the method to probe differential modification at a site across different conditions, we calculated the enrichment level at each site by normalizing the local read coverage per million mapped reads (RPM) at the site in the pull-down sample to that in the input sample to account for gene expression difference. We found that these parameters exhibited excellent Pearson correlations greater than 0.95 between two biological replicates for both HeLa and HEK293T cell lines, demonstrating their reliability for comparing differential modification across conditions (Supplementary Fig. 15a).

We further investigated intra-group modification level variations across different sites. We reasoned that the enrichment level is influenced by not only the modification level but also the enzyme’s preference for the sequence context a site is in. To account for this, we adjusted the enrichment level by the sequence preference, which was determined by the corresponding synthetic oligonucleotide (Fig. 2c). Due to the large discrepancy in enzymatic preference between the 5′ ΨU 3′ context and other sequence contexts, we categorized the sites into ΨU and non-ΨU groups for more accurate intra-group comparisons. Additionally, pull-down and qPCR on a synthetic oligonucleotide modified to various levels revealed an exponential correlation between the modification level and the enrichment level (Supplementary Fig. 15b). We thus used the logarithmic form of the adjusted enrichment level as an estimate of the relative degree of modification across different sites.

Supporting the semi-quantification capability of the method, Ψ sites in rRNAs, which are known to be modified to nearly 100%^33^, are distributed at the higher end of the relative modification level spectrum in both groups (Fig. 4i and Supplementary Fig. 15c). Furthermore, we compared the relative modification levels estimated by our method to the absolute modification levels of 10 mRNA Ψ sites quantified by CLAP so far, either previously^17,35^ or in the current study (Supplementary Fig. 15d). Encouragingly, we found a correlation coefficient of 0.82 (Fig. 4j). Reinforcing the semi-quantitative nature of the method, there exists a moderate-to-strong correlation between our quantification and that obtained via PRAISE (correlation coefficient of 0.45–0.79) (Supplementary Fig. 15e). These results suggest that our method is reasonably accurate in distinguishing relative modification levels between sites.

Based on the correlation between our method and the quantification by CLAP (for Ψ in non-ΨU context, *r* = 0.82 (Fig. 4j)) or PRAISE (for Ψ in ΨU context, *r* = 0.79 (Supplementary Fig. 15e)), we further categorized the modification sites into highly modified, moderately modified, and lowly modified groups (Supplementary Fig. 15f and Supplementary Data 1 and 2). This shows that 72% of the modification sites are defined as lowly modified (estimated modification ratio <30%), which is generally consistent with previous reports. Note that quantification may not be valid for clustered sites, as RT arrests could skew the actual enrichment level, leading to weaker correlation between enrichment and modification levels.

### Comparisons to other methods

Our dataset overlaps with 1168 of 3818 sites (30%) collectively identified by PRAISE, BID-seq, and CeU-seq (Supplementary Fig. 16a) for HEK 293 T cells. Specifically, our method successfully identified 46% (961) of the sites reported by PRAISE and 35% (192) of the sites reported by BID-seq (Supplementary Fig. 16a), providing cross-validation for both strategies, while also determining the exact modification locations in multiple Us, a task that is challenging for PRAISE and BID-seq due to the RT deletion signature. Importantly, sites well overlapping with other methods were mainly found in the highest- and higher-confidence categories, supporting the validity of the confidence classification (Supplementary Fig. 16b). We suggest caution when approaching sites in the lower confidence category (constituting 10% of all sites) and recommend additional validation or referring to Integrated Genome Viewer (IGV) to visually assess their reliability.

To address the discrepancy between our method and the other methods, we identified an additional 40.1% PRAISE-only sites (sites not overlapping with ELAP-seq) in our dataset that exhibit prominent RT stop signatures but either appear in only one replicate or have read coverage below our filter cutoff (Supplementary Fig. 16c, d). Only 13.9% of PRAISE-only sites are entirely undetected by our method, and these sites exhibited lower deletion ratios and read coverage in PRAISE compared to the overlapping sites (Supplementary Fig. 16c, e), suggesting that they are intrinsically more difficult to detect. In addition, our method detected 59% (191) of the TruB1-dependent modification sites reported by PRAISE, which are primarily located in the 5′ ΨC sequence context, demonstrating the capacity of ELAP-seq to detect modifications in diverse sequence contexts (Supplementary Fig. 16f). The remaining TruB1-dependent sites undetected by our method exhibited lower deletion or stop ratios and read coverage in both ours and PRAISE dataset (Supplementary Fig. 16g).

ELAP-seq-only sites (sites only detected by ELAP-seq) tend to have lower modification levels and read coverage than sites overlapping with other methods (Supplementary Fig. 16h), suggesting that they could have been missed by other methods due to weak signals. We examined the raw data for 4060 ELAP-seq-only higher- or highest-confidence sites in the BID-seq dataset. Of these, 2567 sites (59%) exhibited deletion ratios above 0.05 in at least one replicate of BID-seq (Supplementary Fig. 16i, j), demonstrating that they are modification signals in BID-seq as well, despite falling below the filtration cutoff. There are an additional 842 sites showing deletion ratios above 0 but below 0.05, with 89% of them having average deletion ratios of between 0.02 and 0.05 across three replicates (Supplementary Fig. 16i, j). Meanwhile, 651 sites had a deletion ratio of 0 (Supplementary Fig. 16i), but they exhibited significantly lower read coverage than the overlapping sites in the BID-seq dataset (Supplementary Fig. 16k), suggesting that they were likely missed due to low gene expression.

Our method identified 17 candidate Ψ sites in HeLa mitochondria mRNA and 24 in HEK mitochondrial mRNA. This set of sites encompassed all five mitochondria mRNA sites for HEK 293 T (chrM: 6293, 7329, 9905, 11154, and 14868) and three for HeLa (chrM: 6293, 7329, and 11154) collectively identified by BID-seq and PRAISE. Notably, our data shows that 15 mRNA modification sites were shared between the two cell lines, including sites 9905 and 14868, which were previously identified only for HEK 293 T cell line (Supplementary Fig. 17). It’s worth noting that most of the mitochondria mRNA sites uniquely detected by ELAP-seq are characterized as lowly expressed or lowly modified (Supplementary Data 1–4), suggesting potential reasons why they were missed by the other methods.

Altogether, our findings indicate that most discrepancies stem from sites that are intrinsically more difficult to detect due to their low modification or expression levels. This highlights the value of our method for sensitive detection and validation of weak signals overlooked by other methods.

### Insights into Ψ biosynthesis and function

**Fig. 5:**
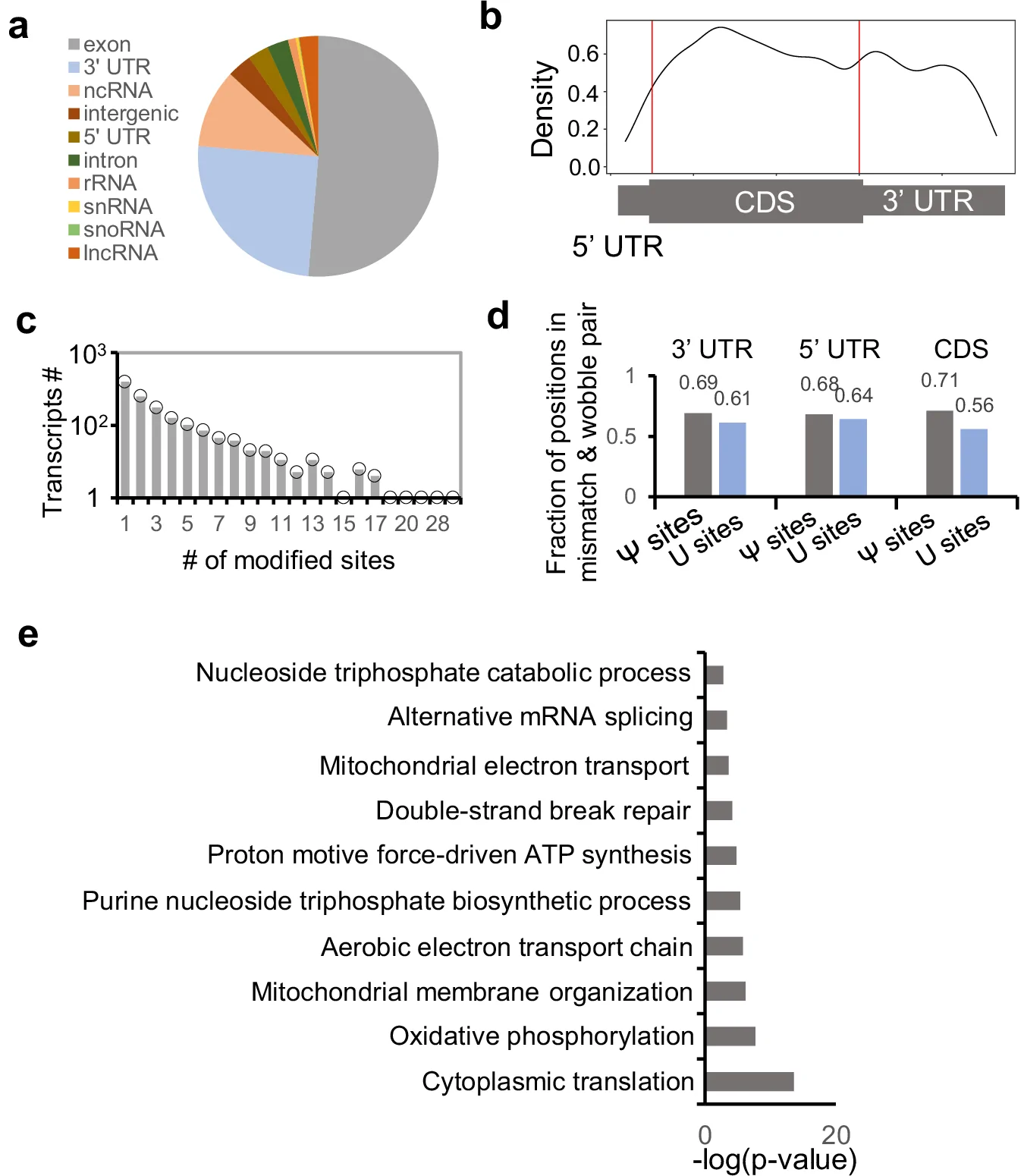
Insights into Ψ distribution and function. **a** Distribution of candidate Ψ sites identified from HeLa cells in different genomic regions. **b** The metagene plot of candidate Ψ sites identified from HeLa cells along the mRNA gene. **c** Numbers of Ψ modification sites harbored by modified transcripts of HeLa cell line. **d** Ψ modification prefers to locate in less structured regions compared to unmodified uridine. **e** Top 10 clusters of GO terms of biological process for moderately and highly modified RNA transcripts.

The 5366 candidate Ψ sites identified from HeLa poly-A RNAs are distributed in 2616 transcripts, of which 82% (4403 sites) are located in messenger RNAs (Fig. 5a), with enrichment in CDS and 3′ UTR and depletion in 5′ UTR, which agrees with previous studies (Fig. 5b)^17^. Most modified transcripts (1621) harbor only one Ψ, although some transcripts are modified at multiple locations, including *FLNA* (19 sites), *A2M* (16 sites), *ATP1A1* (16 sites), *GANAB* (8 sites) and *PRKDC* (10 sites) (Fig. 5c). Importantly, we found that Ψ exhibits higher trend to locate in mismatched and UG or GU wobble paired positions compared to unmodified U sites (Fig. 5d and Supplementary Fig. 18a), which is consistent with the previous report that Ψ installed by PUS7 tends to occur in less structured RNA^36^. This observation is unlikely due to a structural preference of the enzymatic labeling because: (1) we used a protocol that disrupts any remaining RNA secondary structures prior to enzymatic labeling; (2) sites in matched positions in rRNA were as effectively detected as sites in mismatched and wobble positions (Supplementary Fig. 18b); and (3) the enzymatic labeling efficiency on isolated total RNA at 37 °C is the same as that at 65 °C (Supplementary Fig. 18c), although the enzyme activity is higher at 65 °C.

We focused on moderately and highly modified mRNA transcripts for functional analysis. Gene ontology analysis of biological processes and molecular functions suggests a notable correlation of Ψ to cytoplasmic translation, mitochondrial electron transport, and ATP synthesis (Fig. 5e and Supplementary Fig. 18d–f). Mutations in Ψ synthase PUS1 have been linked to mitochondrial myopathy. The identification of Ψ modifications on mRNAs encoding proteins critical for mitochondrial functions, such as UQCRC2, PMPCB, SLC25A5-6, and BNIP3, suggests a direct correlation between pseudouridylation and mitochondrial diseases. Additionally, the link of Ψ to double-stranded DNA break repair is consistent with the previous observation that TruB1 depletion could retard cell growth and cause cell arrest in G1 phase^9^. More studies are required to thoroughly interrogate the determinants of the biosynthesis of Ψ and its biological function.

## Discussion

There are limited tools to study Ψ modifications. Here, we introduce an enzyme-mediated labeling strategy that uses the N1-methyltransferase Mj1640 from *M. jannaschii* to specifically tags Ψ in a simple and mild one-step enzymatic reaction. Although the enzymatic reaction catalyzed by Mj1640 has been reported to only occur at Ψ54 of tRNA in *M. jannaschii*^27^, our biochemical characterization showed that the enzyme converts Ψ to m1Ψ promiscuously in vitro without stringent requirements for RNA secondary structures or specific sequences, while displaying excellent specificity towards Ψ over U. Importantly, it readily adopts propargyl-SeAm as a cofactor to install a propargyl group onto Ψ at position N1, allowing further manipulations through clicking on various functional modules. Based on these results, we developed ELAP-seq, a robust method for enriching RNA fragments bearing Ψ to enable its detection at single-base resolution transcriptome-wide. Although we found that the methyltransferase Mj1640 prefers to methylate Ψ in a 5′ ΨU 3′ context, Ψ in other sequence contexts can still be labeled to lower extents, allowing detection of Ψ across nearly all sequence contexts. The sequence preference difference can be normalized using data of standard oligonucleotides bearing the specific sequence contexts for comparisons of relative intra-group modification variations.

The sites identified by ELAP-seq provided important insights into Ψ deposition and function. One intriguing finding from our data is that Ψ tends to locate in less structured regions. This may be due to the preference of certain Ψ synthases, such as PUS7, catalyzing Ψ formation in less structured regions^36^. Besides, the gene ontology terms of moderately to highly modified sites suggest a close correlation of Ψ to mitochondria function and translation. This has been supported by previous observations that incorporations of Ψ in mRNAs and tRNAs regulate translation in a context-dependent manner^8,37,38^. Considering that Ψ is widely thought to be used by cells to increase their adaptivity to environmental challenges^39,40^, our observations suggest a role for Ψ in providing cells with a mechanism to rapidly alter protein synthesis in response to environmental simulations. These findings call for more in-depth studies in the future.

### Advantages and limitations of ELAP-seq

ELAP-seq, as an enzyme-based strategy for labeling and detecting Ψ, offers a simple, sensitive, and cost-effective method that has the following advantages over other existing methods: (1) The method involves enrichment that substantially enhances the signal-to-noise ratio in the sequencing data. The enrichment for lowly modified and lowly expressed RNAs led to the identification of many modification sites that other methods missed. (2) The sequencing of only modified RNA substantially decreases sequencing and computing cost. For example, our method was able to identify 46% of the sites reported by PRAISE with ~10 times lower sequencing depth. (3) It uses mild labeling conditions avoiding severe RNA degradation, and streptavidin-biotin pair for pull-down, thus doesn’t require a high RNA input compared to other enrichment-based Ψ mapping strategies. Indeed, we were able to build sequencing libraries using 50 times less RNA input compared to CeU-seq.

One limitation of ELAP-seq is its lack of capacity to quantify absolute modification stoichiometry; the current pipeline estimates modification levels by correlating adjusted enrichment levels with the absolute quantification determined by other methods. It is also worth noting that the correlations are stronger for sites with good coverage (i.e., rRNA sites (Fig. 4i), mRNA sites in ΨU context (Supplementary Fig. 15e), and mRNA sites in the non-ΨU context that are readily detectable by CLAP (Fig. 4j)). This suggests that the quantification can be improved by optimization of enzyme sequence context compatibility for better coverage across all sites. Moreover, we envision that including barcoded spike-ins in various sequence contexts with defined modification stoichiometries can improve the current quantification strategy by providing calibration curves that allow direct estimation of modification levels in various sequence contexts. Secondly, ELAP-seq is not suitable for studying sequence motifs of modification sites due to the intrinsic sequence preference of the labeling enzyme. Further engineering the enzyme to increase labeling efficiencies in all sequence contexts would help address this issue. Thirdly, the protocol is less sensitive to detect clustered modification sites like in rRNA. Optimizing the reverse transcription condition for the mutation signature will help mitigate this issue. Lastly, currently the protocol involves both superscript IV and superscript III. In the future, we aim to further optimize the reverse transcription reaction to achieve balanced RT signature and coverage under one single condition.

### Origin of false negatives

Firstly, sequence preferences of enzymatic labeling potentially cause our method to miss sites in less favored sequence contexts, particularly when modification levels or RNA expression are low. Despite the limitation, our method successfully detected 59% of the TruB1 sites identified by PRAISE, which are predominantly found in the 5′ ΨC sequence context (Supplementary Fig. 16f). The sites not detected by our method exhibited weak signals in our dataset but fell below the cutoff threshold, likely due to their low modification or expression levels, making them more difficult to detect. Engineering the methyltransferase to better accommodate various sequence contexts and using higher sequencing depth would help capture more of such sites. Secondly, closely located Ψ modifications could be missed due to the low resolution in the stop signature. Optimizing the reverse transcription conditions to create mutation signatures instead of stops can help address this issue. Lastly, our requirement for stop signals appearing in at least two replicates led to the exclusion of sites captured in only a single replicate. Increasing sequencing depth and the number of replicates will improve detection of these sites.

### Origin of false positives

False positives primarily originate from two sources. First, non-specific pull-down of unmodified RNA fragments (by adhering to magnetic beads) can introduce background noise. To mitigate this, we require RT stop signatures to be reproducibly detected in at least two independent experimental replicates, reasoning that non-specific species are less likely to generate same RT termination signals across independent experiments. Consistent with this reasoning, we observed a decrease in false positives with an increasing number of independent replicates. We also introduced IVT RNA to further eliminate any false positives. Second, although we have performed many quality controls, arbitrary filtering cutoffs commonly employed in sequencing data analysis pipelines can contribute to false positives. Informed by background signals defined by noisy, non-uridine sites, we applied a stricter cutoff for stop ratios to filter our original candidate list for more reliable sites (Supplementary Fig. 14). Taking both factors into consideration, we classified candidate Ψ sites with varying confidence levels based on the number of independent replicates they are detected in and RT stop ratios they have. Lower-confidence sites, although constituting only 10% of all sites, call for caution and further validation. Users may further adjust the cutoffs to suit their research needs.

### Future applications

We anticipate that the enzyme-assisted Ψ labeling strategy will have broader applications beyond probing Ψ modification locations. For example, the high specificity and sensitivity of this labeling strategy make it applicable to low amount of RNAs, allowing development of diagnostic or basic research tools. In addition, pseudouridylation is irreversible, and there has not been a good way to interrogate functions of specific Ψ sites inside cells. The activity of the overexpressed Mj1640 enzyme in cells suggest that this enzyme could be fused to inactive dCAS13 and employed for site-specific labeling of Ψ to m^1^Ψ at endogenous sites. This methodology may provide a promising avenue to connect specific Ψ to biological function and modulate functions of Ψ-modified transcripts if the N1 position is critical to the native role of Ψ. Moreover, the enzymatic treatment may contribute to nanopore sequencing by further decorating Ψ in long, intact RNA with bulky functional groups and allowing enrichment of Ψ-containing transcripts. Additionally, the enrichment of modified RNAs may facilitate identification of interaction partners surrounding the modification, leading to insights into its biological function and biosynthesis. Looking forward, we anticipate the application of ELAP-seq and the enzymatic labeling strategy to advance basic research as well as the development of therapeutic strategies.

## Methods

### Protein purification

The Mj1640 plasmid (deposited in Addgene with deposit ID 87044) for overexpression in E. coli was a kind gift of the Jens Wöhnert laboratory at the Goethe-University Frankfurt. In the plasmid, the Mj1640 gene with an N-terminal His-tag optimized for overexpression in *E. coli* was cloned into the pET11a overexpression vector. The plasmid was expressed in T7 express competent *E. coli* cells (NEB) grown in LB medium and overexpression of the protein was induced by adding IPTG to 1 mM at OD_600_ of around 1 and incubating for 3 h at 37 °C. Two liters of induced cell cultures were pelleted and resuspended in 60 mL lysis buffer composed of 50 mM sodium phosphate (pH 7.5), 500 mM NaCl, 10 mM imidazole, 5 mM beta-mercaptoethanol, and 1 tablet of complete protease inhibitor EDTA-free (Roche). Cells were lysed in French Press with 1–2 rounds of lysis under pressures between 15,000 psi and 20,000 psi. The purified lysate was run through a Ni-NTA column manually, washed with 300 mL wash buffer containing 2.5 mM sodium phosphate (pH 7.5), 12.5 mM NaCl, and 30 mM imidazole, and eluted sequentially with three elution buffers containing 25 mM sodium phosphate (pH 7.5), 125 mM NaCl, and 100, 200, and 300 mM imidazole, respectively. To thoroughly remove bound nucleic acids and contaminating proteins, the eluted protein was further purified sequentially with the anion exchange column twice and Heparin column once. Specifically, proteins were diluted with buffer A (20 mM Tris-HCl, pH 7.4) to lower NaCl concentration to below 75 mM and then loaded onto a FPLC- mono Q or -Heparin column. After washing with buffer A, the proteins were eluted with a gradient mixture of buffer A and buffer B (20 mM Tris-HCl pH 7.4 and 1 M (for the mono Q column) or 1.5 M (for the heparin column) NaCl). The proteins eventually eluted from the Heparin column were buffer exchanged to the storage buffer (20 mM sodium phosphate pH 7.5, 150 mM KCl), concentrated to 20 µL, flash frozen in liquid nitrogen and stored at −70 °C. The resulting protein was > 95% pure.

### In vitro methylation reaction

The condition used for testing the reactivity on synthetic RNA oligos (ordered from Dharmacon) was 15 mM sodium phosphate (pH 7.5), 100 mM KCl, 500 µM SAM or pro-SeAm, 10 µM RNA substrate, and 220 µM enzyme in a 50 µL scale reaction for various time periods at 65 °C. The SAM cofactor was purchased from New England Biolabs, and peo-SeAm was synthesized and HPLC purified by Dr. Mingkui Luo’s lab at Memorial Sloan Kettering Cancer Center, New York. After methylation, synthetic RNAs were purified via phenol-chloroform extraction and ethanol precipitation. The condition used for labeling isolated cellular RNA for sequencing library preparation was 15 mM sodium phosphate (pH 7.5), 100 mM KCl, 321 µM pro-SeAm (added from 1.5 mM stock of Pro-SeAm dissolved in water containing 0.5% TFA (trifluoric acid), with pH adjusted to 7 using Tris-HCl pH 8.3), 12.5% PEG8000, 0.8 U/µl RNaseOut, 0.8 U/µl SUPERase•In™ RNase inhibitor, around 200 ng mRNA (around 70 nM for 200 nt average size) and 64 µM enzyme in 40 µL scale reaction for 2 h at 37 °C. Cellular RNAs were purified using the RNA Clean and Concentrator kit (Zymo Research) and stored in RNase-free H_2_O.

### Dot blot assay

RNA samples after enzymatic labeling and click reaction were in 2 µL water, boiled at 93 °C for 3 min to denature local structure, and then promptly put on ice to avoid reformation of structure. Dotted the desired amount of RNA from each sample on the Amersham Hybond N+ membrane, with 1 µL each round and wait until totally dry before dot again. Crosslinked in auto-crosslinker twice under autocrosslinking mode (70,0000 microJ/cm^2^, 254 nm). Fluorescence signals were directly read with a bioimager. Chemiluminescence signals were detected by the chemiluminescence nucleic acids detection kit. For antibody tests, RNAs without labeling were denatured, dotted, and crosslinked. Then Western blots were performed following the manual of the antibodies.

### MALDI-TOF

Purified RNA oligonucleotides from the enzymatic labeling reaction were resuspended in 5 μL water, mixed with 40 μL ion exchange resin (Bio-Rad), and left at room temperature for 30 min to remove salts. We loaded 1 μL of supernatant of the mixture to a MALDI sample plate, mixed with 1 μL THAP matrix freshly added with salt, and pipetted to mix until crystals were formed. The data was acquired using a Bruker Ultraflextreme MALDI-TOF-TOF mass spectrometer using a negative reflector mode and analyzed using the Bruker FlexAnalysis 3.4 software. More than two biological replicates were analyzed for each sample type.

### Mammalian cell culture and RNA preparations

HEK 293 T cells (purchased from ATCC (cat# CRL-3216)) and HeLa cell lines (purchased from ATCC (cat# CCL-2)) were grown and maintained in DMEM supplemented with 10% FBS and 1% penicillin-streptomycin at 37 °C with 5% CO_2_. Total RNA was extracted from cells using Trizol reagent and poly-A RNA was extracted using Dynabeads® mRNA DIRECT™ Kit for two rounds with an additional ribosomal RNA removal step using the RiboMinus™ Eukaryote Kit v2.

### LC-MS/MS

RNA was digested with 3 μl 10× Reaction Buffer (100 mM NH_4_OAc, pH = 5.3) and 1 μl Nuclease P1 (>1 U/μl) in 25 μl solution at 42 °C for 2 h to break the phosphodiester bond, after which phosphate was removed by adding 1 μl FastAP (1 U/μl) (ThermoFisher, EF0654) and 3 μl 1 M NH_4_HCO_3_ (pH unadjusted), and incubating at 37 °C for 2 h. Upon the completion of incubation, we diluted the reaction mixture to 50 μL with water and filtered the samples through 0.22 μm Millex-GV PVDF filters (Millipore). Five microliters of sample were injected into a ZORBAX SB-Aq 4.6 × 50 mm column (Agilent) on UHPLC (Agilent) coupled to a SCIEX 6500+ Triple Quadrupole Mass Spectrometer in a positive electrospray ionization mode. The nucleosides were quantified based on the nucleoside to base transitions: 268.0–136.0 (A), 284.0–152.1 (U), 245.1–125.0 (Ψ), 259.1–139.0 (m^1^Ψ), and 283.1–163.0 (p^1^Ψ). At least two independent biological replicates were used for Ψ level quantification, and each sample was injected 2 times.

### Primer extension assay

In this assay, a 33-mer synthetic RNA with a Ψ modification in the middle was used as the template. A fluorescently labeled RT primer was designed to target the position immediately downstream of the modification site. The RT primer and template were mixed at a 1:10 ratio to drive the majority of primers to hybridize with the template. In the absence of any treatment, the RT primer reads through the modification site, producing the full-length RT product (upper FL band). Conversely, when encountering the propargyl-Ψ adduct (with or without subsequent biotinylation at a low dNTP concentration), the elongation of the RT primer is impeded, resulting in a significant amount of un-extended RT primer (lower Primer band in Fig. 2e). The limited presence of un-extended RT primer bands in untreated RNAs is likely attributed to a low level of unhybridized RT primer.

### ELAP-seq

#### RNA fragmentation, enzymatic labeling, end repair, and click reaction

RNA was first fragmented using 10× fragmentation buffer (Thermos Fisher) in a 10 µL scale reaction containing 1× fragmentation buffer and 200–1000 ng mRNA. The fragmentation reaction was incubated at 70 °C for 12 min to fragment the RNA to 60–200 nt. RNA was recovered using the Oligo Clean and Concentrator kit (Zymo Research) and eluted in water. For downstream library preparation, the same amounts of mRNA (200 ng) were used in library construction processes. The recovered RNA was denatured at 90 °C for 3 min and quickly set on ice to break RNA secondary structures. One tenth of each RNA sample was aliquoted as input.

The remaining RNA was then labeled in an in vitro methylation reaction using pro-SeAm as co-factor following the protocol mentioned above. RNA was purified using the RNA Clean and Concentrator kit (Zymo Research), eluted in 9 µL of water, and all of it was used in a 50 µL scale end repair reaction containing 1× T4 PNK buffer (NEB B0201S) and 5 µL T4 PNK (Thermos Fisher) with incubation at 37 °C for 1 h. The RNA was then purified using the Oligo Clean and Concentrator kit and eluted in 15 µL water.

We used half of the samples in a 100 µL scale click reaction containing 250 µM biotin-azide, 0.4 mM CuSO_4_, 4 mM THPTA, and 4 mM sodium ascorbate, which was added last. We used the other half in a control reaction where THPTA and CuSO_4_ were omitted. We incubated the reactions at room temperature with shaking at 600 rpm for 1.5 h. RNA was recovered by ethanol precipitation at −80 °C overnight. The input RNA was treated the same way with the end repair reaction above and used directly in RNA ligation.

#### RNA ligation, pull-down, and reverse transcription

RNA recovered from ethanol precipitation was resuspended in 9 µL water and mixed with 1 µL of 30 µM 3′ RNA linker (5′rApp-NNNNNAGATCGGAAGAGCGTCGTG-3SpC3). The mixture was denatured at 72 °C for 2 min and then quickly set on ice. Added to the mixture T4 ligation buffer to 1×, PEG8000 to 15%, 0.8 U SUPERase• In™ RNase Inhibitor, and water to a total of 23 µL. Mixed thoroughly, added two µL T4 RNA ligase 2 truncated KQ and mixed again. Incubated the mixture at 25 °C for 2 h and then 16 °C for 12 h. RNA was then purified with the RNA Clean and Concentrator kit.

Aliquoted MyOne C1 magnetic beads suspension (5 µL per sample) and washed the beads following the manual. Blocked the beads with 2 volumes of 1× W&B buffer containing 0.5 mg/ml BSA and 50 µg/ml tRNA for 1 h at 4 °C. Resuspended the beads in 2 volumes of pull-down buffer containing 10 mM Tris-HCl (pH 7.5), 2 M NaCl, 1 mM EDTA, 8.8 µM biotin-azide, 44 µg/mL yeast tRNA, 0.44 mg/mL BSA, and 0.1% Tween-20. When working with beads, use low-adhesion tubes and tips to minimize loss of beads due to sticking to the tube wall or tips.

Mixed 17 µL purified ligation product with 10 µL bead suspension, and added 15 µL of 0.73× IP buffer (without biotin-azide) to decrease NaCl concentration to 1 M to facilitate biotin-streptavidin binding and decrease PEG concentration to below 5% to avoid bead aggregation. Beads aggregation problem, if met, could be mitigated by adding Tween-20 or DTT when applicable. Incubated the mixture for 1 h at 4 °C and then 30 min at room temperature. Wash the beads 6 times with 100 µL 1× W&B buffer supplemented with 0.05% Tween-20, and then 2 times with 50 µL 1× W&B buffer with each wash incubated at 4 °C for 5 min. Changed tubes every three washes. Changed tubes again after the final wash and resuspended beads in 12 µL 10 mM Tris-HCl buffer (pH 7.5). Beads after pull-down were divided into two parts for the downstream reverse transcription step, with three-quarters (corresponding to 150 ng starting RNA) used for SuperScript III RT and one-quarter (corresponding to 50 ng starting RNA) for SuperScript IV RT.

For input RNA samples, diluted each ligation reaction to 47 µL with water. To remove un-ligated adapters, first add 2 µL 5′ de-adenylase and incubate at 30 °C for 1 h, then add 1 µL RecJ and incubate at 37 °C for 1 h. Purified the RNA using RNA Clean and Concentrator kit and eluted the product in 12 µL water.

Beads resuspended in six µL Tris-HCl buffer from the pull-down experiment or 6 µL purified RNA from input was used in reverse transcription (RT). Set up RT reaction by adding 1 µL 2 mM RT primer (5′-ACACGACGCTCTTCCGATCT-3′), 1 µL 20 µM dNTP and water to 13 µL. For Superscript IV, used 10 mM dNTP instead of 20 µM dNTP. Denatured the mixture in a shaking incubator at 65 °C for 5 min with shaking at 900 rpm and then quickly set on ice for at least 1 min. Added 4 µL 5× SSIV buffer (for the Superscript IV) or 5× first strand synthesis buffer (for the Superscript III), 1 µL 100 mM DTT, 1 µL RNaseOut, and 1 µL Superscript IV or Superscript III reverse transcriptase, and incubated at 50–55 °C for 10 min (for Superscript IV) or 50 min (for Superscript III) with shaking at 900 rpm. After the RT reactions, heat denatured the RT proteins. Then washed beads once with 100 µL high salt buffer composed of 20 mM Tris-HCl pH 7.4, 1 M NaCl, and 0.1% tween-20, and once with 50 µL low salt buffer composed of 20 mM Tris-HCl pH 7.4, 100 mM NaCl, and 0.1% tween-20. Resuspend beads in 18 µL buffer composed of 50 mM Tris-HCl pH 8.3, 75 mM KCl, 3 mM MgCl_2_, and 0.1% tween-20 and denatured the RT enzymes by heating at 80 °C for 15 min. After cooling the reaction on ice, added 1 µL 200 mM DTT and 1 µL RNase H and digested at 37 °C for 30 min with shaking at 900 rpm. Heated the mixture at 72 °C for 2 min and quickly set on the magnet to collect the supernatant. cDNA was purified from the supernatant using Oligo Clean and Concentrator and eluted in 8 µL water.

#### cDNA ligation and PCR

All cDNA was used to mix with 1× T4 RNA ligase buffer, 0.81 µM adapter (5'Phos-NNNNNNAGATCGGAAGAGCACACGTCTG-3SpC3 or 5'Phos-NNNNNAGATCGGAAGAGCACACGTCTG-3SpC3. Details of the specific adapter (with 6N or 5N UMI) used for each library can be found on Github^41^), 1 mM ATP, 24% PEG8000, 7.5% DMSO, and water in a 22 µL scale reaction, and incubated at 72 °C for 2 min before quickly chilling on ice for 2 min. One µL of 50 mM Co(NH_3_)_6_Cl_3_ and 1.8 µL T4 RNA ligase 1 (high concentration) were added and the ligation reaction was allowed to proceed at 25 °C for 12 h, followed by protein denaturation at 65 °C for 5 min. The ligation product was purified with 1.9× Ampure beads and eluted in 10 µL water.

Two µL ligation product was used in qPCR to estimate the number of cycles needed in library amplification PCR. The rest of ligation product was amplified in a 25 µL scale PCR reaction containing LongAmp Taq 1× mix and 0.24 mM forward and reverse primers. To minimize adapter dimers, PCR products of the pull-down samples reverse transcribed using Superscript III were first purified with 0.9× Ampure beads, further amplified for 6 cycles, and then loaded on a 3% low melting-point agarose gel to remove primer dimers. For input samples and pull-down samples built from superscript IV, PCR products were directly run on a 3% low-melting point agarose gel. Control libraries are expected to yield no DNA product under the same PCR cycle numbers as the experimental group. Excise the bands in the desired range and perform gel extraction to recover pure DNA for Illumina sequencing PE150. 25 M reads were obtained from each sample.

#### Library preparation for synthetic RNA oligos containing 5′-NNΨNN-3′ and 5′-NNUNN-3′ sequence

Library preparation for synthetic RNA oligo was performed as above with the following modifications. Synthetic RNA (20 ng) was used in vitro methylation reaction using pro-SeAm as co-factor following the protocol mentioned above. RNA was purified using ethanol precipitation, resuspended in 9 µL of water, and entirely used in a 50 µL scale end repair reaction for incubation at 37 °C for 1 h. The RNA was then purified using ethanol precipitation and resuspended in 9 µL water. One microliter RNA was aliquoted as input.

Both input RNA and RNA for IP were end-repaired and ligated with RNA adapter. After on-bead pull down, reverse transcription was performed on-bead using Superscript IV with 0.5 mM dNTP. The on-bead reaction and wash substantially removed adapter-dimers from the libraries.

### ELAP-seq data analysis

#### Synthetic standard oligo data analysis

Paired end reads were merged using bbmap and deduplicated. 5 and 6 nt were trimmed respectively on the 5′ and 3′ ends to remove UMI with the option of -q 10, 10 to further trim both ends with quality cutoff of 10. Reads of synthetic oligonucleotides were extracted and sorted using the command grep -o “GCTCGCAG.*A” data.fq.gz > output.txt < output.txt sort | uniq -c > output1.txt. Subtotals of number of reads for sequences with identities varying at 2 nucleotides upstream and downstream of the Ψ site were summarized. Enrichment level was determined by dividing the RPM in the pull-down sample to the RPM in the input sample. The heatmap for enrichment levels of all 256 sequences was plotted using R.

#### rRNA data analysis

Data analysis of biological RNA samples can be found on Github^41^. Reads R2 are used for analyzing data of biological RNA. Poly-A RNA ELAP-seq data also contain rRNA reads thus was used for analyzing Ψ modifications in rRNA. Adapter sequence of “AGATCGGAAGAGCGTCGTGTAGGGAAAGAGTGT” was first trimmed from the 3′ end of library R2 of HEK poly-A built with superscript III, followed by deduplication and removing UMI of 6 and 5 nt on the 5′ and 3′ end, respectively. By design, after trimming adapter and UMI sequences, the first nucleotide of R2 is the stop site of reverse transcription. The trimmed reads were mapped to ribosome RNA genome using hisat2 with the options of -k 1 –no-softclip –mp 1000,999 to allow only one distinct alignment, disallow soft-clipping and allow no mismatch. RT arrest was called using JACUSA2 with the options of “rt-arrest -m 0 -p 2 -c 1 -P FR_SECONDSTRAND” to filter off positions with mapping quality <0 or reads coverage <1 and specify second strand sequenced. The resulting output file was processed sequentially with in house scripts rRNA_filter_p1.sh, Stutter_removal_rRNA.py, and rRNA_filter_p2.sh to calculate stop ratios, remove false positive sites due to the stutter effect, and select for sites whose stop ratio is > 0.05 in the pull-down sample. In Stutter_removal_rRNA.py, we required that the stopped reads at a candidate Ψ site must be at least 15% higher than its neighboring sites. Sites called from two replicates were overlapped for stop patterns between independent biological replicates. The resulting sites were further filtered in excel for final candidate sites.

A candidate site in rRNA is defined when meeting the following criteria: (1) the stop ratio at the site in the pull-down library is > 0.05 in both replicates; (2) stop ratio in the pull-down library—stop ratio in the input library is > 0.05 in both replicates; (3) the number of stopped reads in the pull-down library is ≥5 in both replicates; (4) the average value of stop ratio * stopped reads between the two pull-down replicates is ≥1.5 (this imposes a more stringent requirement on the number of stopped reads when the stop ratio in the pull-down sample is lower than 30%); and (5) the stop event occurs at uridine (U). Finally, we further remove sites meeting the following criteria: (1) the stop ratio in the input library is > 0.1 and > 1/3 of the stop ratio in the pull-down library, and (2) number of stopped reads in input is ≥3 in any replicate. When using superscript III and IV data together, we follow the same criteria except for requiring that the stop ratio in the pull-down library- stop ratio in the input library is > 0.1.

#### Poly-A RNA data analysis

Reads R2 from samples generated with superscript III were trimmed 6 nt on the 5′ end and 5 nt on the 3′ end, whereas reads from samples generated with superscript IV was trimmed 7 nt on the 5′ end and 5 nt on the 3′ end as superscript IV tends to elongate for one more nucleotide passing Ψ. Reads after adapter trimming, deduplication, and UMI trimming were mapped to hg38 using hisat2 with options of “–known-splicesite-infile –rna-strandness F –no-softclip”. To combine samples built from superscript III and IV, bam files for the same sample generated from Superscript III and IV were combined to generate a new bam file. We then used MACS2 to call highly enriched peaks and categorized all mapped sites in the pull-down samples into groups inside (in-group) and outside (out-group) of highly enriched peaks. We used JACUSA2.0 to calculate RT-arrest at each site, with parameters “rt-arrest -m 0 -p 2 -c 1 -P FR_SECONDSTRAND”.

We pre-processed the resulting files with the following steps: (1) we filtered away sites in any region covered by reads that all share exactly the same start and end locations, which is likely caused by off-target mapping of reads whose originality is another region in the genome containing a same sequence as the current region; (2) we removed RT stop sites within 50 nt upstream (when reads are mapped to the − strand) or downstream (when reads are mapped to the + strand) of a main RT stop site to decrease false positives due to the effect of diminished processivity of the RT enzyme reading beyond the Ψ modification; and (3) to remove the noise caused by off-target mapping to repetitive regions, we only select for sites that are covered by at least one uniquely mapped read for further analysis.

Next, we determined candidate Ψ sites based on the same principle as used for detecting modifications in rRNA as described above. We raise the stop ratio in pull-down to be higher than 0.1 to decrease potential false positives, considering less crowdedness of modification sites in mRNA compared to rRNA. Sites identified from superscript III alone and new sites identified from combined reads of Superscript III and IV were combined.

Metagene analysis was performed using MetaPlotR^42^. Gene ontology enrichment analysis was done using clusterProfiler^43^.

#### IVT RNA preparation and processing

The protocol reported by Xiao et al. was followed^44^, with the exception that RNase A/T1 digestion was performed in addition to RNaseH digestion after reverse transcription to remove any residual RNA template. The library construction and data analysis followed the same procedures as those used for the biological RNAs. Sites called in low-complexity regions were manually discarded.

#### Validation using CMC-RT-qPCR

Total RNA was treated with Cyclohexyl-N′-(2-morpholinoethyl)carbodiimide (CMC) as previously described^35^. Briefly, 15 or 10 µg total RNA in 12 µl sterile H_2_O was used for the treatment (CMC+) and the control reaction (CMC−). 24 µl TEU buffer (50 mM Tris-HCl (pH 8.3), 4 mM EDTA, 7 M urea), and 4 µl freshly prepared 1 M CMC (C106402-5G, Sigma) in TEU buffer or 4 µl TEU buffer was added to the CMC+ or CMC- tube, respectively. Both samples were incubated at 30 °C for 16 h to ensure complete conversion of Ψ to Ψ-CMC adduct. RNA was then purified with ethanol precipitation. The RNA pellet was then air dried and resuspended in 40 µl of 50 mM sodium carbonate and 2 mM EDTA (pH 10.4) and incubated at 37 °C for 6 h to remove other CMC adducts. RNA was purified again with ethanol precipitation, air dried and resuspended in sterile deionized H_2_O and stored at −80 °C before use. The RNAs were used in RT-qPCR using primers as listed in Supplementary Data 7.

#### Estimation of modification levels

For quantification of modification levels, we used enrichment levels at the modification sites. Enrichment level is defined as the local read coverage per million mapped reads (RPM) value at the site in the pull-down sample divided by that in the input sample. For calculating enrichment levels, two input samples can be combined into one to increase read coverage and to minimize technical variation. For more reliable quantification, we suggest the readers apply a cutoff of 1 for Input-RPM, which corresponds to at least 10 local read coverage under the current sequencing depth (10–20 M mapped reads). Such cutoff can be user-defined based on their sequencing depth and research needs.

We used a synthetic oligonucleotide with sequence of 5′-GGA GAC GGU CGG GΨUC CAG AUA UUC GUA UCU GUC to study the relationship between the enrichment level and the modification percentage. Different ratios of biotin-propargyl-Ψ -modified and unmodified oligos are mixed to make a total of 0.1 pmol comprising 1, 9, 25, 50, and 100% of modified oligos. Pull-down was performed followed by RT-qPCR to determine relative amount of pull-down oligos. By normalizing the enrichment level to the data of modification of 1%, an exponential correlation was found between the modification percentage and the normalized enrichment level: *Y* = 1.879 e^0.0469*X*^, where *Y* is the normalized enrichment level and *X* is the apparent modification level. Based on this result, we propose to estimate the relative modification levels of each modification site using the natural logarithm of the enrichment level at the site adjusted by the preference for the specific sequence context by Mj1640 as described below.

$${{\rm{Relative}}}\; {{\rm{modification}}}\; {{\rm{level}}}={\mathrm{ln}}\left(\frac{{{\rm{Enrichment}}}\; {{\rm{at}}}\; {{\rm{the}}}\; {{\rm{modification}}}\; {{\rm{site}}}}{{{\rm{Enrichment}}}\; {{\rm{of}}}\; {{\rm{the}}}\; {{\rm{oligo}}}\; {{\rm{in}}}\; {{\rm{the}}}\; {{\rm{sequence}}}\; {{\rm{context}}}}\right)$$

Based on the correlations in Fig. 4j (for sites in non-ΨU contexts) and Supplementary Fig. 15e (for sites in ΨU contexts), sites are defined as highly modified when relative modification levels are ≥ 2.3 for sites in ΨU context and ≥ 3.5 for sites in non-ΨU context. Sites are defined as moderately modified when relative modification levels are ≥ 1.6 for sites in ΨU context and ≥ 1.8 for sites in non-ΨU context. Sites are defined as lowly modified when relative modification levels are <1.6 for sites in ΨU context and <1.8 for sites in non-ΨU context.

#### DKC1 knockdown

The day before transfection, 2 × 10⁶ HEK293T cells were seeded onto each 15 cm cell culture plate. Transfection was performed using RNAiMax transfection reagent following manufacturer’s instructions. For one 15 cm plate, 20 μL of 20 μM siDKC1 (Qiagen, Hs_DKC1_6 FlexiTube siRNA) or siCtrl (Sigma Aldrich, catalog # sic001) was used. Cells were harvested 72–96 h post-transfection.

Library construction was performed as previously described, with the following modifications. To minimize variability during the pull-down and downstream processing steps, we designed four RNA adapters containing unique 3-letter internal barcodes (Supplementary Data 8). These adapters were individually ligated to the two siCtrl and two siDKC1 samples, respectively. Following the RNA ligation step, the four input or enzymatically labeled samples were pooled together to ensure uniform handling in subsequent steps.

Paired-end sequencing reads (R1 and R2) were first merged, and samples were then demultiplexed based on their internal barcodes. After deduplication to remove PCR duplicates, the internal barcode and a UMI (8nt in total) were trimmed from the 5′ end, and a second UMI (5 nt) was trimmed from the 3′ end. We used the known modification sites identified from HEK293T cell line as a reference and local read coverage at these sites and on the expected strand (noting strand reversal when applying results from R2-only mapping to the analysis using merged-read mapping) was obtained using JACUSA2 for downstream analysis.

Differential modification was evaluated using changes in the enrichment level defined by local RPM at the site in the pull-down normalized by that in the input. We first selected sites whose enrichment fold change is greater than 2 with *p*-value < 0.05. For downregulated sites, we further excluded sites with average pull-down RPM values < 1 before treatment (corresponding to approximately 10× local read coverage given the current total mapped reads of 6–10 M), as their enrichment efficiencies are more susceptible to technical variation. For more reliable fold-change quantification, readers can apply a cutoff of 1 for Input-RPM under both siCtrl and siDKC1 conditions, which corresponds to at least 10× local read coverage given the current total mapped reads of 10–20 M. Such cutoff can be user-defined based on their sequencing depth and research needs. A more detailed analysis procedure can be found in the GitHub repository associated with this paper.

Differential gene expression analysis was performed on RNA-seq data of input samples for both siCtrl and siDKC1-treated cells using DESeq2.

#### RNA structural analysis

RNA sequences of 100 nt surrounding all Ψ sites located in different mRNA subregions (5′ UTR, 3′ UTR, and CDS) with 50 nt upstream and 50 nt downstream were used for RNA secondary structure prediction. To achieve this, the program ViennaRNA was used with the command of “RNAfold -p <input.fa> output.res”. The resulting file was cleaned up to keep only the information of the RNA sequence and the most confident prediction (the one with lowest ΔG values) and saved as an Excel file. Next, an in-house Python script was used to extract the information of the base pairing status of each modification site as well as the location of the matched base if it is base paired. The identity of the matched base was further determined in the excel using the “MID” function. Next, we calculated the frequency for all modification sites to reside in a mismatch/UG wobble pair versus in UA base pair. Control U sites were chosen at both the −100 position and +100 position of Ψ sites and analysis were performed the same way as for Ψ sites. mfold were used to predict secondary structures of 100 nt surrounding the chosen Ψ sites.

### Statistics and reproducibility

Statistical analysis for siDKC1 knockdown data used one-sided student *t*-test and *p*-values smaller than 0.05 were considered significant. For DKC1-dependent and -independent sites determined by PRAISE, the statistical analysis of these sites in our dataset was performed using a two-sided Wilcoxon rank-sum test. In this paper, error bars represent mean ± standard error of the mean (s.e.m), *n* ≥ 3. No statistical method was used to predetermine sample size, and no data were excluded from the analysis.

### Reporting summary

Further information on research design is available in the Nature Portfolio Reporting Summary linked to this article.

## Supplementary information

Supplementary Information

Description of Additional Supplementary Information

Supplementary Data

Reporting Summary

Transparent Peer Review file

## Source data

Source Data

## Data Availability

The NGS data generated in this paper has been deposited in the GEO database under accession code GSE236530. Source data are provided with this paper.

## Code availability

The code for data analysis can be found on GitHub: https://github.com/yuruwang26/ELAP-seq-v4^41^.
