# Supplementary material for: Enzyme-mediated alkynylation enables transcriptome-wide identification of pseudouridine modifications
Source: Nat Commun. 2026 Mar 23;17:4318. doi: 10.1038/s41467-026-70597-8 (PMC13171889; doi:10.1038/s41467-026-70597-8)
Supplement: Supplementary file 1 [file 41467_2026_70597_MOESM1_ESM.pdf]

Supplementary information

Enzyme-mediated alkynylation enables transcriptome-wide identification of pseudouridine modifications

Yuru Wang, Kinga Pajdzik, Yutao Zhao, Chang Ye, Talbot Stone, Li-Sheng Zhang, Wen Zhang, Mahdi Assari, Wenxin Zhao, Caraline Sepich-Poore, Qing Dai, Ke Wang, Minkui Luo, Tao Pan, and Chuan He

Table of Contents

Supplementary Fig. 1 Purification of recombinant Mj1640 for in vitro characterization 2

Supplementary Fig. 2 LC-MS/MS signals of Ψ, m1Ψ, p1Ψ nucleosides and quantification of enzymatic labeling of Ψ in cellular RNA isolated from HeLa and HEK 293T cell lines 3

Supplementary Fig. 3 Mj1640 uses a mild condition to label RNA and can also label endogenous Ψ inside cells.. 4

Supplementary Fig. 4 Characterization of the enzymatic labeling assay 5

Supplementary Fig. 5 ELAP-seq for 5'-NNUNN control synthetic RNA oligonucleotides 6

Supplementary Fig. 6 MALDI-TOF of 10- or 11-mer oligonucleotides... 7

Supplementary Fig. 7 Detection of rRNA Ψ sites by ELAP-seq... 8

Supplementary Fig. 8 Pipelines to analyze ELAP-seq data of mRNAs. 9

Supplementary Fig. 9 ELAP-seq identified thousands of candidate Ψ sites in human transcripts........... 10

Supplementary Fig. 10 Evaluation of reproducibility of ELAP-seq... 11

Supplementary Fig. 11 ELAP-seq analysis on IVT RNA 11

Supplementary Fig. 12 Differential modification upon DKC1 knockdown.. 12

Supplementary Fig. 13 Validation of candidate modification sites by CMC-RT-qPCR 13

Supplementary Fig. 14 Categorizing candidate Ψ sites into different confidence levels 14

Supplementary Fig. 15 Semi-quantitative evaluation of Ψ modification levels...................................... 15

Supplementary Fig. 16 Comparison of ELAP-seq to other methods.. 16

Supplementary Fig. 17 Mitochondrial mRNA modification sites 18

Supplementary Fig. 18 Ψ detected by ELAP-seq provides new insights into the biogenesis and functions of Ψ 19

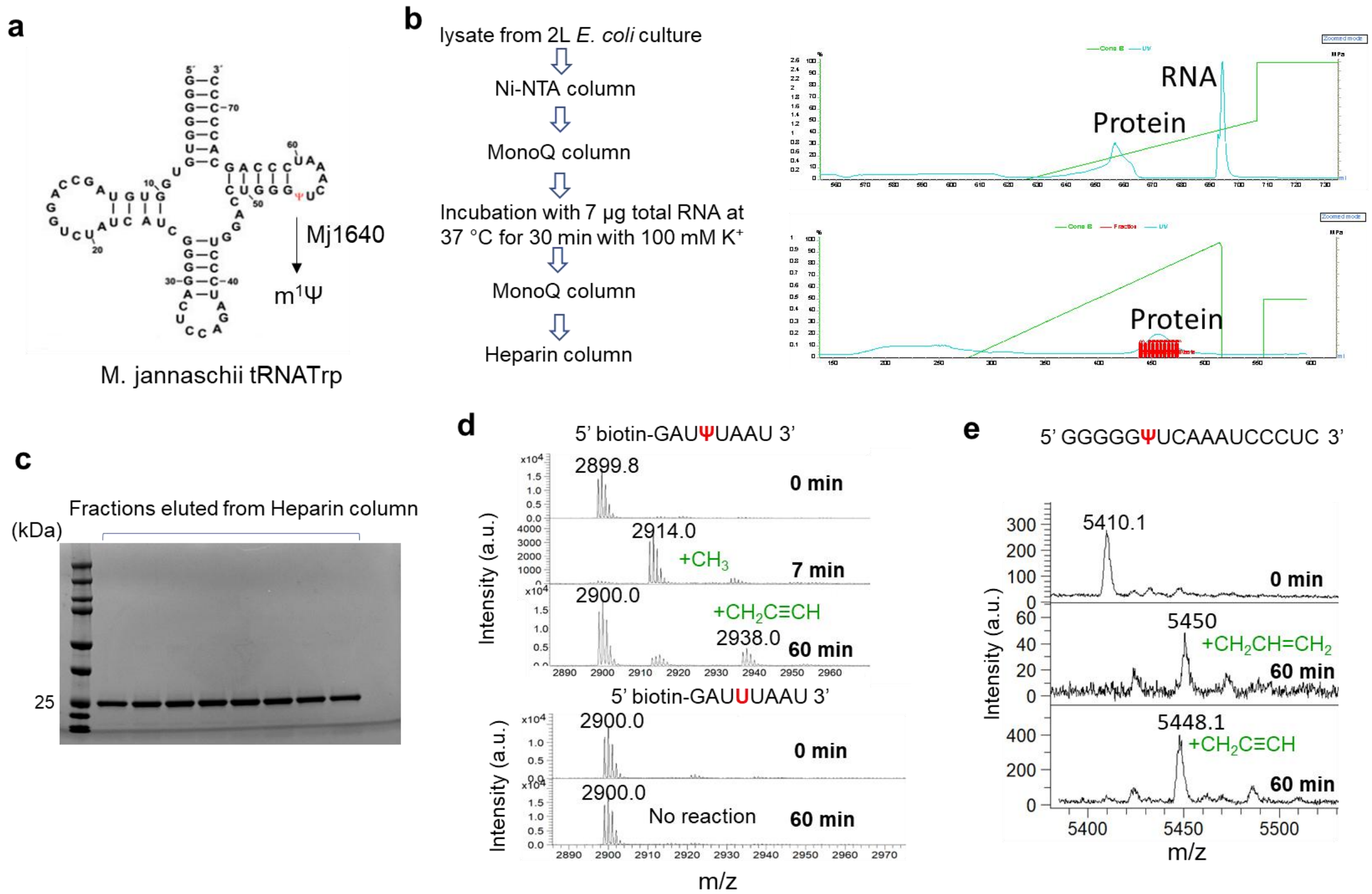

Supplementary Fig 1. Purification of recombinant Mj1640 and *in vitro* characterization. a) Naturally occurring reaction catalyzed by Mj1640 on M. jannaschii $tRNA^{Trp}$. b) The workflow to purify the recombinant Mj1640 (left) and HPLC trace of Mono Q (top) and Heparin (bottom) chromatography (right). c) SDS-PAGE analysis of fractions eluted from the last chromatography in the workflow. d) MALDI-TOF shows that the recombinant Mj1640 labels an 8-mer RNA oligonucleotide containing a Ψ in the center but not a counterpart RNA containing a U. e) MALDI-TOF shows that the recombinant Mj1640 can efficiently and specifically label a 17-mer Ψ-RNA oligonucleotide with an allylic (middle) or a propargyl (bottom) group.

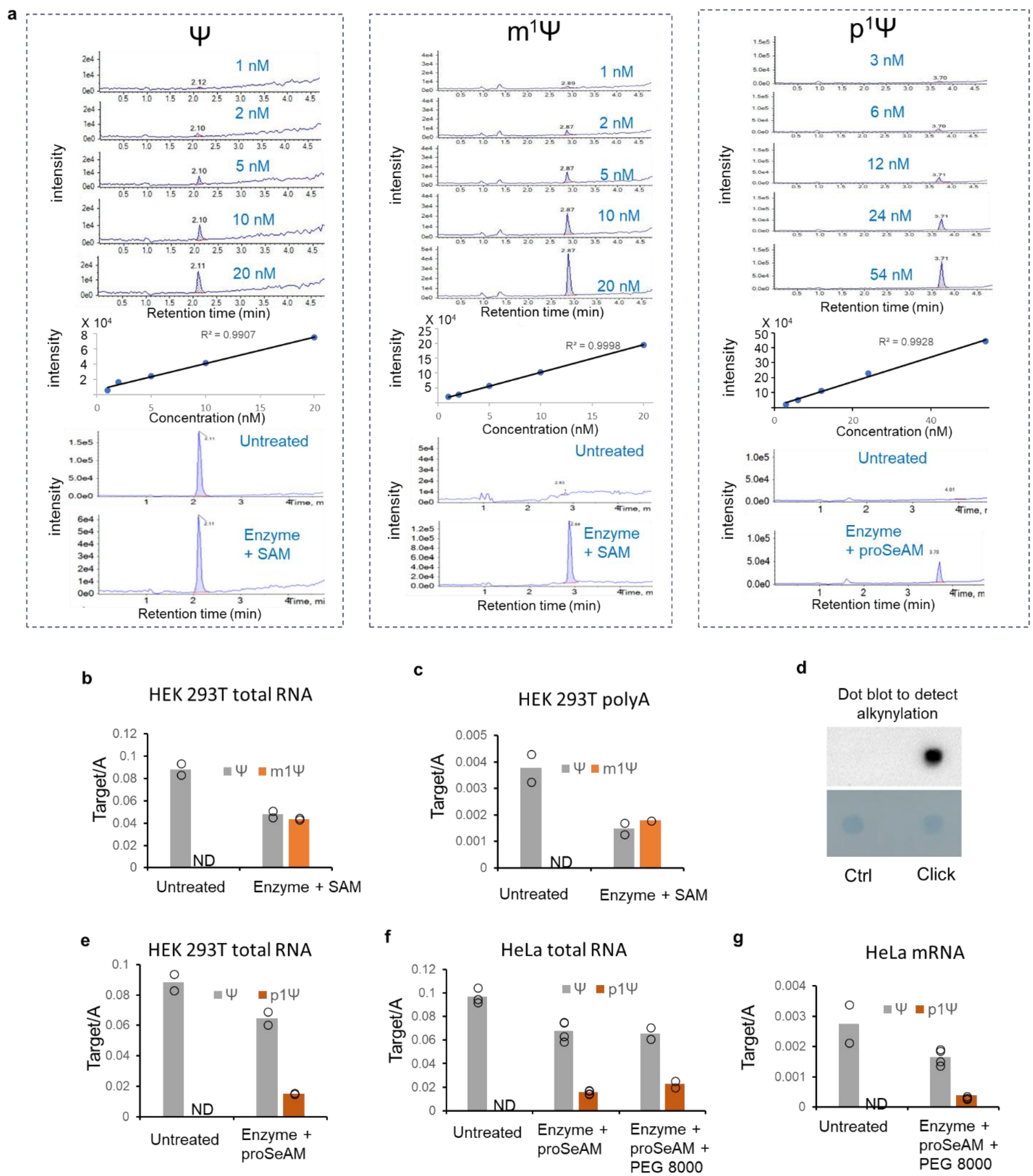

Supplementary Fig 2. LC-MS/MS signals of Ψ, m^1Ψ, p^1Ψ nucleosides and quantification of enzymatic labeling of Ψ in cellular RNA isolated from HeLa and HEK 293T cell lines. a) LC-MS/MS signals and calibration curves used to quantify the efficiency of enzymatic addition on Ψ in cellular RNA with methyl and propargyl groups. b) Methylation efficiency on Ψ in total RNA extracted from HEK 293T cells. n=2 independent experiments. c) Methylation efficiency on Ψ in poly-A RNA extracted from HEK 293T cells using two rounds of poly A selection. n=2

independent experiments. d) Dot blot to detect alkynylation through the enzymatic labeling followed by the addition of a biotin molecule via copper-catalyzed azide–alkyne cycloaddition (CuAAC or click) reaction, which is then detected using the chemiluminescent nucleic acids detection kit (Thermo Fisher). e) Propargyl group labeling efficiency on Ψ in total RNA extracted from HEK 293T cells. n=2 independent experiments. f) Optimization of enzymatic labeling conditions. Adding PEG8000 effectively increases the labeling efficiency. Untreated: n=3 independent experiments. Enzyme+ proSeAm: n=3 independent experiments. Enzyme + proSeAm + PEG 8000: n=2 independent experiments. g) Propargyl group labeling efficiency on Ψ in mRNA extracted from HeLa cells using two rounds of poly A isolation and one round of ribosomal RNA depletion. Untreated: n=2 independent experiments. Enzyme + proSeAm + PEG 8000: n=4 independent experiments.

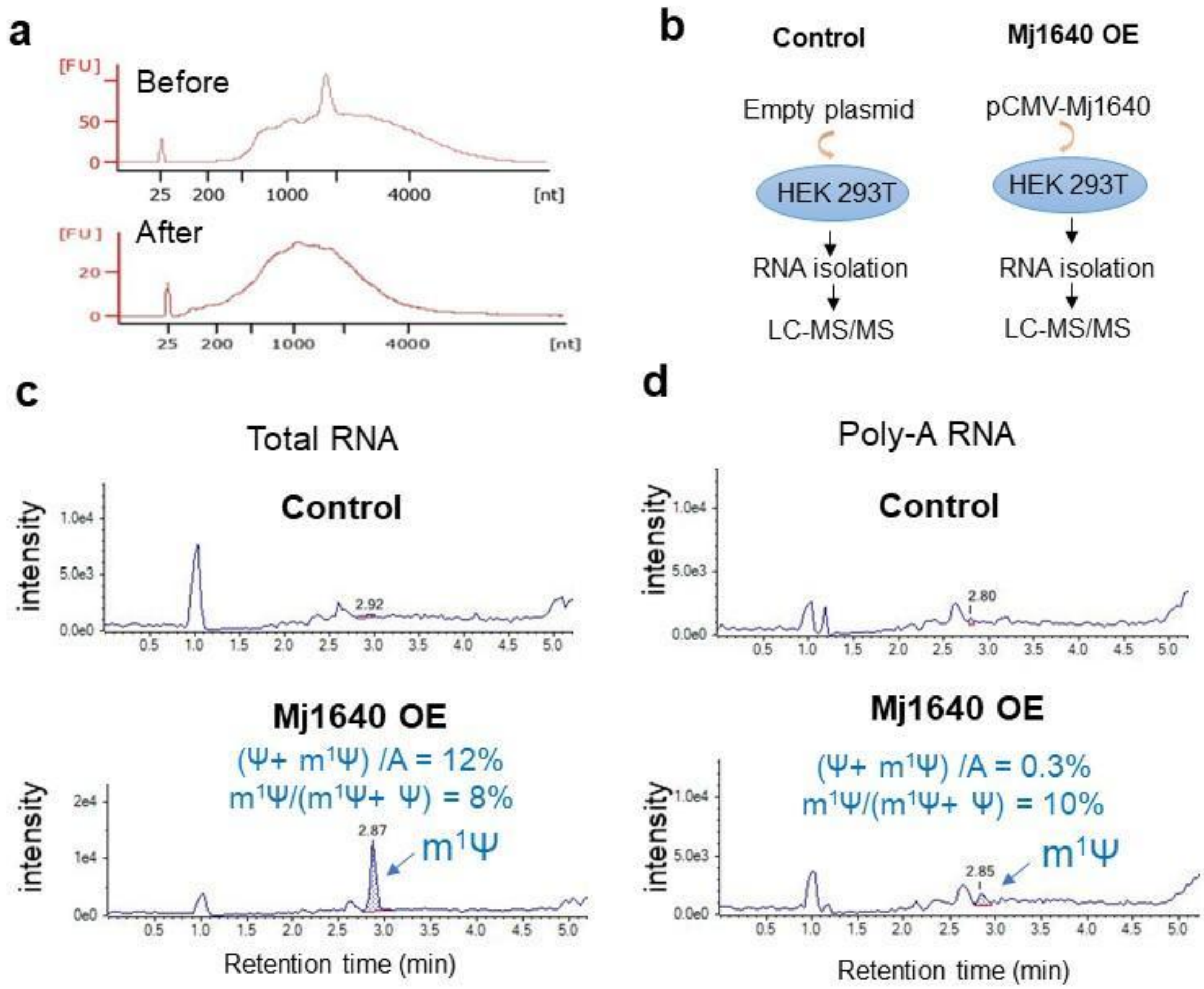

Supplementary Fig 3. Mj1640 uses a mild condition to label RNA and can also label endogenous Ψ inside cells. a) Isolated mRNA remains largely intact after the enzymatic treatment using Mj1640 *in vitro*. b) Experiments to test the activity of exogenously expressed Mj1640 inside human cells. c) LC-MS/MS trace shows that Mj1640 overexpressed in HEK 293T

cells successfully labeled 8% of endogenous Ψ in total RNA and d) 10% of endogenous Ψ in polyA RNA. n=1 experiment for c) and d).

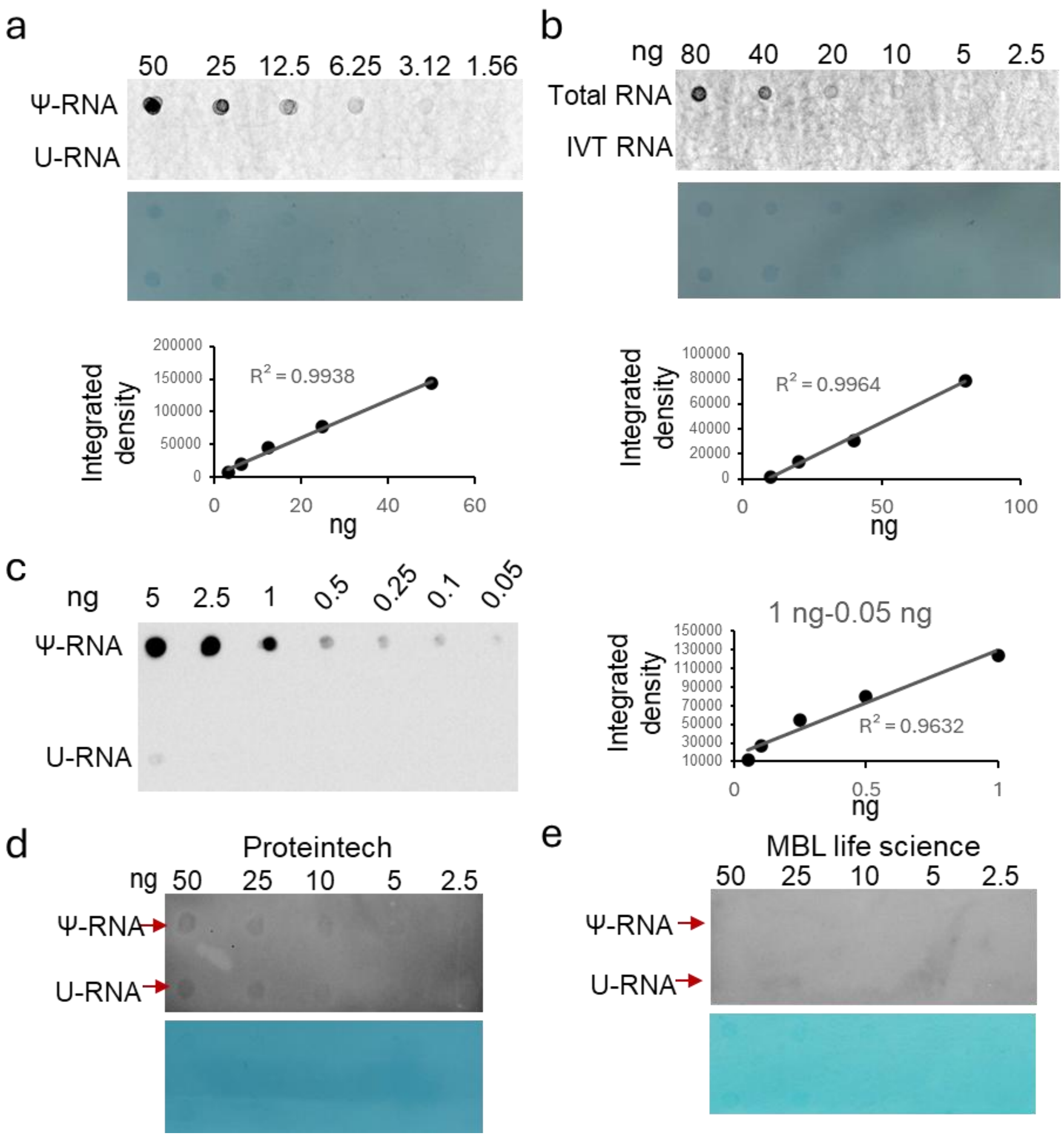

Supplementary Fig 4: Characterization of the enzymatic labeling assay. (a) Fluorescence signals from modified (Ψ-RNA) and unmodified synthetic RNA (U-RNA) in a degenerate sequence context (NNUNN) that were enzymatically labeled and clicked with Fluor 488. (b) Fluorescence signals from total RNA isolated from HEK cells and IVT RNA representing the HEK transcriptome and free of modifications, both labeled and clicked with Fluor 488. (c) Chemiluminescence signals from modified and unmodified synthetic RNAs that were labeled and clicked with biotin. Chemiluminescence signals were detected based on the biotin-

streptavidin interactions using the Chemiluminescence Nucleic Acids Detection Kit (ThermoFisher Scientific). The trace signal with the U-RNA may originate from unremoved free biotin contamination or bacterial RNA contamination in the purified protein. Methylene blue staining was omitted due to low RNA input. (d) Chemiluminescence signals from modified and unmodified synthetic RNAs probed with commercial antibodies (proteintech cat# 68578-1-Ig and MBL life science Code No. D347-3). The methylene blue staining signals were weak because the membrane was masked during blocking. The experiments were repeated at least once with similar results.

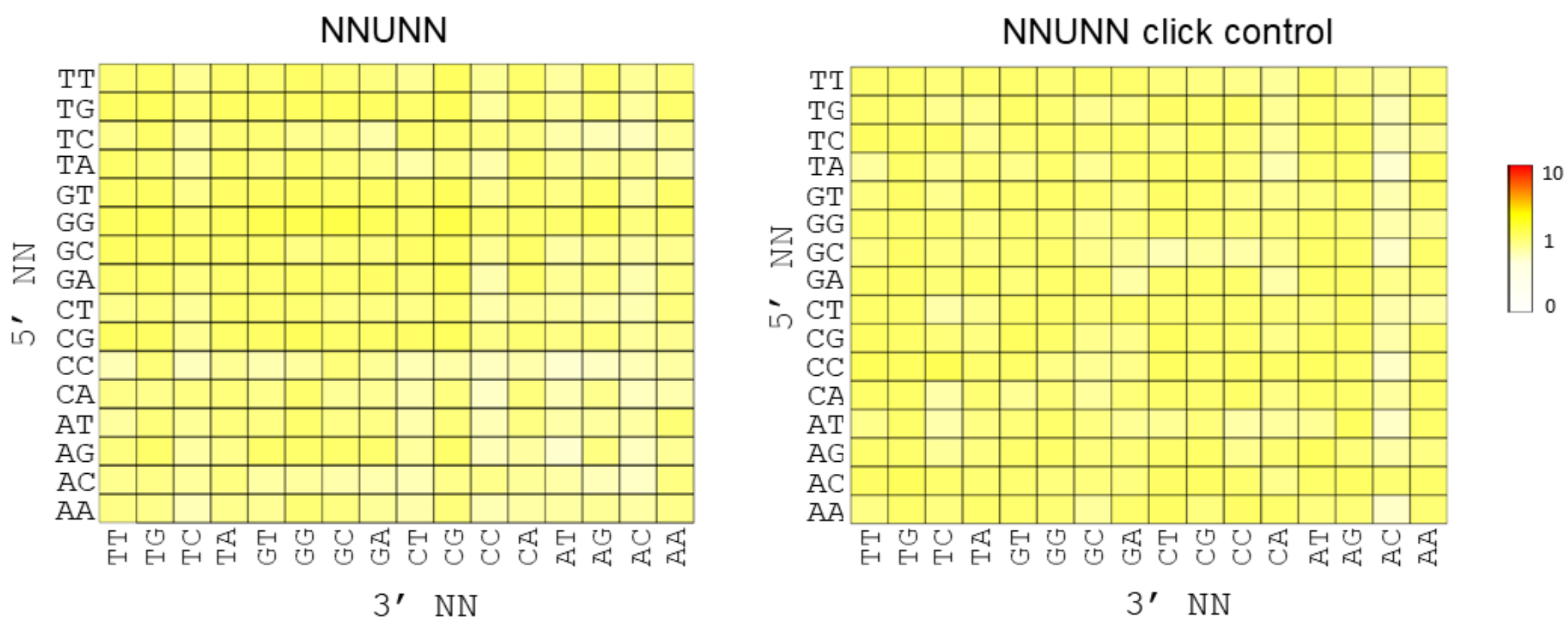

Supplementary Fig 5. ELAP-seq for 5'-NNUNN control synthetic RNA oligonucleotides. Heatmaps show degrees of enrichment for control synthetic 5'-NNUNN RNA oligonucleotides (left), and its click control library (right, omitting Cu^{2+} in the click reaction) covering all 256 sequences. No specific sequence was enriched for the NNUNN spike-in, like its own click control library. Compared with the NNΨNN libraries, both the NNUNN and its click control libraries were built with overamplification.

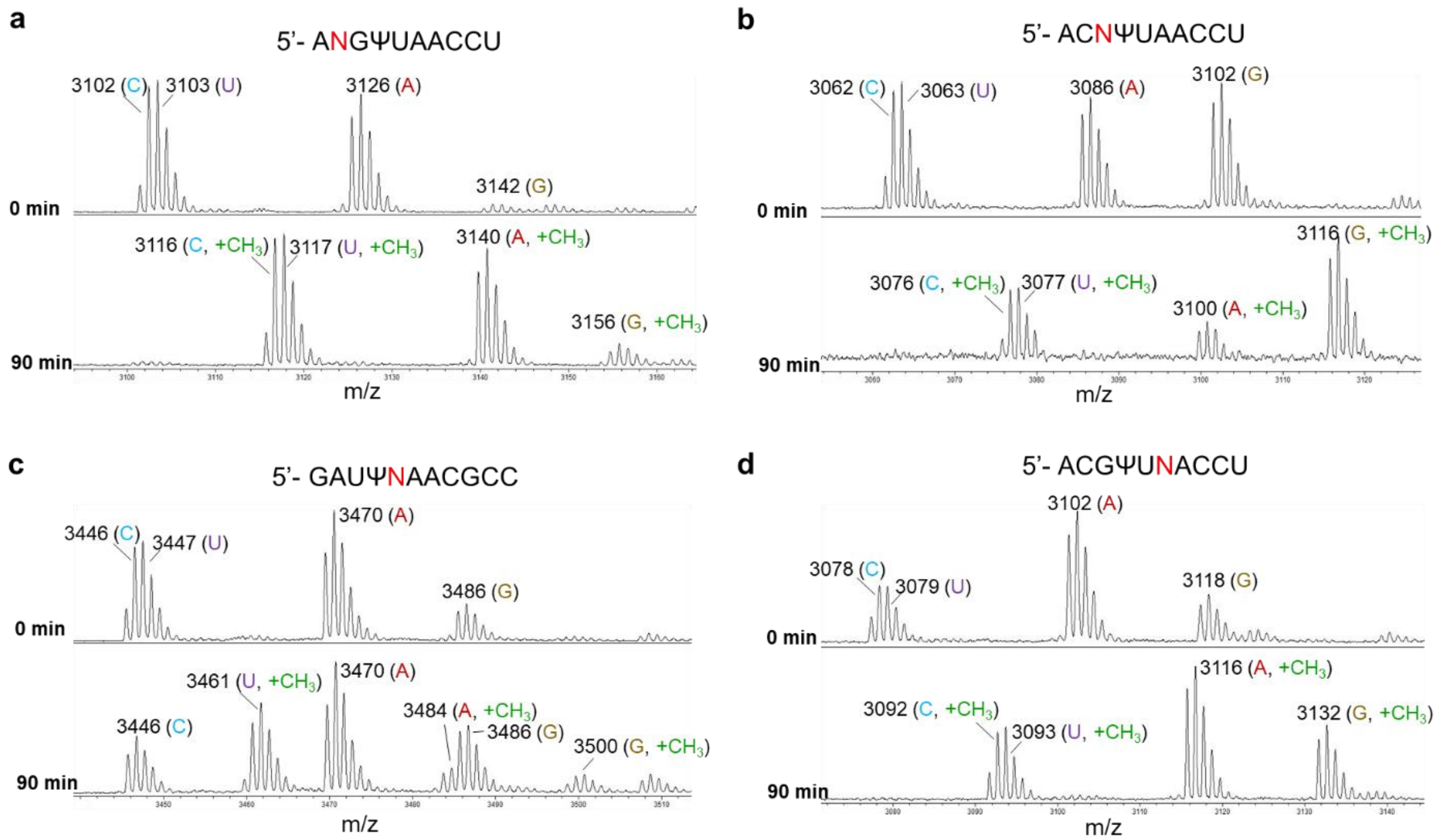

Supplementary Fig 6. MALDI-TOF of 10- or 11-mer oligonucleotides. The experiment tests Mj1640's sequence preference for nucleobase at positions -2 (a), -1 (b), +1 (c), and +2 (d) relative to the Ψ modification. The experiment was repeated at least once with similar results.

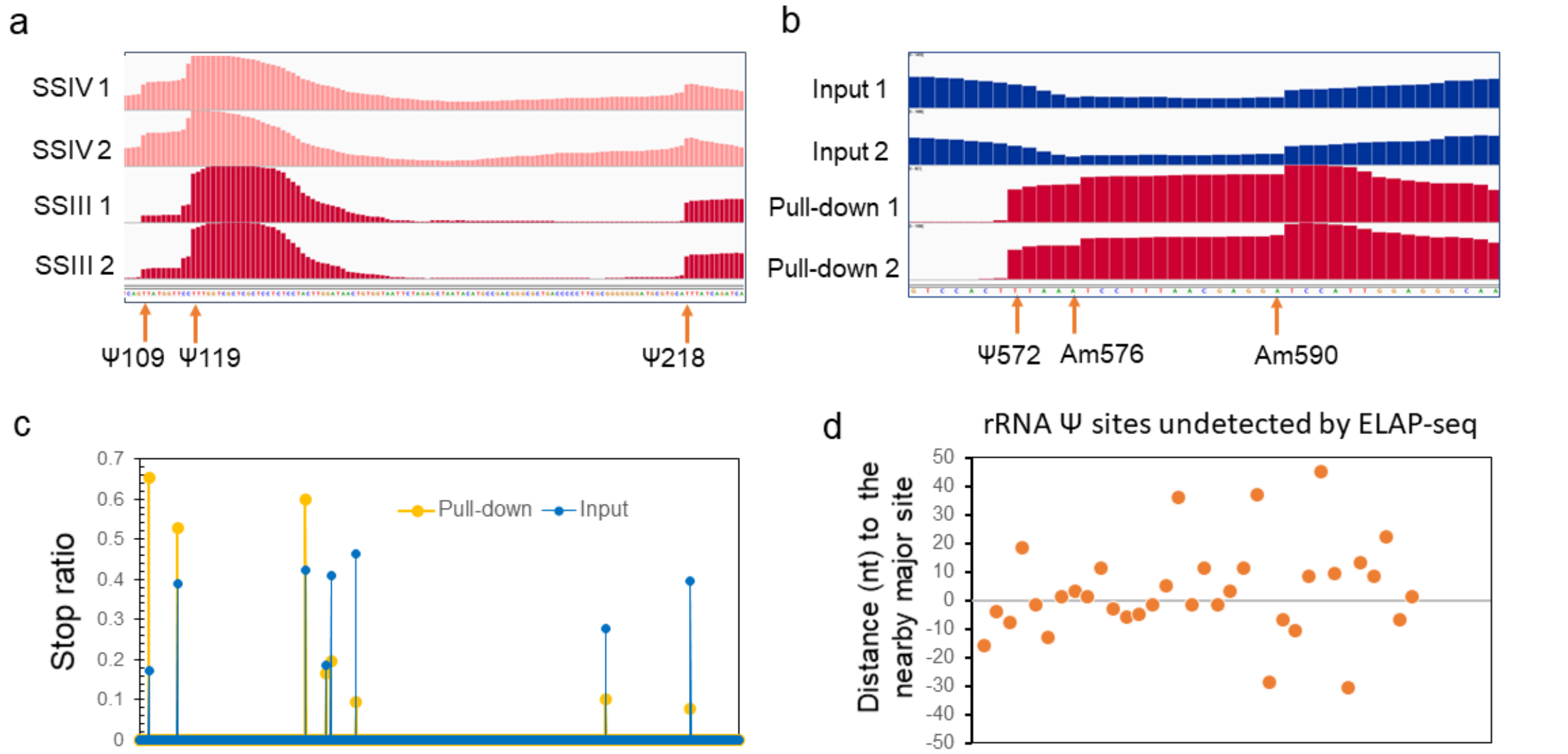

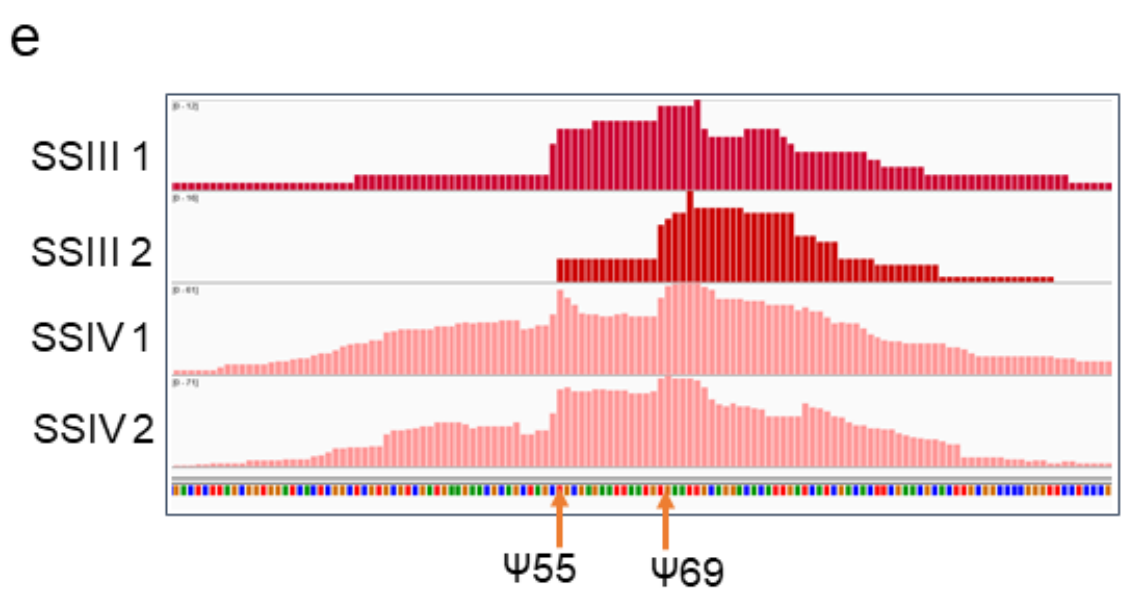

	SSIII 1	SSIII 2	SSIV 1	SSIV 2
Stopped reads at Ψ55	2	4	16	19
Stop ratio at Ψ55	0.5	1	0.31	0.39
Stopped reads at Ψ69	2	6	12	10
Stop ratio at Ψ69	0.29	0.6	0.26	0.18

Supplementary Fig 7. Detection of rRNA Ψ sites by ELAP-seq. a) Stop signatures generated by Superscript IV (SSIV) and Superscript III (SSIII). (b) Stop signatures at Ψ are generated only after enzymatic treatment whereas Nm signatures exists in both the input and the pull-down samples. c) Nm signatures on human 18S rRNA. The stop signatures exist in both the input and the pull-down samples thus can be distinguished from Ψ signatures. d) rRNA sites not detected by ELAP-seq are mostly clustered (located within 20 nucleotides (nt) from a nearby modification site). e) SSIV data identified two sites on 5.8S missed by SSIII.

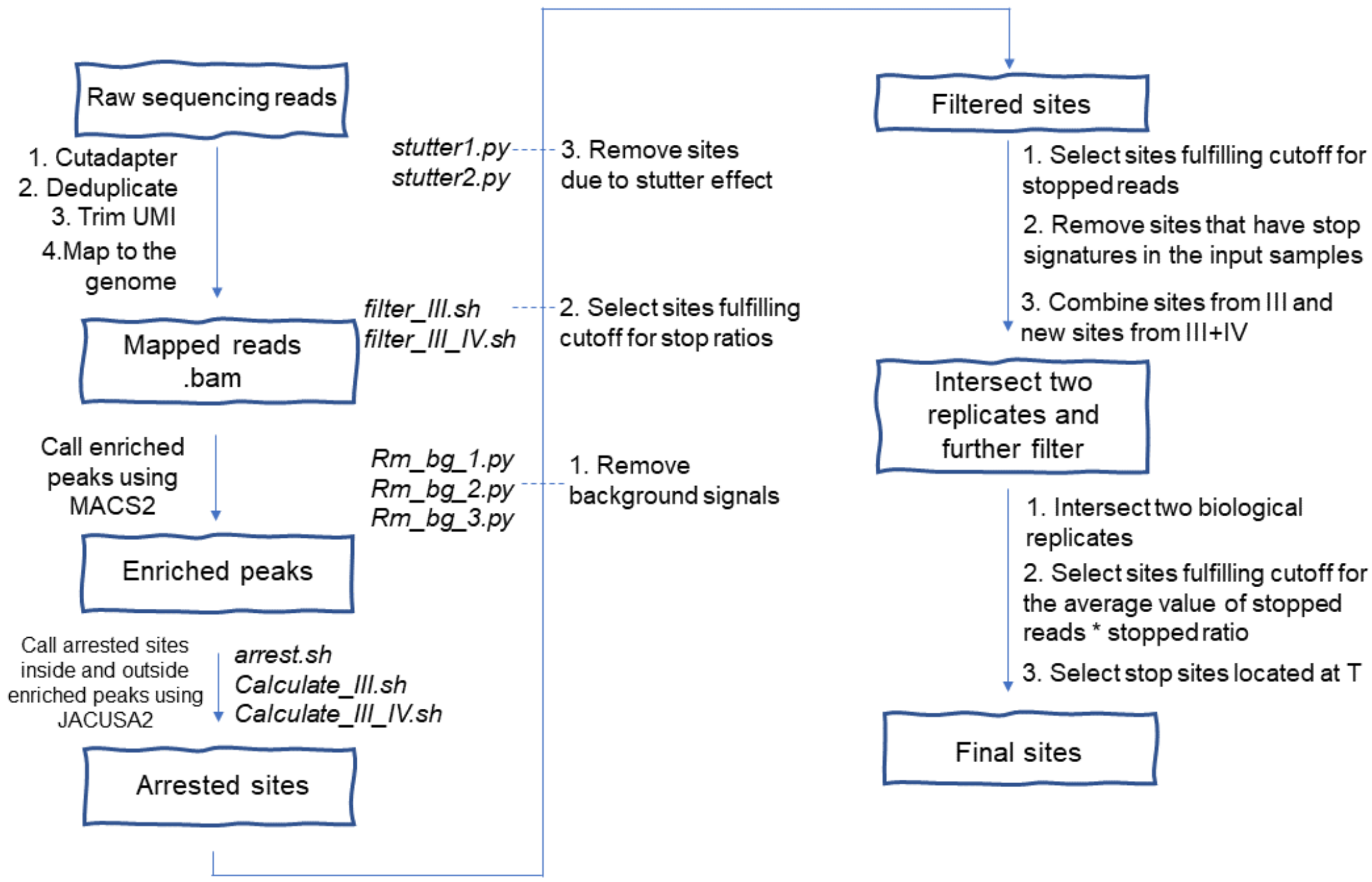

Supplementary Fig 8. Pipelines to analyze ELAP-seq data of mRNAs.

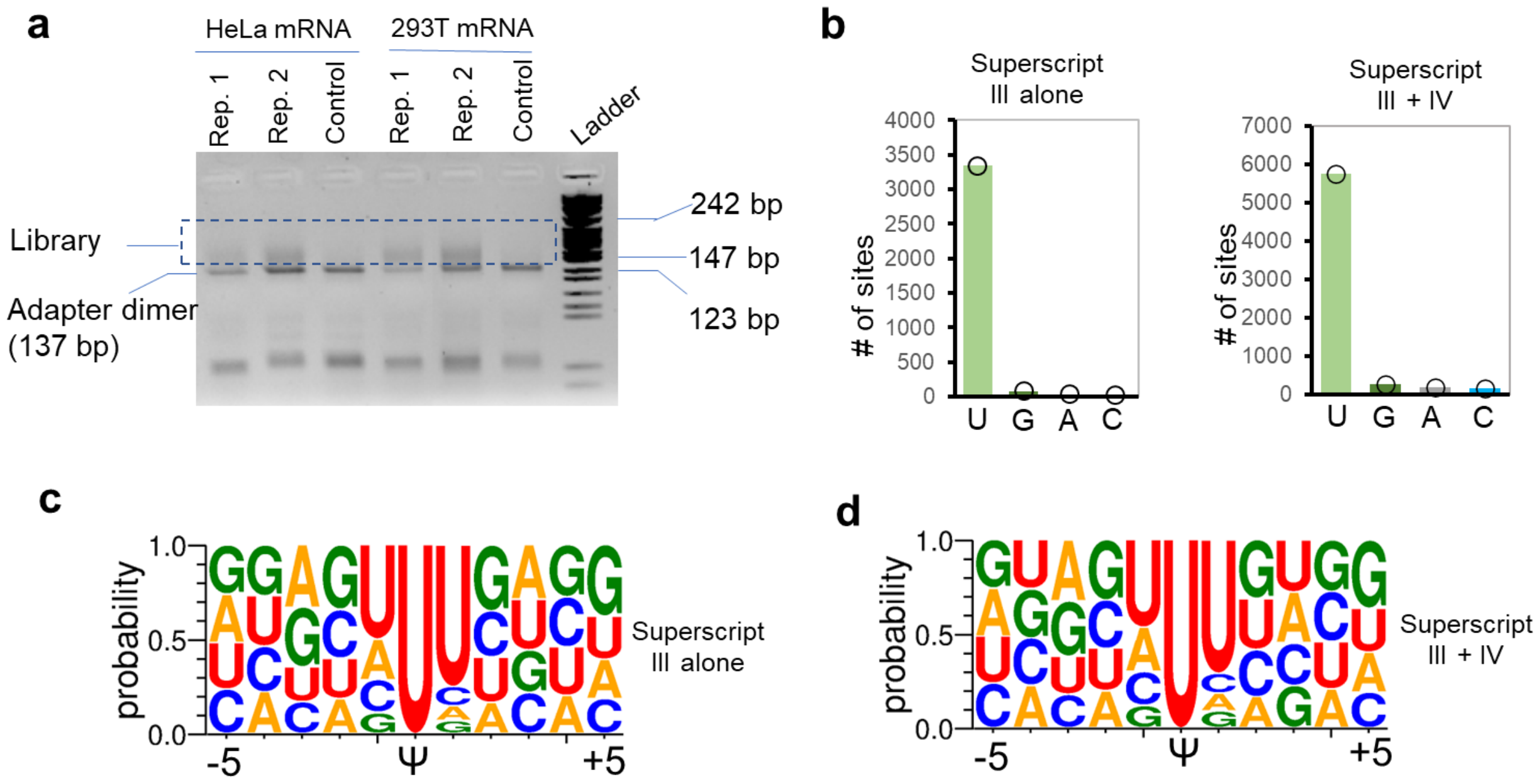

Supplementary Fig 9. ELAP-seq identified thousands of candidate Ψ sites in human transcripts. a) The agarose gel of the final PCR products in the process of constructing libraries for Illumina sequencing. b) Base distribution of overlapped stop sites identified from two replicates of poly-A RNA extracted from HEK 293T cell lines using reads of libraries generated with Superscript III alone or combined reads of libraries generated with Superscript III and Superscript IV. n=1. c) Sequence motif of 11-nt surrounding the candidate Ψ sites of HeLa identified from libraries generated with Superscript III alone. d) Sequence motif of 11-nt surrounding the candidate Ψ sites of HeLa identified from combined sequencing reads of libraries generated with Superscript III and Superscript IV.

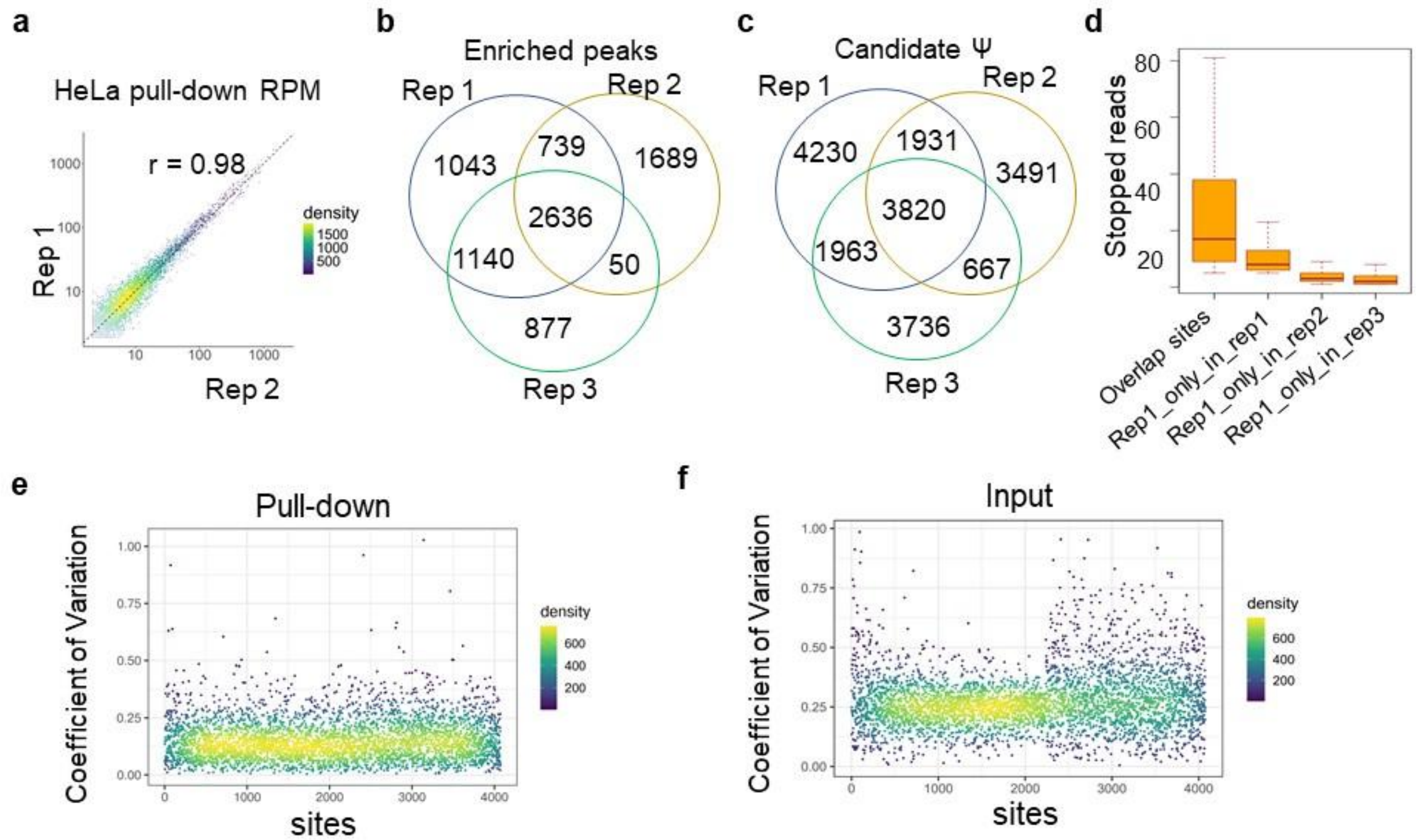

Supplementary Fig 10. Evaluation of the reproducibility of ELAP-seq. a) Correlation of RPM values between two replicates for input and pull-down samples. b) Overlap of pull-down peaks called by MACS2 in HEK293T cells among the three replicates. c) Overlap of RT stop sites at U identified in HEK293T for each replicate. d) Comparison of the number of stopped reads between sites identified in at least two replicates ("overlap sites") and sites identified in only replicate 1. Only sites identified in at least two replicates are defined as candidate modification sites. e-f) Coefficient of variation of RPM values in pull-down (e) and input samples (f) across three replicates for candidate sites shared by all three replicates.

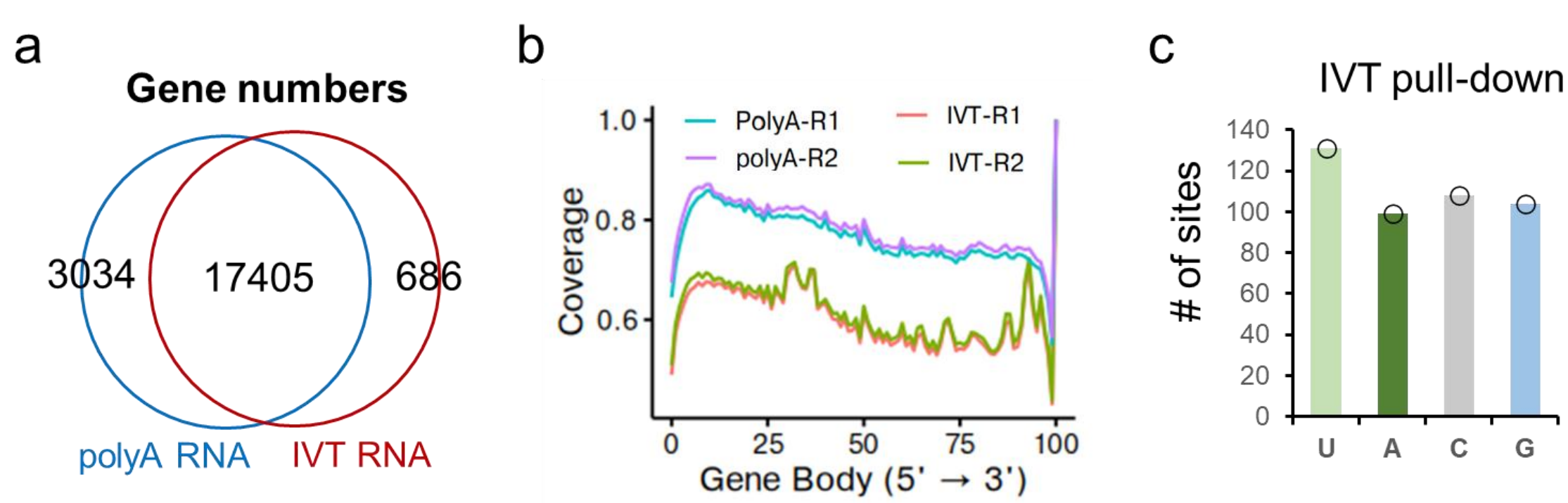

Supplementary Fig 11. ELAP-seq analysis on IVT RNA. a) Venn diagram depicting gene overlap between polyA RNA and IVT RNA input samples. b) Read coverage from the 5' end to the 3' end of transcripts in polyA RNA and IVT RNA input libraries. c) Base distribution of overlapped RT arrest sites identified from two IVT RNA libraries. n=1.

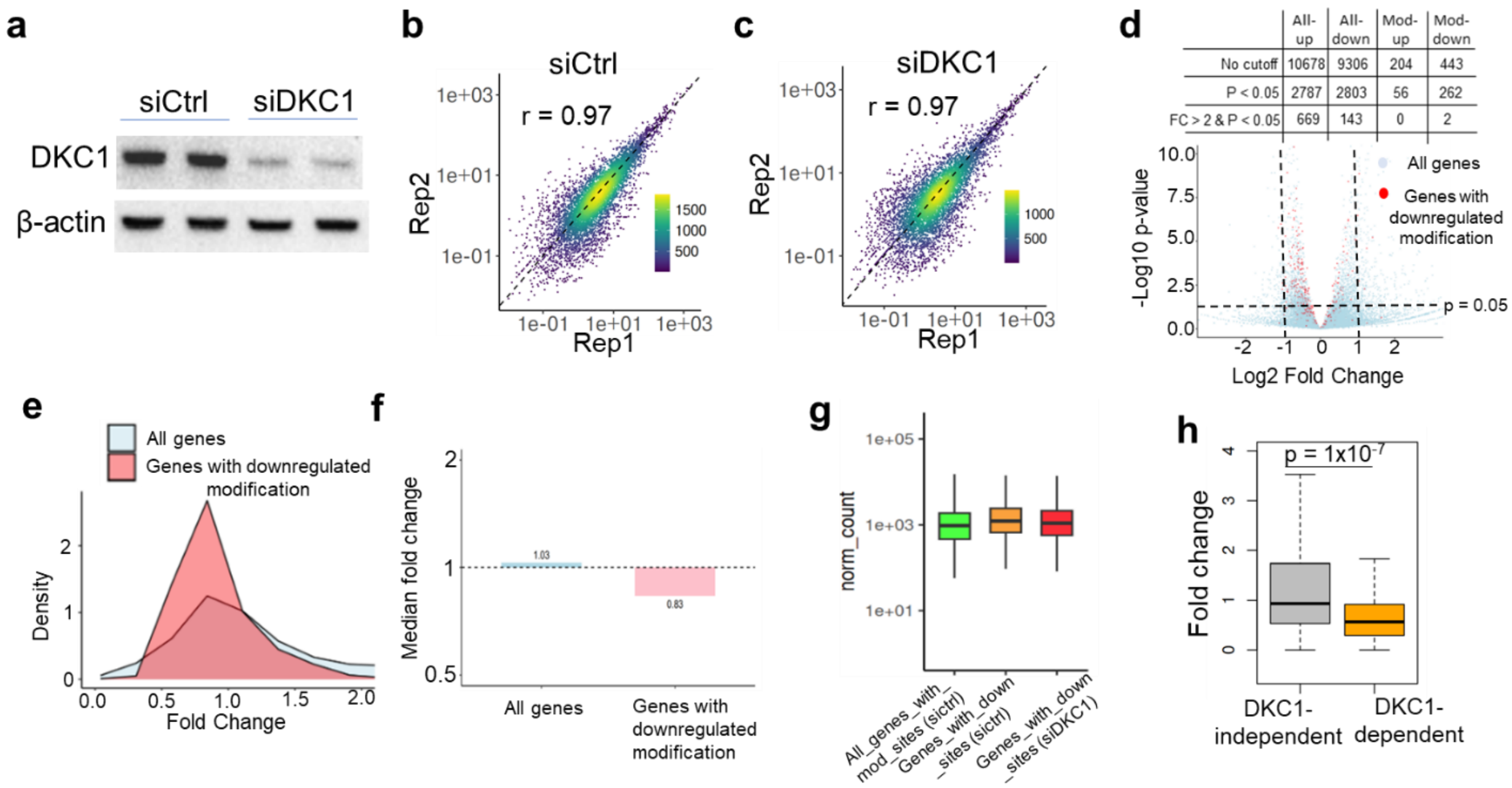

Supplementary Fig 12. Differential Ψ modification upon DKC1 knockdown. a) Western blot showing the successful knockdown of the DKC1 protein in HEK 293T cells (siDKC1) compared to the control (siCtrl). b) Enrichment values for Ψ sites show excellent correlation between two biological replicates for both siCtrl and c) siDKC1 samples. d) Differential gene expression analysis between siCtrl and siDKC1 conditions using the input libraries. Shown are all genes detected by RNA-seq (light blue) and genes harboring Ψ sites identified as downregulated upon DKC1 knockdown (red). All_up and All_down denote all genes whose expression is upregulated or downregulated in siDKC1 relative to siCtrl, respectively; Mod_up and Mod_down denote genes harboring downregulated Ψ sites whose expression is upregulated or downregulated, respectively. e) Distribution of gene expression fold changes following siDKC1 treatment (without filtering by p-value or fold-change threshold) for all genes and for genes harboring downregulated Ψ sites. f) Median fold changes in gene expression (without filtering by p-value or fold-change threshold) for all genes and for genes harboring downregulated Ψ sites. Panels (d–f) show that although genes harboring downregulated Ψ sites tend to exhibit modest expression decreases upon DKC1 knockdown, the magnitude of these changes is mild and unlikely to affect the reliability of the detected Ψ downregulation events. g) Box plots showing the distribution of normalized read counts for genes harboring downregulated Ψ sites before knockdown (orange box), after knockdown (red box), and for all genes harboring Ψ sites prior to knockdown (green box). Considering all genes harboring Ψ sites detected before knockdown represent transcripts with sufficient expression levels to support reliable pull-down, the comparable read count distributions indicate that post-knockdown expression levels of genes harboring downregulated Ψ sites remain within a range for efficient pull-down. h) DKC1-dependent sites determined by PRAISE showed an overall downregulation in the ELAP-seq dataset compared to the DKC1-independent sites defined by PRAISE. Fold change means enrichment level. The P value from two-sided Wilcoxon rank-sum test is shown.

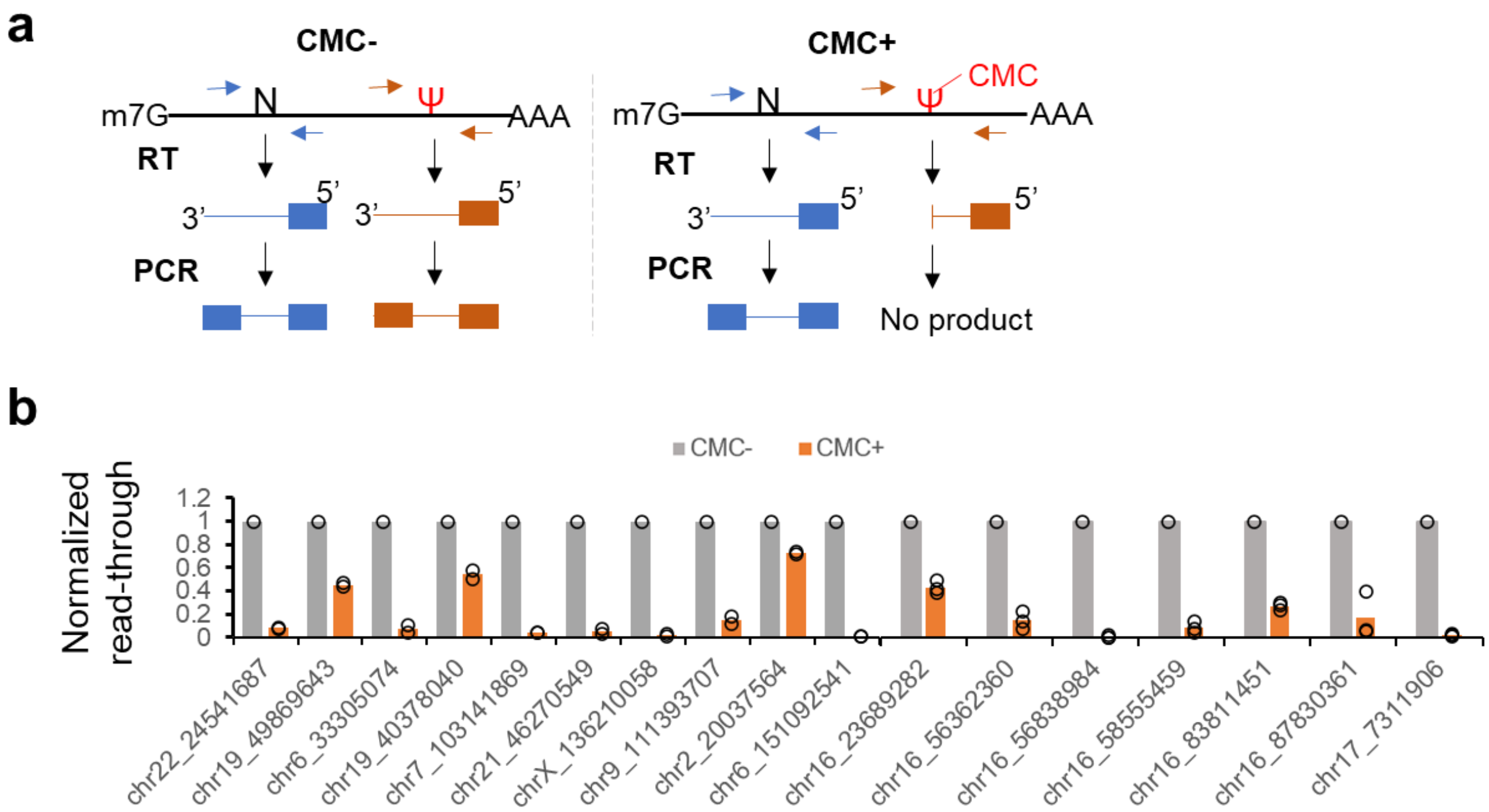

Supplementary Fig 13. Validation of candidate modification sites by CMC-RT-qPCR. a) The scheme of using CMC-RT-qPCR. b) 17 candidate sites are successfully validated by CMC-RT-qPCR. n=3.

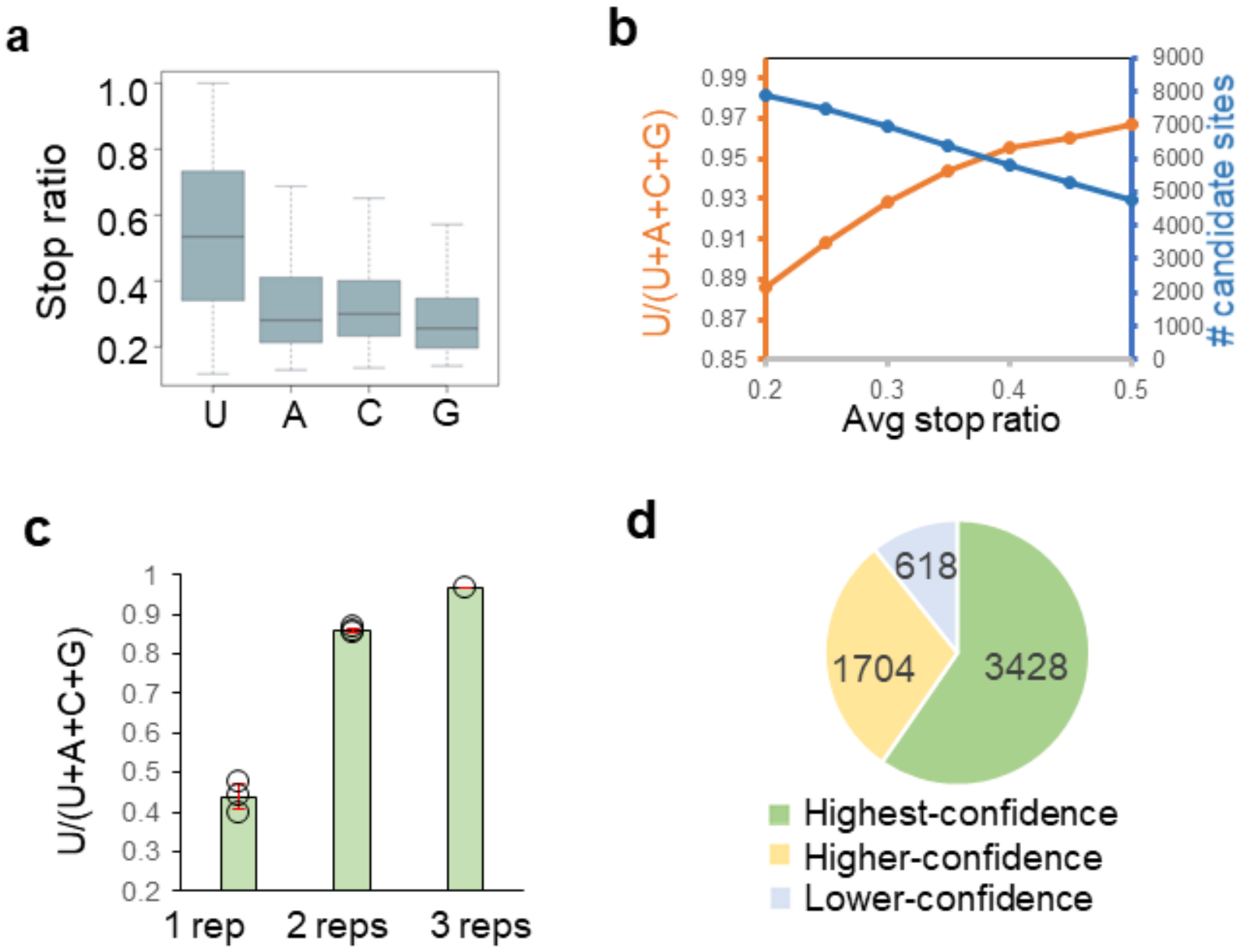

Supplementary Fig 14. Categorizing candidate Ψ sites into different confidence levels. a) Box plot showing that U sites displayed higher stop ratios than non-U sites identified by ELAP-seq. b) The percentage of U increases and the number of identified sites decrease with increasing stop ratio cutoff. c) Higher number of replicates leads to higher confidence level. For "1 rep", n=3. For "2 reps", n=3. For "3 reps", n=1. d) Categorizing candidate Ψ sites into different confidence levels based on a more stringent threshold for stop ratio and the number of replicates a site can be repeatedly identified.

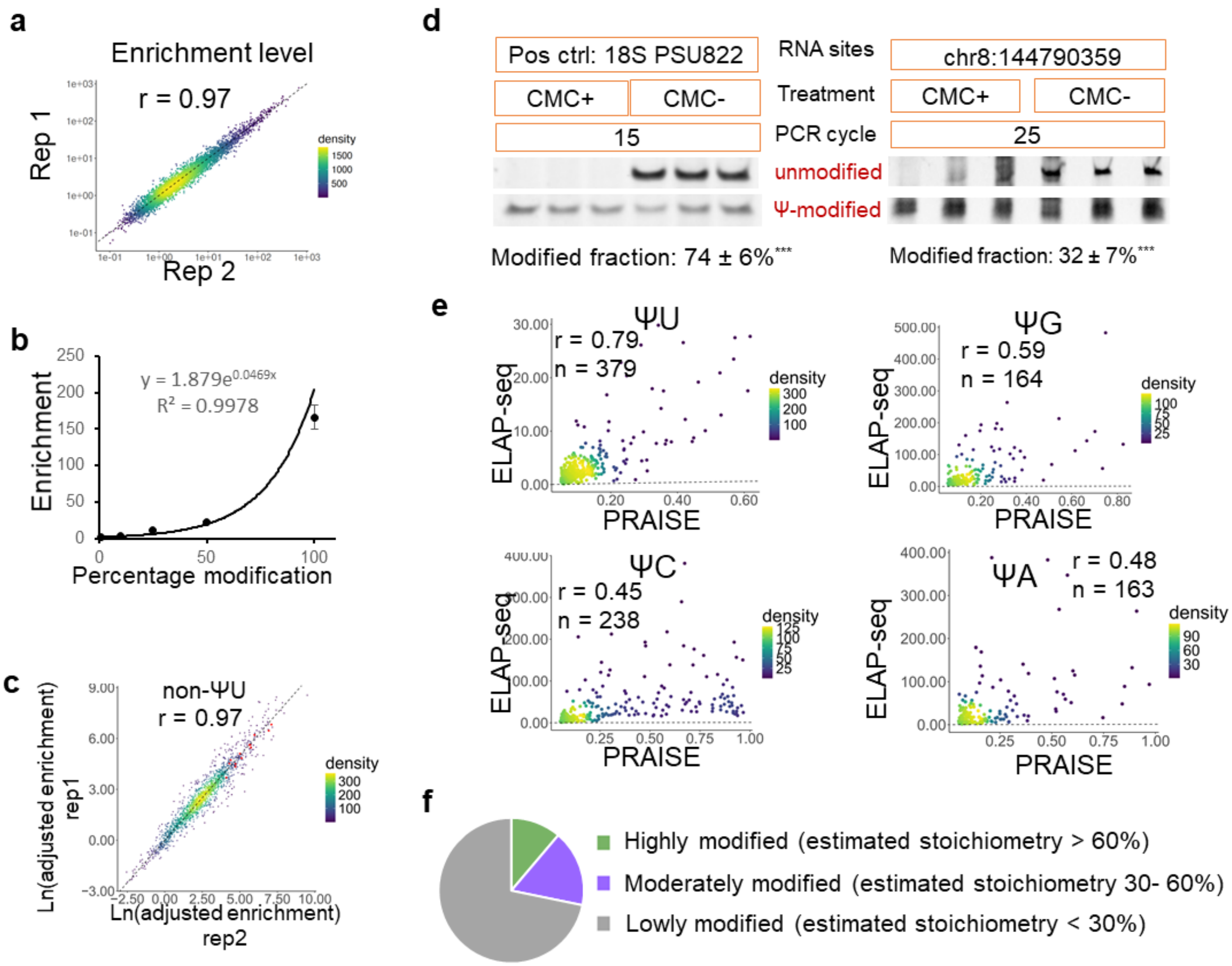

Supplementary Fig 15. Semi-quantitative evaluation of Ψ modification levels. a) Correlation of enrichment values between two biological replicates for all candidate sites. b) Pulling down synthetic RNAs modified to various levels reveals an exponential correlation between the modification level and the pull-down enrichment level. c) Adjusted enrichment level which indicates relative modification extent for sites in the non-ΨU contexts. Red dots denote known modification sites on rRNA d) Quantification of modification stoichiometry for one candidate site using CLAP. A modification site on rRNA is used as the positive control. e) Correlation of adjusted enrichment levels in ELAP-seq and deletion ratios in PRAISE for Ψ sites in various sequence contexts. f) Pie chart depicting the categorization of candidate Ψ sites detected by ELAP-seq in HEK 293T poly-A RNA into different modification levels.

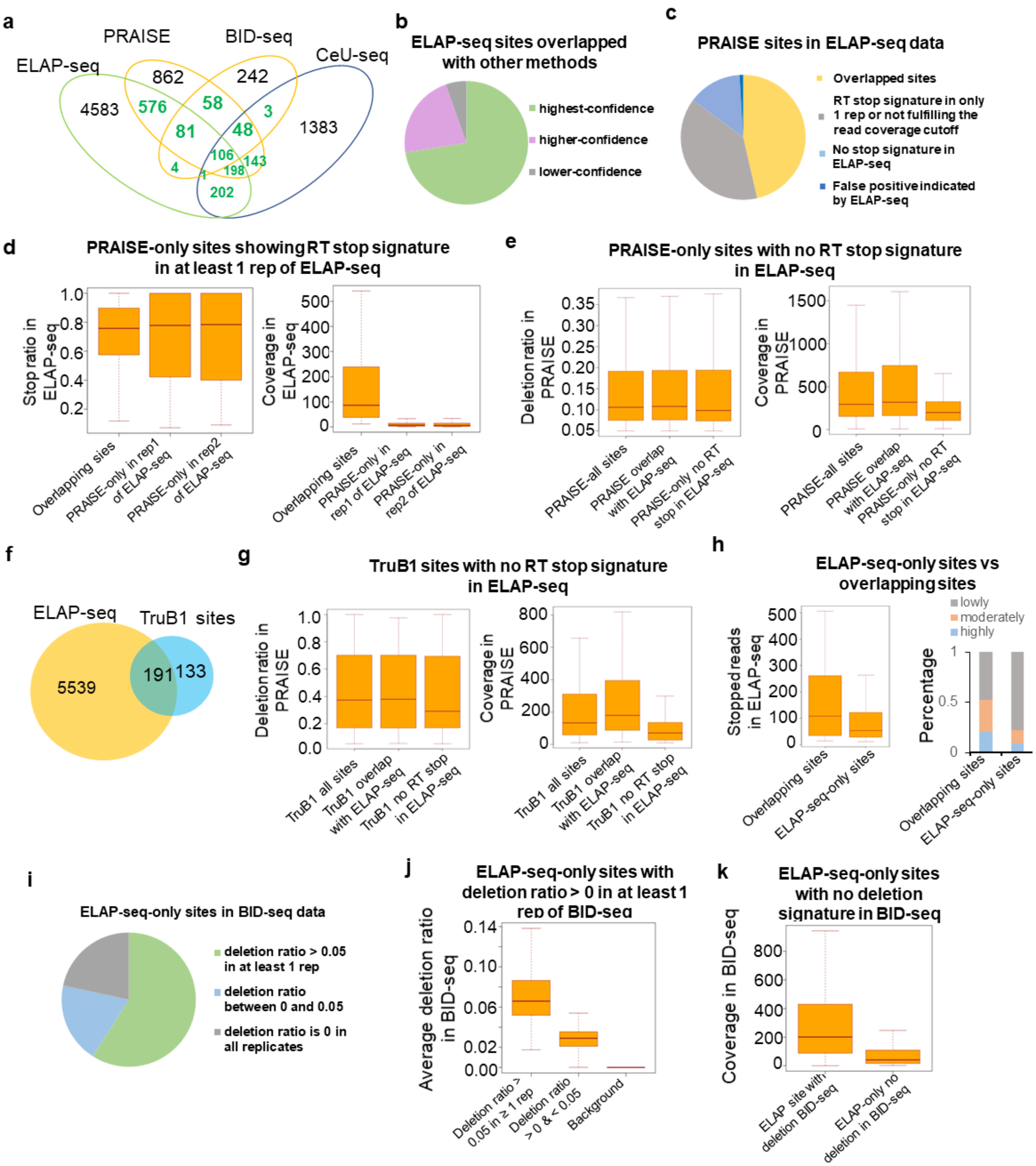

Supplementary Fig 16. Comparison of ELAP-seq to other methods. a) Venn diagram depicting the overlap among Ψ sites detected by ELAP-seq, PRAISE, BID-seq and CeU-seq for HEK 293T cells. b) The distribution of ELAP-seq Ψ sites overlapping with other methods in the three confidence categories. c) Overview of signals of PRAISE sites in the ELAP-seq dataset. The false positives were determined based on that those sites were covered by multi-mapped reads, potentially leading to repeated counts, or identical stop signatures in both the pull-down and input in our dataset. d) Signals in ELAP-seq dataset for PRIASE-only sites (sites not overlapping with the ELAP-seq Ψ list) showing prominent stop signatures in at least one replicate in ELAP-seq. e) Signals in PRAISE dataset for PRAISE-only sites showing no RT stop signals in any

replicate of ELAP-seq. These sites showed overall lower deletion ratios and read coverage than the rest sites. f) Overlap between ELAP-seq Ψ list and the TruB1-dependent sites reported by PRAISE. g) Signals in the PRAISE dataset for TruB1-dependent sites not overlapping with the ELAP-seq Ψ list. They have overall lower deletion ratios and read coverage compared to the rest sites. h) Signals in the ELAP-seq dataset for ELAP-seq-only sites and ELAP-seq sites overlapping with other methods. ELAP-seq-only sites have overall lower reads coverage and contain a higher fraction of lowly modified sites than the overlapping sites. i) Overview of signals of ELAP-seq-only highest- and higher-confidence sites in the BID-seq dataset. j) Average deletion ratio signals in BID-seq dataset for ELAP-seq-only sites with deletion signals higher than 0 in at least one replicate of BID-seq dataset. Background is defined by randomly chosen U sites not reported as Ψ by any method. k) Signals in the BID-seq dataset for ELAP-seq-only sites with deletion ratio of 0 in all replicates of BID-seq dataset. They showed substantially lower read coverage compared to the rest sites.

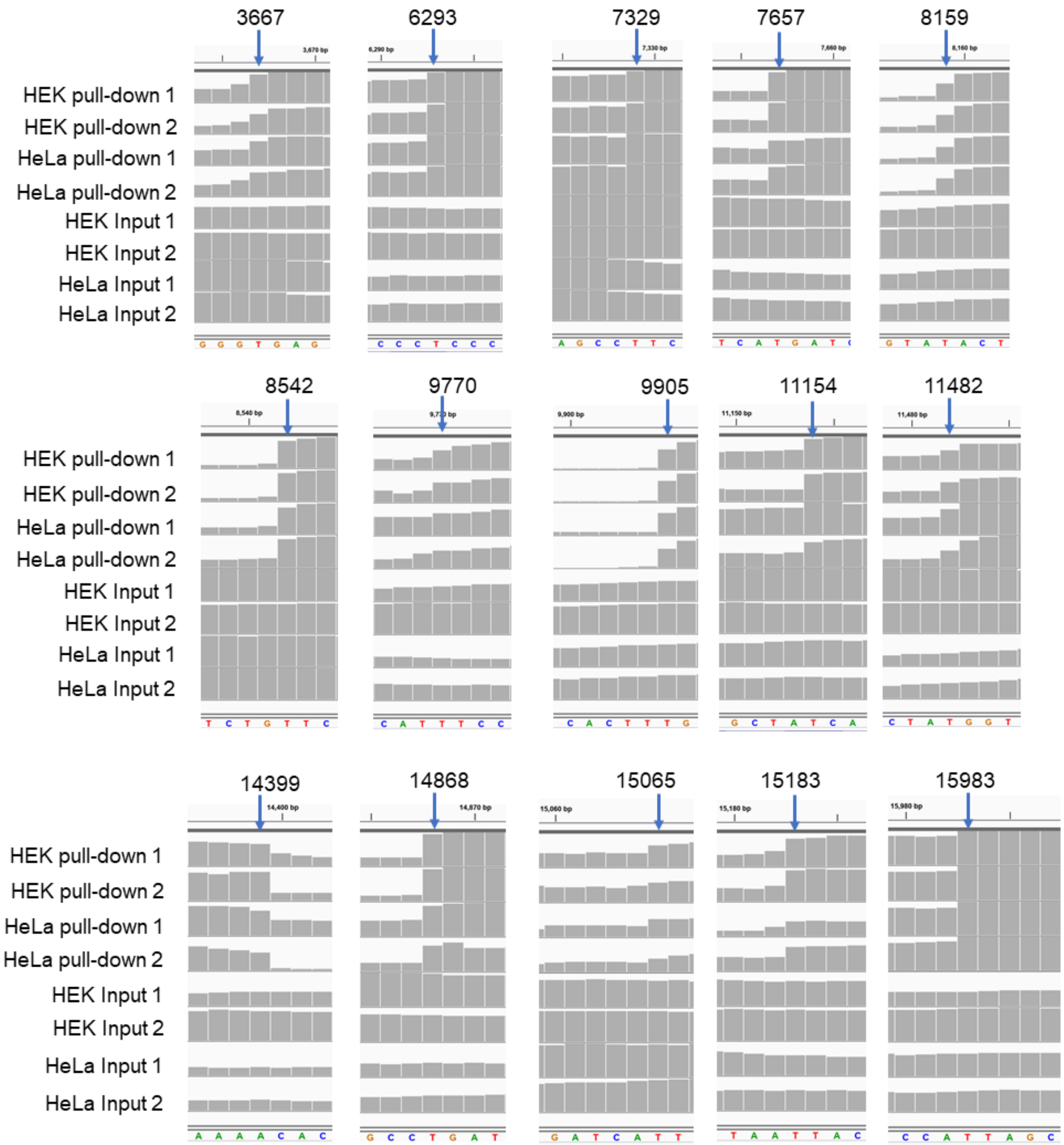

Supplementary Fig 17. 15 mitochondrial mRNA modification sites shared between HeLa and HEK 293T cell lines.

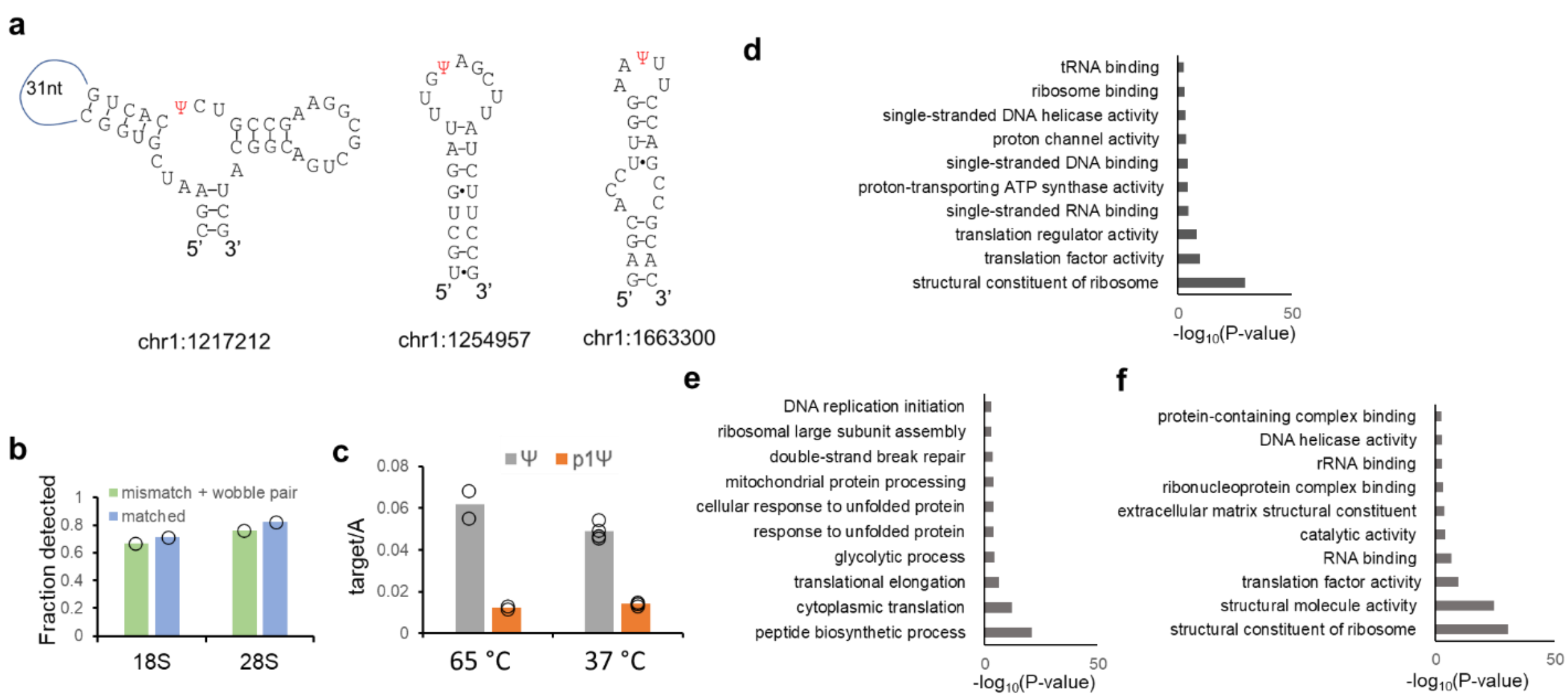

Supplementary Fig 18. Ψ detected by ELAP-seq provides new insights into the biogenesis and functions of Ψ. a) RNA-fold analysis shows examples of Ψ modification locating in the less structured region of mRNA. b) Sites in matched positions in rRNA were as effectively detected as sites in mismatched and wobble positions. n=1. c) The enzymatic labeling efficiency on isolated total RNA at 37 °C is the same as that under the temperature of 65 °C. Data at 65 °C: n=2. Data at 37 °C: n=4. d) Top 10 enriched GO clusters of molecular functions for Ψ-modified genes in HEK 293T cells. e) Top 10 enriched GO clusters of biological process for Ψ-modified genes in HeLa cells. f) Top 10 enriched GO clusters of molecular function for Ψ-modified genes in HeLa cells.
