# Supplementary material for: Enzyme-mediated alkynylation enables transcriptome-wide identification of pseudouridine modifications
Source: Nat Commun. 2026 Mar 23;17:4318. doi: 10.1038/s41467-026-70597-8 (PMC13171889; doi:10.1038/s41467-026-70597-8)
Supplement: Supplementary file 2 [file 41467_2026_70597_MOESM2_ESM.pdf]

Description of Additional Supplementary Files

File Name: Supplementary Data 1
Description: Candidate Ψ identified by ELAP-seq in HEK 293T RNA/

File Name: Supplementary Data 2
Description: Candidate Ψ identified by ELAP-seq in HeLa RNA.

File Name: Supplementary Data 3
Description: Candidate Ψ identified by ELAP-seq in HeLa mitochondrial RNA.

File Name: Supplementary Data 4
Description: Candidate Ψ identified by ELAP-seq in HEK 293T mitochondrial RNA.

File Name: Supplementary Data 5
Description: rRNA modification sites identified by ELAP-seq.

File Name: Supplementary Data 6
Description: Changes in enrichment levels at Ψ modification sites in response to DKC1 knockdown.

File Name: Supplementary Data 7
Description: Primers used for CMC-RT-qPCR validation.

File Name: Supplementary Data 8
Description: Internally barcoded RNA adapters used for building DKC1 control and knockdown libraries.
