# Supplementary material for: Enzyme-mediated alkynylation enables transcriptome-wide identification of pseudouridine modifications
Source: Nat Commun. 2026 Mar 23;17:4318. doi: 10.1038/s41467-026-70597-8 (PMC13171889; doi:10.1038/s41467-026-70597-8)
Supplement: Supplementary file 3 [file 41467_2026_70597_MOESM4_ESM.pdf]

nature portfolio

Corresponding author(s): Yuru Wang

Last updated by author(s): Feb 17, 2026

Reporting Summary

Nature Portfolio wishes to improve the reproducibility of the work that we publish. This form provides structure for consistency and transparency in reporting. For further information on Nature Portfolio policies, see our Editorial Policies and the Editorial Policy Checklist.

Statistics

For all statistical analyses, confirm that the following items are present in the figure legend, table legend, main text, or Methods section.

n/a	Confirmed	
☐	☒	The exact sample size (*n*) for each experimental group/condition, given as a discrete number and unit of measurement
☐	☒	A statement on whether measurements were taken from distinct samples or whether the same sample was measured repeatedly
☐	☒	The statistical test(s) used AND whether they are one- or two-sided<br>*Only common tests should be described solely by name; describe more complex techniques in the Methods section.*
☒	☐	A description of all covariates tested
☐	☒	A description of any assumptions or corrections, such as tests of normality and adjustment for multiple comparisons
☐	☒	A full description of the statistical parameters including central tendency (e.g. means) or other basic estimates (e.g. regression coefficient) AND variation (e.g. standard deviation) or associated estimates of uncertainty (e.g. confidence intervals)
☐	☒	For null hypothesis testing, the test statistic (e.g. *F*, *t*, *r*) with confidence intervals, effect sizes, degrees of freedom and *P* value noted<br>*Give P values as exact values whenever suitable.*
☒	☐	For Bayesian analysis, information on the choice of priors and Markov chain Monte Carlo settings
☒	☐	For hierarchical and complex designs, identification of the appropriate level for tests and full reporting of outcomes
☐	☒	Estimates of effect sizes (e.g. Cohen's *d*, Pearson's *r*), indicating how they were calculated

Our web collection on statistics for biologists contains articles on many of the points above.

Software and code

Policy information about availability of computer code

Data collection	Illumina sequencing system; Bruker Ultraflextreme MALDI-TOF-TOF
Data analysis	Link to Github: https://github.com/yuruwang26/ELAP-seq-v4 DOI: 10.5281/zenodo.18673787. To analyze MALDI data: flexanalysis 3.4.

For manuscripts utilizing custom algorithms or software that are central to the research but not yet described in published literature, software must be made available to editors and reviewers. We strongly encourage code deposition in a community repository (e.g. GitHub). See the Nature Portfolio guidelines for submitting code & software for further information.

Data

Policy information about availability of data

All manuscripts must include a data availability statement. This statement should provide the following information, where applicable:

- Accession codes, unique identifiers, or web links for publicly available datasets
- A description of any restrictions on data availability
- For clinical datasets or third party data, please ensure that the statement adheres to our policy

The NGS data generated in this paper has been deposited in the GEO database under accession code GSE236530.

Research involving human participants, their data, or biological material

Policy information about studies with human participants or human data. See also policy information about sex, gender (identity/presentation), and sexual orientation and race, ethnicity and racism.

Reporting on sex and gender	No human participants were involved
Reporting on race, ethnicity, or other socially relevant groupings	No human participants were involved
Population characteristics	No human participants were involved
Recruitment	No human participants were involved
Ethics oversight	No human participants were involved

Note that full information on the approval of the study protocol must also be provided in the manuscript.

Field-specific reporting

Please select the one below that is the best fit for your research. If you are not sure, read the appropriate sections before making your selection.

[x] Life sciences [ ] Behavioural & social sciences [ ] Ecological, evolutionary & environmental sciences

For a reference copy of the document with all sections, see nature.com/documents/nr-reporting-summary-flat.pdf

Life sciences study design

All studies must disclose on these points even when the disclosure is negative.

Sample size	No statistical methods were used to predetermine sample size. Sample size were determined based on our prior experience on similar experiments and literature reports. All sequencing for HeLa and HEK293T cell lines were performed in triplicates. Sequencing for siCtrl and siDKC1 cells were performed in duplicates.
Data exclusions	No data was excluded
Replication	Results were confirmed in at least two biological replicates for each experiment as stated in the figure legends. All attempts to replicate data are successful.
Randomization	The experiments were not randomized. Controlling for covariates was unnecessary because all assays were performed in pairs.
Blinding	Blinding was not applicable because the focus of this paper did not involve group allocation and blinding.

Reporting for specific materials, systems and methods

We require information from authors about some types of materials, experimental systems and methods used in many studies. Here, indicate whether each material, system or method listed is relevant to your study. If you are not sure if a list item applies to your research, read the appropriate section before selecting a response.

Materials & experimental systems

n/a	Involved in the study	
[ ]	[x]	Antibodies
[ ]	[x]	Eukaryotic cell lines
[x]	[ ]	Palaeontology and archaeology
[x]	[ ]	Animals and other organisms
[x]	[ ]	Clinical data
[x]	[ ]	Dual use research of concern
[x]	[ ]	Plants

Methods

n/a	Involved in the study	
[x]	[ ]	ChIP-seq
[x]	[ ]	Flow cytometry
[x]	[ ]	MRI-based neuroimaging

Antibodies

Antibodies used	rabbit anti-DKC1 (proteintech Cat# 25420-1-AP), rabbit anti-actin (proteintech cat# 20536-1-AP), mouse anti-pseudouridine (proteintech Cat# 68578-1-Ig) and mouse anti-pseudouridine (MBL life science Code No. D347-3)

Validation | These antibodies have been validated in our hand for western blot.

Eukaryotic cell lines

Policy information about cell lines and Sex and Gender in Research

Cell line source(s)	HEK 293T cells were purchased from ATCC (cat# CRL-3216); HeLa cells were purchased from ATCC (cat# CCL-2)
Authentication	Cell lines were not authenticated after purchase from ATCC.
Mycoplasma contamination	All cell lines were tested negative for mycoplasma contamination with MycoProbe Mycoplasma Detection kit
Commonly misidentified lines (See ICLAC register)	Not relevant

Plants

Seed stocks	Not relevant
Novel plant genotypes	Not relevant
Authentication	Not relevant
