# Supplementary material for: Enzyme-mediated alkynylation enables transcriptome-wide identification of pseudouridine modifications
Source: Nat Commun. 2026 Mar 23;17:4318. doi: 10.1038/s41467-026-70597-8 (PMC13171889; doi:10.1038/s41467-026-70597-8)
Supplement: Supplementary file 4 [file 41467_2026_70597_MOESM5_ESM.pdf]

nature portfolio

Peer Review File

Enzyme-mediated alkynylation enables transcriptome-wide identification of pseudouridine modifications

Corresponding Author: Dr Yuru Wang

This file contains all reviewer reports in order by version, followed by all author rebuttals in order by version.

Version 0:

Reviewer comments:

Reviewer #1

(Remarks to the Author)
Pseudouridine (Ψ) is an abundant and critical RNA modification, yet the full extent of its biological roles in the cell have been difficult to understand due to incomplete transcriptome-wide maps of modification sites. One of the major bottlenecks in generating comprehensive maps with current mapping methods is the extensive sequence depth required to confidently assign modifications globally, due the sparse distribution and general low occupancy of Ψ. In this manuscript, Wang et al., present an innovative method to enrich for RNA fragments containing Ψ by using an archaeal methyltransferase, which they claim can specifically add click-chemistry compatible propargyl groups to Ψ to facilitate biotin conjugation. This addition allows them to both enrich for modified RNA using streptavidin, and obtain single nucleotide resolution of modification position using RT-stop signatures induced by the biotin adduct. They apply this method to correctly identify most known rRNA pseudouridine sites and generate a map of pseudouridylation in human mRNA across three cell types and make several claims about global Ψ distribution.
While we find this technique to be a potentially powerful new tool for profiling Ψ, the lack of overlap with existing methods and little validation for the many new sites uncovered is cause for concern. Additionally, several of the claims made about Ψ occupancy and distribution need more supporting evidence or explanation. We do not recommend acceptance of this manuscript until the following points are addressed:

Major Concerns:

1. According to figure S11, only 4% of sites identified in HeLa cells overlap with previous mapping datasets, and 8% overlap in 293T. The authors do not address this discrepancy beyond this figure, except to suggest it is due to poor performance of CMC-based methods. More evidence is needed to demonstrate that the mRNA data is capturing mostly genuine Ψ modifications, including performing PUS enzyme depletions to demonstrate PUS-dependence of signal, and far more targeted validations (ex. using CLAP), specifically of sites not recapitulated by other datasets.

2. The authors claim that 1/3 of sites have over a 50% modification rate, much higher than the average mRNA modification rates estimated from other methods such as BID-Seq, and inconsistent with their claim that the massive number of new sites are from enrichment of lowly-modified positions. The authors must more rigorously demonstrate that their estimation of modification occupancy is accurate, by correlating their estimates with estimates from mutational-profiling methods such as BID-Seq, and by demonstrating correlation with many more than 2 sites estimated by CLAP. Also, the CLAP vs ELAP-Seq comparison is difficult to see in figure 4h and should have its own figure panel (with the additional comparisons).

3. The language explaining the rational for cutoffs and filtering is vague. Please provide specific details with statistical justification for the following:

“We optimized cutoffs for stop ratio and reads coverage and allow for gradients of coverage for different ranges of stop ratios” [275-276]

“We required higher reads coverage for lower levels of stop ratios to decrease false positives” [685-686]

“We allowed slightly lower reads coverage for sites in the out-group” [688-689]

“…we screened cutoffs for stop ratios and coverage to find a balance between the number of sites identified and percentage of U” [689-691]

4. For the IFN dataset it is mentioned that “the interferon stimulated samples (IFN+) resulted in ~40% less reads compared to untreated samples (IFN-), thus we allowed lower reads coverage cutoff for the IFN+ samples.”[715-719] How does this not increase the false positive rate for the INF+ samples – if the read cutoffs were set to minimize false positive rate? Intuitively this cutoff should not be determined by read depth. This comparison would be more accurate with the INF- samples randomly sub-sampled down to the same depth as INF+, using identical read cutoffs.

Minor critiques:

“a significant proportion of Ψ sites in mRNA appear in the 5’-ΨU-3’ context…” [203-204] what proportion exactly?

“30 – 60% of Ψ in cellular mRNA can be labeled by this enzyme” [205-206] Do you expect 30-60% of Ψ positions are being labeled – or each site is being labeled at a 30-60% rate?

Why is there a stop in untreated RNA at the expected psi position in figure 2e?

How do the NNpsiNN pools perform in the standard library prep? E.g. are there any RT-stop or ligation biases due to sequence context?

It is not described how the wobble pair analysis was performed, please add more detail in the methods or main text.

Enrichment of Ψ at splice junctions is only informative with a comparison against the background distribution of U. Plot both distributions from your dataset and demonstrate that the difference in Ψ distribution is significant.

Reviewer #3

(Remarks to the Author)
The manuscript by Wang et al developed ELAP-seq to enrich and map Ψ at single-base resolution. This new Ψ sequencing technology leveraged enzymatic strategy to label and enrich Ψ-modified transcripts. They have identified approximately 10, 000 candidate Ψ sites in HEK293T and HeLa cell lines, a significantly larger number compared to all previous CMC-based and bisulfite-based methods. Based on this approach, the authors found differential Ψ modification upon type-I interferon stimulation in the glioblastoma cell line. Overall, the manuscript provides a new sequencing method and applies it to detect Ψ modification in the human transcriptome. Unfortunately, the major concerns remain non-specificity, unclear false positives, and unclear false negatives. Some weaknesses need to be addressed before this manuscript would be suitable for publication:

Major:
1. The authors reported around ten thousand candidate Ψ sites from HeLa and HEK 293T cell lines. However, more than 9000 sites currently lack validation through existing datasets. Before asserting their claims of high sensitivity, the authors have to verify the specificity of ELAP-seq instead of just providing the ambiguous Ψ lists. The authors should perform perturbation of PUS proteins, such as employing PUS knockdowns or knockouts in these cells, to assign the majority of the newly identified Ψ sites to specific enzymes. This assignment would validate the specificity of ELAP-seq and contribute to functional insights.

2. A more comprehensive comparison should be performed between the Ψ dataset sourced from HEK293T and HeLa cells in ELAP-seq and their previously published human mRNA pseudouridine maps (Dai, Q. et al. Nat Biotechnol. 2023). They should analyze the raw deletion signal for ELAP-seq-unique Ψ sites in BID-seq classify the authenticity of these sites and provide a clear explanation for their lack of detection in BID-seq.

3. In L187, the strong bias for ΨU context might even underestimate the GUΨC context modified by TRUB1 protein which has the highest modification activity, which indicates the severe false negative of ELAP-seq. The authors need to demonstrate they could detect all these TRUB1-dependent sites from previous Ψ datasets.

4. The authors claim the high sensitivity and low background of ELAP-seq; however, they have yet to provide substantial supporting evidence for this assertion. (1) For Ψ in rRNA, they only identified 70 out of 104 known Ψ sites in rRNA (L250), since Mj1640 the strong preference for 5’-ΨU-3’ sequence context in oligo RNA, they should analyze these 34 missed known Ψ sites, and then address whether these omissions were a result of the sequence context instead of being clustered within specific rRNA regions. (2) For Ψ in mRNA, although the author can filter out these highly modified Nm sites through bioinformatic cutoff, they cannot exclude the existence of false-positive Ψ sites derived from Nm modifications that vary with differential modification levels in mRNA, hence, the reliability of ten thousand of Ψ is questionable. The comparison and overlap analysis of Ψ sites identified via ELAP-seq with Nm sites identified through Nm-seq should be performed (Dai, Q., et al Nat Methods, 2017).

5. In L267 and Figure 4a, how does the mitochondrial mRNA Ψ result compare to the previous Ψ modification map obtained

by BID-seq? The comparison of mitochondrial Ψ sites should be performed for both HEK293T and HeLa cell lines.

6. In L328, the authors claim “excellent” repeatability for Ψ detection in mRNA. They should provide mRNA Ψ site numbers for three biological replicates and hence the ratio of overlapping sites.

7. In L432, the authors need to provide more experimental and bioinformatic analysis for 92 downregulated Ψ sites upon interferon-α treatment, for instance, they can analyze the motif and investigate whether they were modified by PUS3 protein. The authors could also detect the protein level of relevant PUS enzymes between IFN- and IFN+ samples to explain why Ψ was downregulated.

Minor:
1. In L55, the statement regarding quantitative mapping methods is incorrect, RBS-seq is not a quantitative approach.

2. In L130, data was not shown for the control U-RNA.

3. In L146, the authors should provide experimental data to demonstrate the capacity of the Mj1640 enzyme to label the synthetic 8-mer oligo using propargyl-SeAm.

4. In L158, the size distribution of untreated mRNA should be provided.

Version 2:

Reviewer comments:

Reviewer #3

(Remarks to the Author)
During revision, the authors have incorporated a substantial number of bioinformatics analyses. These analyses encompass overlap studies with the PRAISE, BID-seq, and CeU-seq methodologies. Additionally, the authors have performed DKC1 knockdown experiments to identified PUS-dependent sites.The potential false positives and false negatives associated with the technology have been thoroughly discussed in the discussion section. However, two minor concerns remain to be addressed:
1. The authors should provide data from NNUNN spike-in experiments to further validate the specificity of the technology.
2. In Figure S8b, despite the authors having performed three replicates, the reproducibility of the modification sites is only approximately 20%. A detailed explanation and clarification of this discrepancy are required.

(Remarks on code availability)

Reviewer #4

(Remarks to the Author)
In this manuscript, Wang et al. present a new transcriptome-wide pseudouridine (Ψ) site profiling method (ELAP-seq) using the Mj1640 enzyme to modify Ψ sites with a propargyl group and click-chemistry for biotin labeling. The authors achieved enrichment of Ψ by streptavidin pulldown and single nucleotide resolution by harnessing the RT-stop signature. For the most part, the authors have addressed concerns voiced by Reviewer 1 and Reviewer 3. The most appealing finding in this current manuscript is the detection of many more Ψ sites compared to previous methods, with some sites further validated by orthogonal methods. However, the potential strength of this enzyme-based method appears to be underdeveloped, and the inconsistency of cutoffs and filtering makes it difficult to follow. These concerns, combined with the relative simplicity, quantitativeness, and cost-effectiveness without need for commercially unavailable enzyme of other methods (particularly bisulfite-Ψ adduct methods PRAISE for transcriptome-wide Ψ profiling and BID-Seq for targeted Ψ profiling) severely limit the utility of ELAP-seq to the Ψ research community.

As such, the current version of this manuscript is not well suited for publication in this journal, in our opinion. The manuscript would be more useful as either a resource of novel Ψ sites (which would require a lot more validation, particularly for lower confidence sites) or a revelation of novel biology that existing methods cannot capture.

Major comments:
1. The authors claim that ELAP-seq could be used in live cells for Ψ profiling, but in the ELAP-Seq protocol they denature fragmented isolated RNA prior to enzymatic labeling. This is a contradiction, because RNA secondary structure is influenced by conditions unique to cells not present in denatured fragmented isolated RNA, including long-range RNA-RNA interactions and RNA-binding proteins. For this reason, it is not obvious to the reader that in situ ELAP-Seq would work. Certainly in vivo ELAP-Seq, as hinted at in Supplementary Fig. S3, would not work.
2. In the Introduction, the authors emphasize that many other methods for profiling Ψ sites require a high level of RNA input and/or deep sequencing. Although the authors showed the detection sensitivity of this assay on synthesized oligos in Figure 2b, the detection limit of this assay on real mRNA samples from cells is unclear (based on the Methods section, ELAP-seq

appears to be carried out with 200-1000ng of RNA, but it is unclear if it is total RNA or isolated mRNA). In this aspect, it remains uncertain whether ELAP-seq can be used for rare cell populations that other methods could hardly approach.
3. In lines 234-236 the authors decided to use data generated with Superscript III for detecting Ψ sites, while in lines 287-289 the authors decided to combine Superscript III and Superscript IV data to yield better signal. The inconsistency of data analysis makes it difficult to follow. It might be better to fix a filtering and cutoff strategy with rRNA benchmarking and then stick to the same filtering so that the level of false positives can be more easily estimated.
4. In lines 278-280 and a few cases later in the manuscript, as well as in Fig. 4c-e, the authors detected Ψ sites at U, G, A, C with different proportions, while when describing their filter strategy (lines 246-247) they only include sites for which "the stop event occurs at uridine (U)." It is unclear why these G, A, C sites were still included with their pipeline, unless the authors really mean ΨU, ΨG, ΨA, and ΨC.
5. In the paragraphs to study Ψ sites in response to IFN treatment, the phenotype was not well explained. By LS-MS/MS, there seemed to be a 20-25% reduction of global Ψ, while by ELAP-seq there seemed to be 41-52% fewer sites. The authors should explain this discrepancy. Additionally, since the protein level of PUS enzymes did not change, it is unclear how robust this assay was. The authors should either demonstrate Ψ change with a different method or identify other enzymes/regulators to explain the phenotype.
6. Pull-down of both enzymatic and control reactions in ELAP-seq means that the methodology will likely be even less quantitative than other semi-quantitative methods for lower RNA inputs. It is hard to imagine that spike-ins mentioned in lines 564-565 would alleviate this concern.

Minor comments:
1. In the rebuttal, the authors write that, "Performed knockdown experiment for one of the pseudouridine writers, DKC1, which led to the identification of 1014 sites confidently downregulated due to DKC1 depletion, including 882 sites newly identified by ELAP-seq (Figure 4h and the new supplementary Figure S10)." The authors should analyze how much of this change is due to transcript expression changes leading to lower confidence in calling Ψ sites vs. bona fide Ψ downregulation.
2. In line 114, the authors write, "data not shown." They should show the data or remove/restate the sentence.
3. In line 232-234, the authors do not show Superscript IV data.
4. The authors should state the precise statistical test used to generate the p-value stated in line 241.
5. In line 466, Fig. 5f should be Fig. 5e.
6. In the Discussion in lines 604-605, the authors speculate that Mj1640 could be used to selectively introduce m1Ψ to mRNA vaccines. It is hard to imagine that such a strategy would be productive, given that such a change would inherently increase immunogenicity and reduce protein translational efficiency.

(Remarks on code availability)

Version 3:

Reviewer comments:

Reviewer #3

(Remarks to the Author)
The authors have addressed the two minor comments raised during the revision. Regarding the first point, while the inclusion of the spike-in results in Figure S5 is appreciated, the color scheme requires adjustment as it is currently visually harsh. Additionally, the figure legend needs to be expanded to provide more detailed descriptive information.

(Remarks on code availability)
The provided code is user-friendly and the documentation is sufficient. I was able to replicate the installation and execution processes seamlessly.

Reviewer #4

(Remarks to the Author)
In their updated manuscript, Wang et al. substantially improve their report on the enzyme-based pseudouridine detection method ELAP-Seq. Beyond demonstrating enhanced sensitivity of the method, they have removed incomplete data and unsupported claims. With a few minor changes, their work could be suitablel for publication in Nature Communications.

1. The authors should add accessibility information on the Addgene-deposited Mj1640 expression plasmid to the Materials and Methods.
2. The authors should remove references to the interferon treatment experiment from the Materials and Methods.
3. I am not convinced by the authors' rebuttal to my previous minor comment 1.
a. For reference, I originally wrote: "In the rebuttal, the authors write that, "Performed knockdown experiment for one of the pseudouridine writers, DKC1, which led to the identification of 1014 sites confidently downregulated due to DKC1 depletion, including 882 sites newly identified by ELAP-seq (Figure 4h and the new supplementary Figure S10)." The authors should analyze how much of this change is due to transcript expression changes leading to lower confidence in calling Ψ sites vs. bona fide Ψ downregulation."
b. The authors respond: "Our method to measure differential RNA modification uses changes in the enrichment level,

defined as the RPM in the pull-down library normalized by that in the input library (please see main text at page 20, lines 380-381 and Material and Method section at page 42, lines 874-876), which already takes into account the change in the transcriptional expression level. Therefore, these are bona fide downregulation, instead of false positives due to a decrease in the gene expression."
c. The specific problem I have with this response is what the authors write in the previous paragraph of their rebuttal: "We also note that reads coverage can influence quantification accuracy: mRNA sites in the ΨU context, rRNA sites and mRNA sites in the non-ΨU context readily detectable by CLAP, which generally have high read coverage, correlate better with PRAISE quantification than mRNA sites in non-ΨU context, which generally have low read coverage (Figure 4i and S15e)."
d. There appears to be a clear contradiction—if lower read coverage results in lower quantification accuracy, presumably it also results in lower confidence of called Ψ sites. If this is true, then decreased gene expression can indeed lead to an increase in sites confidently downregulated. As is common in RNA enrichment method benchmarking, the authors should perform additional analysis of their RNA-seq data (Fig. S12a). Specifically, they should assess the degree to which genes harboring 717 (previously 1014—I am not sure what caused the change) Ψ sites confidently downregulated are themselves downregulated in gene expression. I propose that the authors first generate a differential gene expression analysis volcano plot of all RNA-Seq genes with the genes harboring these 717 Ψ sites plotted in a different color. Additionally, they should quantify the distribution in fold-change of the Ψ site-harboring genes relative to the fold-change of all genes. Such analysis will suitably resolve this open question.

(Remarks on code availability)

Open Access This Peer Review File is licensed under a Creative Commons Attribution 4.0 International License, which permits use, sharing, adaptation, distribution and reproduction in any medium or format, as long as you give appropriate credit to the original author(s) and the source, provide a link to the Creative Commons license, and indicate if changes were made.
In cases where reviewers are anonymous, credit should be given to 'Anonymous Referee' and the source.
The images or other third party material in this Peer Review File are included in the article's Creative Commons license, unless indicated otherwise in a credit line to the material. If material is not included in the article's Creative Commons license and your intended use is not permitted by statutory regulation or exceeds the permitted use, you will need to obtain permission directly from the copyright holder.
To view a copy of this license, visit https://creativecommons.org/licenses/by/4.0/

We thank the reviewers for the detailed analysis and constructive comments of our manuscript. Below, we have positively addressed reviewers' concerns regarding the authenticity, the reproducibility, and the quantitative capability of the ELAP-seq method by performing additional experiments, conducting thorough data analysis, as well as re-writing part of the manuscript. We have also improved the data analysis pipeline and provided a one-step command. We appreciate the suggestions from the reviewers to perform additional work which greatly enhanced the clarity and usability of our method.

REVIEWER COMMENTS

REVIEWER COMMENTS

Reviewer #1 (Remarks to the Author):

Pseudouridine (Ψ) is an abundant and critical RNA modification, yet the full extent of its biological roles in the cell have been difficult to understand due to incomplete transcriptome-wide maps of modification sites. One of the major bottlenecks in generating comprehensive maps with current mapping methods is the extensive sequence depth required to confidently assign modifications globally, due the sparse distribution and general low occupancy of Ψ. In this manuscript, Wang et al., present an innovative method to enrich for RNA fragments containing Ψ by using an archaeal methyltransferase, which they claim can specifically add click-chemistry compatible propargyl groups to Ψ to facilitate biotin conjugation. This addition allows them to both enrich for modified RNA using streptavidin, and obtain single nucleotide resolution of modification position using RT-stop signatures induced by the biotin adduct. They apply this method to correctly identify most known rRNA pseudouridine sites and generate a map of pseudouridylation in human mRNA across three cell types and make several claims about global Ψ distribution.
While we find this technique to be a potentially powerful new tool for profiling Ψ, the lack of overlap with existing methods and little validation for the many new sites uncovered is cause for concern. Additionally, several of the claims made about Ψ occupancy and distribution need more supporting evidence or explanation. We do not recommend acceptance of this manuscript until the following points are addressed:

Major Concerns:

1. According to figure S11, only 4% of sites identified in HeLa cells overlap with previous mapping datasets, and 8% overlap in 293T. The authors do not address this discrepancy beyond this figure, except to suggest it is due to poor performance of CMC-based methods.

More evidence is needed to demonstrate that the mRNA data is capturing mostly genuine Ψ modifications, including performing PUS enzyme depletions to demonstrate PUS-dependence of signal, and far more targeted validations (ex. using CLAP), specifically of sites not recapitulated by other datasets.

Our response:

We are grateful for the constructive feedback from reviewer #1. The reviewer has noted a 4% and 8% overlap between our data and previously reported sites for HeLa and 293T mRNA, respectively. The lower percentage of overlap is attributed to a greater number of sites identified by our method due to its higher sensitivity, which we view as an advantageous feature.

In this revised version of manuscript, we performed a comparative analysis with the three most recently published bisulfite-based methods (BID-seq, RBS-seq, and PRAISE). Our method demonstrated substantial coverage, and successfully identified 42%, 49%, and 60% of the reported sites, respectively (Figure S10a). Overall, our data encompassed 1,904 of the 4,333 previously reported sites for 293T mRNA (combined data from BID-seq, PRAISE and CeU-seq) (Figure S10a). Our dataset not only validated these modification sites but also facilitated the precise localization of Ψ modifications within multiple Us, a challenging task for the current bisulfite-based approaches.

To address discrepancies with previous methods, we further looked at signals of PRAISE-unique sites in our dataset. Our data uncovered 1,237 of the 2,070 sites reported by PRAISE (Figure S10 a-b). Among the 833 PRAISE-unique sites, 526 exhibited prominent stop signatures in at least one replicate of our data, with 113 showing signals in two replicates. However, these sites were excluded from our final list due to being present in only one replicate or having low read coverage (Figure S10e). Only 288 sites (13.9%) lacked any signal in our dataset, and these sites were found to have lower deletion ratios and read coverage than the rest Ψ sites in the PRAISE-seq dataset (Figure S10f), suggesting that they are intrinsically more difficult to identify and may have lower confidence. Our examination also identified 11 sites determined by PRAISE that were covered by multi-mapped reads, potentially leading to repeated counts, and 8 sites flagged as false positives due to identical stop signatures in both the pull-down and input in our data.

Similarly, for the 302 BID-unique sites, we identified 65 additional sites showing prominent stop signals in at least one replicate of our dataset, with those lacking signals in our dataset mainly situated in unfavorable sequence contexts and displaying low deletion levels in BID-seq dataset.

Furthermore, our data covered 70% of TruB1 sites reported by PRAISE (Figure S10h) and all mitochondria mRNA sites reported by PRAISE and BID-seq (Supplementary table 3-4).

Notably, by comparing the U sites and non-U sites identified by our method, we found that increasing cutoff for stop ratio could increase the degree of confidence for the reported sites (Figure 4 g-h). Additionally, by performing a third replicate for each cell line, we found that the

degree of confidence increases with the number of replicates (Figure 4i). This suggests a means to better characterize the authenticity of each candidate site. Based on these observations, we more stringently defined the degree of confidence for each candidate Ψ site based on the stop ratio and the number of replicates the site can be repetitively identified in. Among the 10106 sites identified from HeLa cells, 4260 are defined as "highest-confidence" sites as they fulfil both criteria of being identified in all three biological replicates and having stop ratios higher than 0.3, 3801 sites are defined as "higher-confidence" sites for fulfilling one of the criteria, and 2044 are defined as "lower-confidence sites" for fulfilling none of the criteria (Figure 4j and Lines 329-336).

The assignment of confidence, coupled with the enhanced accuracy in estimating the modification levels as addressed in the response to question #2 from reviewer #1, provides valuable insights for characterizing each individual candidate site.

Upon inspecting raw signals in BID-seq for 6,333 ELAP-seq-only higher or highest-confidence sites, we found that 3,012 sites showed deletion ratio of higher than 0.05 in at least one replicate of BID-seq, thus these sites demonstrate modification signals in BID-seq as well (Figure S10j). Among the rest sites, 2,139 sites have deletion ratio of between 0.0 and 0.05 in BID-seq with 82% of them having average deletion ratios among three replicates of between 0.02 to 0.05, which is below the deletion ratio cutoff used by the method (Figure S10j). There are 1,182 sites having deletion ratio of 0 in BID-seq data, and these sites exhibited substantially lower read coverage than sites detected by BID-seq, suggesting that these sites were missed by BID-seq due to low gene expression (Figure S10k). This further supports the authenticity of sites identified by ELAP-seq and underscores the high sensitivity of our approach in validating weak signals in BID-seq.

In addition to more thorough data analysis, we also performed CLAP and CMC-RT-qPCR validation for more sites. In addition to the ten sites presented in the previous version, we evaluated 1 more site using CLAP (Figure S9c) and 7 more sites using CMC-RT-qPCR (Figure S7f). Overall, 18 sites were successfully validated, including 10 sites not previously identified by any method.

We have incorporated the above results in the revised manuscript (Lines 398-433), as well as detailed discussions for the origins of false positives and false negatives to address concerns users might have when approaching our dataset or using our method (Lines 573-607).

In response to reviewer's suggestions, we employed siRNA knockdown (KD) of TruB1 and DKC1 and strived to assess the dependence of our reported sites on PUS to further prove their authenticity. Unfortunately, we have encountered unforeseen technical issues which have delayed our data acquisition. We believe that the significant improvements we have made would have already sufficiently addressed the concerns raised by the reviewers. Therefore, we are submitting this revised manuscript without the inclusion of data from the PUS KD studies.

However, we are still actively acquiring data from the PUS KD experiments and will be willing to provide the data if the reviewers see them remain necessary and critical.

We believe that this revised manuscript has been substantially improved by: 1) a more rigorous characterization of the confidence level for each candidate site; 2) a comprehensive comparison of our dataset with PRAISE and BID-seq to address the discrepancies; 3) improved accuracy in quantifying the modification levels; 4) thorough evaluation of the reproducibility of our method; 5) site-specific validation of more sites not captured by other methods; 6) detailed discussions of the origins of false negatives and false positives; as well as 7) streamlined pipeline with a single command for user-friendly analysis. We believe these revisions have greatly increased the usability of our dataset and the reliability of our method.

In summary, our revision includes additional experiments, meticulous data analysis, and detailed discussions, reinforcing the validity of the identified sites. Moreover, our method excels in detecting sites overlooked by other methodologies albeit may be less effective in identifying sites in less favorable sequence contexts, especially those with low modification levels. We posit that our method represents a potent innovation and serves as a crucial complement to the existing toolbox for RNA modification detection and investigation.

2. The authors claim that 1/3 of sites have over a 50% modification rate, much higher than the average mRNA modification rates estimated from other methods such as BID-Seq, and inconsistent with their claim that the massive number of new sites are from enrichment of lowly-modified positions. The authors must more rigorously demonstrate that their estimation of modification occupancy is accurate, by correlating their estimates with estimates from mutational-profiling methods such as BID-Seq, and by demonstrating correlation with many more than 2 sites estimated by CLAP. Also, the CLAP vs ELAP-Seq comparison is difficult to see in figure 4h and should have its own figure panel (with the additional comparisons).

Our response:

Thank the reviewer for this constructive critique. In response to the reviewer's critique, we conducted a correlation analysis comparing the quantification results obtained from ELAP-seq with those from CLAP and PRAISE (Lines 379-397). We performed CLAP and obtained absolute quantification for one mRNA modification site (Figure S9c). In addition, there are 9 other mRNA modification sites quantified by CALP previously. All these 10 mRNA sites are in non-ΨU sequence context. Notably, we observed a strong correlation between ELAP-seq and CLAP quantifications for these sites (r = 0.83) (Figure 4m). Additionally, we correlated quantification by our method to that by PRAISE due to a high number of overlapping sies. While the correlations for sites within non-ΨU contexts were moderate (r = 0.46-0.56), the correlation

was high for sites within ΨU contexts (r = 0.72) (Figure S9d). These results, combined with the observation that highly modified rRNA sites are distributed at the upper end of the quantification spectrum, underscore the general accuracy of quantification by ELAP-seq.

Our method relies on adjusted enrichment levels to estimate the relative modification levels, rather than determining exact modification ratios, a limitation of pull-down based strategies. Nevertheless, by utilizing the absolute quantification provided by CLAP (for non-ΨU context sites) and PRAISE (for ΨU context sites) as benchmarks, we were able to estimate the approximate modification levels more confidently in this revised manuscript. This analysis revealed that approximately 20-25% of sites exhibit moderate modification levels (between 30%-60%) or high modification levels (exceeding 60%), while over 70% of sites are characterized as lowly modified (below 30%) (Figure S9e). These results align more closely with conclusions drawn from other studies (PMID: 36997645 and 36302989). Such semi-quantitative estimations of modification levels, coupled with the capacity to probe modification level changes across conditions, would render our method applicable to a wide range of studies.

With the improved quantification accuracy, we re-examined enriched GO terms for genes containing moderately and highly modified Ψ, and found a notable enrichment of categories related to mitochondrial electron transport and ATP synthesis (Figure 5e) (Lines 459-470). This finding aligns with the report that PUS1 is associated with mitochondrial myopathy and calls for future investigation into the roles of Ψ in mitochondrial function and associated diseases.

3. The language explaining the rational for cutoffs and filtering is vague. Please provide specific details with statistical justification for the following: "We optimized cutoffs for stop ratio and reads coverage and allow for gradients of coverage for different ranges of stop ratios" [275-276] "We required higher reads coverage for lower levels of stop ratios to decrease false positives" [685-686] "We allowed slightly lower reads coverage for sites in the out-group" [688-689] "…we screened cutoffs for stop ratios and coverage to find a balance between the number of sites identified and percentage of U" [689-691]

Our response:

We appreciate the reviewers' feedback regarding the clarity of cutoffs and filtering rationale. The cutoffs were determined empirically. Initially, we set a stop ratio of 0.1, guided by our visual examination of IGV, which indicated that some clustered sites tended to exhibit low stop ratios. Additionally, we allowed a read coverage of 5, a practice used in several other studies (PMID: 31548705 and 26075521). However, upon using the percentage of U as an indicator, we found these cutoffs to be suboptimal (U% = 81%). Consequently, we further refined them with more stringent cutoffs. The rationale behind this optimization was requiring higher read coverage for sites with lower stop ratios. In addition, in-group sites situated inside of the enrichment peaks typically had higher read coverage, thus necessitating overall higher coverage thresholds for

these sites. As the number of sites detected decreases with increasing U%, we settled on cutoffs which gave U% of 87.9%. The script used has been provided in GitHub and the cutoffs can be further user-defined for desired confidence. For example, users may raise the cutoffs for stop ratio to filter for sites with higher confidence.

We have now provided the specific details in the Materials and Methods section (Lines 875-880):"…The optimized process was as follows: 1) we required higher reads coverage for lower levels of stop ratios to decrease rate of false positives: In-group: at least 6-read coverage for sites with stop ratios > 0.8, at least 8-read coverage for sites with stop ratios ranging between 0.2 and 0.8 , and stopped reads of at least 11 for sites with stop ratios ranging between 0.1 and 0.2; Out-group: at least 5-read coverage for sites with stop ratios > 0.8, at least 7-read coverage for sites with stop ratios ranging between 0.2 and 0.8 , and stopped reads of at least 2 for sites with stop ratios ranging between 0.1 and 0.2; …"

4. For the IFN dataset it is mentioned that "the interferon stimulated samples (IFN+) resulted in ~40% less reads compared to untreated samples (IFN-), thus we allowed lower reads coverage cutoff for the IFN+ samples."[715-719] How does this not increase the false positive rate for the INF+ samples – if the read cutoffs were set to minimize false positive rate? Intuitively this cutoff should not be determined by read depth. This comparison would be more accurate with the INF-samples randomly sub-sampled down to the same depth as INF+, using identical read cutoffs.

Our response:

This is a great point. In the previous version of the manuscript, we implemented a stricter cutoff for IFN- samples and a looser cutoff for IFN+ samples, based on the established thresholds used for HeLa and HEK cell lines. As noted by the reviewer, this adjustment could lead to an increased rate of false negatives and false positives for each sample group, respectively. In this revised manuscript, we reverted to the original cutoffs, resulting in the identification of 9117 and 3737 candidate sites from IFN- and IFN+ samples, respectively (Figure 6c and Lines 498-501).

To ensure more accurate comparisons, we conducted a reanalysis of the data by employing an equal number of sub-sampled reads for both IFN- and IFN+ samples. Utilizing the same pipeline with consistent read cutoffs, we reanalyzed data subsampled from reads of libraries constructed with SuperScript III, or combined libraries built with SuperScript III and SuperScript IV. In both scenarios, we observed a 40~50% reduction in identified sites from the IFN+ samples compared to the IFN- samples (Figure S12b and lines 501-503). This finding supports a consistent trend of downregulation of Ψ following interferon treatment, which aligns with observations made using LC-MS/MS.

Minor critiques:

1. “a significant proportion of Ψ sites in mRNA appear in the 5’-ΨU-3’ context…” [203-204] what proportion exactly?

Our response: Based on the paper of Li et al., ~30% of Ψ sites appear in the 5’ ΨU 3’ context. We have changed our main text to provide a more precise description (Lines 209-210).

2. “30 – 60% of Ψ in cellular mRNA can be labeled by this enzyme” [205-206] Do you expect 30-60% of Ψ positions are being labeled – or each site is being labeled at a 30-60% rate?

Our response:

We apologize for the confusion caused by this sentence. The ratio 30-60% is m1Ψ/Ψ percentage determined through LC-MS/MS analysis on digested RNAs. Due to the sequence preference of the enzyme, the efficiency of enzymatic labeling should be higher for Ψ sites in the 5’-ΨU-3’ context compared to sites in other sequence contexts. Ψ sites in the 5’-ΨU-3’ constitutes around 30% of all Ψ sites in mRNA while Ψ sites in other sequence contexts constitutes about 70%. Therefore, more than 30% of Ψ sites would undergo labeling, with the degree of labeling at individual positions contingent upon the specific sequence context, which together gave an overall labeling efficiency of 30-60% across all Ψ sites.

We have modified our main text to clarify this (Line 139): “The overall 33-60% $m^1\Psi/\Psi$ ratio suggests that a considerable proportion of Ψ modification sites were successfully labeled.”

3. Why is there a stop in untreated RNA at the expected psi position in figure 2e?

Our response:

We appreciate the reviewer's observation, and upon reflection, we acknowledge that our manuscript lacked clarity in describing the experiment's design.

In our assay, we used a synthetic 33-mer RNA as the template, which harbors a Ψ modification in the middle. A fluorescently labeled RT primer was designed to target the position immediately downstream of the modification site. The RT primer and template were mixed at a 1:10 ratio to drive most primers to hybridize with the template. In the absence of any treatment, the RT primer reads through the modification site, producing the full-length RT product (upper FL band). Conversely, when encountering the propargyl-Ψ adduct (with or without subsequent

biotinylation at a low dNTP concentration), the elongation of the RT primer is impeded, resulting in a significant amount of un-extended RT primer (lower Primer band in Figure 2e).

The anticipated stop signatures in treated RNA align precisely with the expected Ψ position. The limited presence of un-extended RT primer bands in untreated RNAs is likely attributed to a low level of unhybridized RT primer (Figure 2e, lane 2 and lane 6). Notably, the un-extended RT primer bands in treated RNAs exhibited considerably stronger signals compared to those in untreated RNAs (Figure 2e, lane 3-5 compared to lane 2, and lanes 7-9 compared to lane 6). This disparity indicates that the introduction of bulkier groups during Ψ labeling significantly increased the arrest rate of reverse transcription at the modification site, aligning with our expectations.

To make the experimental design clearer, we have incorporated additional details in the legend of Figure 2 and in the Materials and Method section (Lines 714-724).

4. How do the NNpsiNN pools perform in the standard library prep? E.g. are there any RT-stop or ligation biases due to sequence context?

Our response:

We appreciate the reviewer's insightful question. In assessing the sequence preference of enzymatic labeling with the NNpsiNN pools, we employed a protocol designed to avoid generating RT stops so that we could comprehensively evaluate the sequence context spectrum on both sides of the modification site. Specifically, following on-bead pull-down, we eluted RNAs from the beads by heat-denaturing the streptavidin-biotin interaction. Subsequently, we employed Superscript IV with 0.5 mM final dNTP to facilitate full readthrough at the modification site. Additional details about this methodology have been incorporated into the Materials and Methods section for clarification (Lines 816-817).

Our ELAP-seq results consistently identified uracil as the primary nucleobase, aligning with the in vitro characterization of Mj1640's specificity towards Ψ. This evidence supports the conclusion that ELAP-seq specifically detects Ψ sites in RNA. We want to emphasize that, in our library preparation workflow, we do not perceive sequence context-dependent biases in RT-stop or ligation. While previous studies have reported sequence biases for 3' RNA adapter ligation using T4 RNA ligase 2 truncated KQ, we mitigated this bias by employing RNA adapters with multiple Ns on the 5' end, as recommended (Nucleic Acids Research, 2011, Nov; 39(21):e141). Our analysis of input libraries using the same pipeline revealed no enrichment for uracil, demonstrating that ligation and reverse transcription were not influenced by sequence context.

5. It is not described how the wobble pair analysis was performed, please add more detail in the methods or main text.

Our response:

We thank the reviewer for pointing this out. We have now provided a more detailed description of the analysis in the Materials and Methods section (Lines 934-946). We have also deposited the data analysis pipelines and the in-house python transcripts to GitHub.

"RNA sequences of 100 nt surrounding all Ψ sites located in different mRNA subregions (5' UTR, 3' UTR and CDS) with 50 nt upstream and 50 nt downstream were used for RNA secondary structure prediction. The program viennaRNA was used with the command of 'RNAfold -p < input.fa > output.res'. The resulting file was cleaned up in notepad++ to keep the information of the topmost prediction for each site. Then, we used an in-house python script match.py to extract the information of the base pairing status of the modification site and the location of its matched base if it is base paired. We then determined the identity of the matched base for each base-paired modification site using the 'MID' function in excel. Next, we calculated the frequency of the modification site residing in a mismatched/UG wobble status versus in UA matched status. Control U sites were chosen at both the -20 position and +20 position of Ψ sites and analysis were performed the same way as for Ψ sites. mfold were used to draw the secondary structures of 100 nt surrounding the chosen Ψ sites."

6. Enrichment of Ψ at splice junctions is only informative with a comparison against the background distribution of U. Plot both distributions from your dataset and demonstrate that the difference in Ψ distribution is significant.

Our response:

We express gratitude to the reviewer for providing valuable insights into the analysis. In response to the suggestion, we randomly selected 10,000 U sites from the transcriptome and conducted a parallel analysis. To our disappointment, these randomly chosen sites were also found to be situated within 100 nt of the nearest splice sites. To delve deeper into this observation, we computed the average distance to splice sites within 800 nt, 400 nt, 200 nt, and 100 nt windows, and still, no substantial differences were observed between the Ψ group and the control U groups across any of these window sizes. We regret the oversight that led to an incorrect conclusion in the previous version of the manuscript. To rectify this, we have removed the erroneous figure and texts that depicted the correlation of Ψ with splicing.

Reviewer #3 (Remarks to the Author):

The manuscript by Wang et al developed ELAP-seq to enrich and map Ψ at single-base resolution. This new Ψ sequencing technology leveraged enzymatic strategy to label and enrich Ψ-modified transcripts. They have identified approximately 10, 000 candidate Ψ sites in HEK293T and HeLa cell lines, a significantly larger number compared to all previous CMC-based and bisulfite-based methods. Based on this approach, the authors found differential Ψ modification upon type-I interferon stimulation in the glioblastoma cell line. Overall, the manuscript provides a new sequencing method and applies it to detect Ψ modification in the human transcriptome. Unfortunately, the major concerns remain non-specificity, unclear false positives, and unclear false negatives. Some weaknesses need to be addressed before this manuscript would be suitable for publication:

We thank the reviewers for the positive comments about our method and the constructive critiques. However, we respectively disagree with the reviewer's opinion that our method lacks specificity. We have improved and demonstrated the specificity of our method in various ways: 1) we have performed thorough in vitro characterization of the enzymatic labeling; 2) we have optimized the pull-down protocol to achieve highly specific enrichment of Ψ-modified RNA; 3) we have obtained agreement of results between ELAP-seq and MALDI on the synthetic oligo pools covering 256 sequence contexts; and 4) we have specifically identified known modification sites on rRNA. Additional support for the accuracy of our method includes that: 1) the sequence context of the identified Ψ agrees with the sequence preference of the Mj1640 enzyme; and 2) U is predominant among all sites identified from the pull-down samples but not the input samples.

Major:

1. The authors reported around ten thousand candidate Ψ sites from HeLa and HEK 293T cell lines. However, more than 9000 sites currently lack validation through existing datasets. Before asserting their claims of high sensitivity, the authors have to verify the specificity of ELAP-seq instead of just providing the ambiguous Ψ lists. The authors should perform perturbation of PUS proteins, such as employing PUS knockdowns or knockouts in these cells, to assign the majority of the newly identified Ψ sites to specific enzymes. This assignment would validate the specificity of ELAP-seq and contribute to functional insights.

Our response: Please refer to our response to Major question #1 from reviewer #1 for a more detailed response to this question.

2. A more comprehensive comparison should be performed between the Ψ dataset sourced from HEK293T and HeLa cells in ELAP-seq and their previously published human mRNA pseudouridine maps (Dai, Q. et al. Nat Biotechnol. 2023). They should analyze the raw deletion signal for ELAP-seq-unique Ψ sites in BID-seq classify the authenticity of these sites and provide a clear explanation for their lack of detection in BID-seq.

Our response:

This is a great point. We thank the reviewer for the constructive suggestion. We have performed more analysis in response to the reviewer's suggestion, and we found:

Of the 6,333 ELAP-seq-only (not detected in any previous reports) higher- or highest-confidence sites (stop ratio > 0.3 in two replicates and/or detected in all three replicates) for HEK 293T, 3,012 sites showed deletion ratio of higher than 0.05 in at least one replicate of BID-seq, thus these sites demonstrate modification signals in BID-seq as well (Figure S10j). Among the rest sites, 2,139 sites have deletion ratio of between 0.0 and 0.05 in BID-seq with 82% of them having average deletion ratios among three replicates of between 0.02 to 0.05, which is below the deletion ratio cutoff used by the method (Figure S10l). There are 1,182 sites having deletion ratio of 0 in BID-seq data, and these sites exhibited substantially lower read coverage than sites detected by BID-seq (Figure S10k-l), suggesting that these sites were missed by BID-seq due to low gene expression. This further supports the authenticity of sites identified by ELAP-seq and underscores the high sensitivity of our approach in validating weak signals in BID-seq.

We have added these results to the section "Comparisons to other methods" in the main text.

3. In L187, the strong bias for ΨU context might even underestimate the GUΨC context modified by TRUB1 protein which has the highest modification activity, which indicates the severe false negative of ELAP-seq. The authors need to demonstrate they could detect all these TRUB1-dependent sites from previous Ψ datasets.

Our response:

We conducted a comparison between our dataset and the 324 TRUB1-dependent sites documented by PRAISE and added the results to the section "Comparisons to other methods" in the main text. Our analysis revealed the identification of 223 out of the 324 TRUB1-dependent sites (68.8%) in our dataset (Figure S10h). Notably, the sites that went undetected tended to exhibit lower coverage and deletion ratios in the PRAISE dataset (Figure S10i), implying inherent challenges in detecting these sites and lower confidence compared to the remaining sites. It's important to note that PRAISE employed a sequencing depth 30 times greater than our

method, enabling the detection of lowly expressed genes that our protocol might have missed. It is possible that our method could identify all these sites with deeper sequencing.

We acknowledge a limitation in our method arising from the enzyme's sequence bias, which may result in missed sites in non-ΨU contexts, especially when expression or modification levels are low (Figure S10f-g). However, the limitation is not significant based on the result above. We have provided discussions on the originalities of false negatives in the Discussion section in this revised manuscript.

4. The authors claim the high sensitivity and low background of ELAP-seq; however, they have yet to provide substantial supporting evidence for this assertion. (1) For Ψ in rRNA, they only identified 70 out of 104 known Ψ sites in rRNA (L250), since Mj1640 the strong preference for 5'-ΨU-3' sequence context in oligo RNA, they should analyze these 34 missed known Ψ sites, and then address whether these omissions were a result of the sequence context instead of being clustered within specific rRNA regions. (2) For Ψ in mRNA, although the author can filter out these highly modified Nm sites through bioinformatic cutoff, they cannot exclude the existence of false-positive Ψ sites derived from Nm modifications that vary with differential modification levels in mRNA, hence, the reliability of ten thousand of Ψ is questionable. The comparison and overlap analysis of Ψ sites identified via ELAP-seq with Nm sites identified through Nm-seq should be performed (Dai, Q., et al Nat Methods, 2017).

<u>Our response:</u>

We would like to first define the high sensitivity we claimed in the manuscript. Our method excels in enriching modified RNA, enabling the detection of sites in lowly expressed genes with a shallower sequencing depth. This enrichment of modified RNA enhances the visibility of stop signals, facilitating the detection of even lowly modified sites. Therefore, we claimed that our method has high sensitivity to detect lowly modified sites and sites in lowly expressed genes. We admit that our method has lower resolution compared to others in terms of detecting highly clustered modification sites due to the stop signature.

In addressing instances where known Ψ sites in rRNA were not detected, we observed that these sites are located within 20 nt of the nearby major modification sites (Figure S5c). Therefore, the stop signal at those sites were obscured by either coverage overlap with nearby sites or low coverage downstream of a major site. Several sites exhibited persistent stop signals across replicates but were filtered out due to the stop ratio cutoff of 0.1. We note that this situation is improved with messenger RNA since modifications are less clustered in that context.

Regarding the potential Nm sites, we clarify that those sites were filtered based on the presence of stop signature at the same location in the input sample. If the modification level is too low to

be detected in the input, it wouldn't manifest in the pull-down sample, thereby avoiding false positives in our final list. While we acknowledge the rare possibility of Nm coexisting with Ψ resulting in an increased stop signal during pull-down, a comparison with Nm data revealed no overlap. The reliability of the ten thousand candidate sites is discussed in greater detail in other responses.

5. In L267 and Figure 4a, how does the mitochondrial mRNA Ψ result compare to the previous Ψ modification map obtained by BID-seq? The comparison of mitochondrial Ψ sites should be performed for both HEK293T and HeLa cell lines.

Our response: In our analysis, we identified 20 candidate Ψ sites in HeLa mitochondria mRNA and 27 in HEK mitochondria mRNA (Supplementary table 3-4). Notably,15 sites were shared between the two cell lines. This set of sites encompassed all five mitochondria mRNA sites for HEK 293T (chrM: 6293, 7329, 9905, 11154 and 14868) and three for HeLa (chrM: 6293, 7329, and 11154) collectively identified by BID-seq and PRAISE. It's worth noting that most of the mitochondria mRNA sites uniquely detected by ELAP-seq are characterized as lowly expressed or lowly modified, suggesting potential reasons why they were not detected by the other two methods.

6. In L328, the authors claim “excellent” repeatability for Ψ detection in mRNA. They should provide mRNA Ψ site numbers for three biological replicates and hence the ratio of overlapping sites.

Our response:

We appreciate the reviewer's valuable suggestion. The reproducibility of our method can be assessed from two primary perspectives: 1) the ability to consistently identify the same enriched peaks or candidate sites across multiple replicates; and 2) the degree of consistency across these replicates in terms of pull-down or enrichment levels at each candidate site. While the former parameter may be susceptible to batch effects due to varying amounts of starting material and technical fluctuations, the latter parameter is intrinsic to the method itself. In response to the reviewer’s suggestions, we have performed the third replicate for both HEK 293T and HeLa cells to interrogate the reproducibility of our method.

To evaluate the method's ability to consistently identify the enriched peaks or candidate sites, we employed MACS2 for peak calling, a method recognized for its reliability in this context (PMID: 32313079). Among the three replicates, we identified 5,558, 5,114, and 4,703 enriched peaks, respectively (Figure S8a). Notably, 2,636 peaks (47.4% - 56% of peaks in each replicate) were shared across all three replicates (Figure S8a). Additionally, 67% to 81% of peaks identified in each replicate were shared by at least one other replicate (Figure S8a).

Recognizing that enriched peaks predominantly cover sites in favorable sequence contexts, we expanded our analysis to identify candidate sites from both inside and outside these enriched peaks. To mitigate noises due to non-specific binding during pull-down, we considered a site as a candidate modification site that has a stop signature occurring in at least two independent biological replicates. Applying these criteria, we identified 10,077 candidate sites from two replicates of HEK 293T RNAs. Notably, we found that 6,108 (~61%) of the 10,077 sites were detected by the third biological replicate, accounting for 97% of all stopped sites shared by all three replicates (Figure S8b). Taken together, these findings indicate a high level of reproducibility with respect to the first parameter.

To investigate the second aspect of reproducibility, we evaluated the correlation of pull-down levels (RPM) across multiple independent biological replicates. Focusing on candidate sites shared by all three replicates, we assessed the coefficient of variation (CV) of their RPM values. Notably, the CV predominantly fell within the 0-25% range across the three independent pull-down samples, indicating a high level of consistency (Figure S8c). Importantly, this consistency across the pull-down samples surpassed that observed across the input samples (Figure S8d), underscoring the high reproducibility of our method.

In summary, with an additional biological replicate we have demonstrated the good reproducibility of our method, both in terms of consistently identifying the same peaks or sites and maintaining a high level of consistency in pull-down efficiency across replicates.

We have added a paragraph to the main text to discuss the reproducibility (Lines 321-329).

7. In L432, the authors need to provide more experimental and bioinformatic analysis for 92 downregulated Ψ sites upon interferon-α treatment, for instance, they can analyze the motif and investigate whether they were modified by PUS3 protein. The authors could also detect the protein level of relevant PUS enzymes between IFN- and IFN+ samples to explain why Ψ was downregulated.

<u>Our response:</u>

We thank the reviewer for the insightful comments. Upon scrutinizing the sequence context of the 126 downregulated sites, we observed a consistent pattern aligned with the overall sequence preference of the enzyme, emphasizing a preference for U on the 3' side. This finding suggests that the sequence context might be influenced by the enzyme's inherent preferences, limiting its ability to provide substantial insights into whether these sites are PUS3 targets.

To delve further, we conducted Western blot analyses to assess the expression levels of relevant PUS enzymes—PUS1, PUS3, TruB1, PUS7, PUS10, and DKC1—during IFN stimulation. However, no discernible changes were observed in the expression levels of any PUS proteins

(Figure S12j). This leads us to infer that the downregulation may stem from regulatory mechanisms acting on Ψ or Ψ synthases, rather than alterations in the expression levels of the writer proteins themselves (Lines 527-533).

Minor:

1. In L55, the statement regarding quantitative mapping methods is incorrect, RBS-seq is not a quantitative approach.

Our response: We have fixed our main text: "Examples include recently developed methods RBS-seq, BID-seq and PRAISE, which rely on bisulfite treatment and allow qualitative or quantitative mapping of Ψ transcriptome-wide" (Lines 53-54)

2. **In L130, data was not shown for the control U-RNA.**

Our response: We have added the data for the control U-RNA in figure 1.

3. In L146, the authors should provide experimental data to demonstrate the capacity of the **Mj1640 enzyme to label the synthetic 8-mer oligo using propargyl-SeAm.**

Our response: We have presented the MALDI data which demonstrated Mj1640's capability to label a synthetic 8-mer oligo using propargyl-SeAm (Figure S1d). However, the labeling efficiency on this RNA was comparatively lower than the reaction involving SAM or the reaction on the synthetic 17-mer oligo (Figure S1d and Figure 1c). We reasoned that this reduced efficiency is attributed to the suboptimal length of the 8-mer oligo for Mj1640 and the weakened binding affinity of Mj1640 to the sterically bulkier propargyl-SeAm. These factors overall make the reaction fall below the enzyme's optimal requirements for efficient binding and catalysis. Importantly, we do not anticipate this to be a concern when working with biological RNAs, as we typically fragment them into 60-200 nt. Indeed, we have observed efficient labeling on cellular RNA as well as a 17-mer RNA oligo using propargyl-SeAm (Figure 1c).

4. In L158, the **size distribution of untreated mRNA should be provided.**

Our response: We have included the size distribution of untreated mRNA in Figure S3a. Following enzymatic treatment, a slight shift to lower RNA sizes was observed. Importantly, the degradation was mild, as evidenced by a high RNA recovery rate ranging from 60% to 80%.

We thank the reviewers for their constructive comments and for bringing up critical questions regarding the reliability, reproducibility, and quantitative capability of the ELAP-seq method. To address these comments, in this revised version of the manuscript, we have:

- Performed a negative control using in vitro transcribed RNA made from cDNA of HEK293T mRNA which lacked any modifications. The data showed minimal background and little overlap with candidate sites identified from native biological RNA samples (Figure 4g and the new supplementary Figure S9).
- Performed knockdown experiment for one of the pseudouridine writers, DKC1, which led to the identification of 1014 sites confidently downregulated due to DKC1 depletion, including 882 sites newly identified by ELAP-seq (Figure 4h and the new supplementary Figure S10).
- Performed CMC-RT-qPCR to validate more candidate sites, including 9 sites uniquely identified by ELAP-seq (Figure S11).
- Evaluated the reliability of individual candidate sites by rationally categorizing them into different confidence levels, based on the parameter of stop ratio and repeated identification, which are important indicators of reliability (the new supplementary Figure S12 and Supplementary Tables 1 and 2).
- Compared the quantification by ELAP-seq with more quantitative methods CLAP and PRAISE (Figure 4i-j and S13). These comparisons enabled more accurate estimation of modification levels, allowing us to categorize candidate sites into various modification-level brackets (Figure S13 and Supplementary Tables 1 and 2). This analysis supports a more confident conclusion that around 75% of sites identified by ELAP-seq fall into the low-modification category (estimated modification level < 30%), consistent with conclusions from other methods.
- Performed a detailed comparative analysis with the bisulfite-based methods, which addressed discrepancies between ELAP-seq and other methods by showing that the majority of sites uniquely identified by either method exhibit below-cutoff but discernible signals in each other's datasets. This suggests the discrepancies mainly stem from sites that are intrinsically more difficult to detect due to low modification or expression levels (the new supplementary Figure S14).
- Added a third biological replicate for both HeLa and HEK293T cell lines, which showed that > 60% of candidate sites identified by the first two biological replicates were detected by the third replicate. Additionally, the pull-down RPM values are highly consistent across the three replicates (the new supplementary Figure S8).
- Provided a clear discussion of the advantages and limitations of the ELAP-seq method compared to other methods (page 29-30)
- Provided a clear discussion of the origins of false positives and false negatives of ELAP-seq and proposed ways to further improve it (page 30-31).
- Improved the data analysis pipeline for more streamlined and simplified analysis without sacrificing the rigor of the pipeline (Methods section).
- Re-analyzed the IFN-/+ datasets using randomly sub-sampled reads with the same depth (Figure 6b-c). Further explored the expression changes of pseudouridine synthases during IFN stimulation (Figure 6e).
- Revised the manuscript to clarify previously ambiguous details and rationale and to strengthen the reliability of the method by highlighting the concordance of ELAP-seq results with orthogonal methods and advantages over similar approaches.

In summary, we believe that the results obtained with the ELAP-seq method are now thoroughly validated and that the manuscript has been substantially improved. All the changes made to the manuscript are labeled in **blue**. Below, we provide a point-by-point response to the reviewers' specific comments.

REVIEWER COMMENTS

REVIEWER COMMENTS

Reviewer #1 (Remarks to the Author):

Pseudouridine (Ψ) is an abundant and critical RNA modification, yet the full extent of its biological roles in the cell have been difficult to understand due to incomplete transcriptome-wide maps of modification sites. One of the major bottlenecks in generating comprehensive maps with current mapping methods is the extensive sequence depth required to confidently assign modifications globally, due the sparse distribution and general low occupancy of Ψ. In this manuscript, Wang et al., present an innovative method to enrich for RNA fragments containing Ψ by using an archaeal methyltransferase, which they claim can specifically add click-chemistry compatible propargyl groups to Ψ to facilitate biotin conjugation. This addition allows them to both enrich for modified RNA using streptavidin, and obtain single nucleotide resolution of modification position using RT-stop signatures induced by the biotin adduct. They apply this method to correctly identify most known rRNA pseudouridine sites and generate a map of pseudouridylation in human mRNA across three cell types and make several claims about global Ψ distribution.
While we find this technique to be a potentially powerful new tool for profiling Ψ, the lack of overlap with existing methods and little validation for the many new sites uncovered is cause for concern. Additionally, several of the claims made about Ψ occupancy and distribution need more supporting evidence or explanation. We do not recommend acceptance of this manuscript until the following points are addressed:

Major Concerns:

1. According to figure S11, only 4% of sites identified in HeLa cells overlap with previous mapping datasets, and 8% overlap in 293T. The authors do not address this discrepancy beyond this figure, except to suggest it is due to poor performance of CMC-based methods.
More evidence is needed to demonstrate that the mRNA data is capturing mostly genuine Ψ modifications, including performing PUS enzyme depletions to demonstrate PUS-dependence of signal, and far more targeted validations (ex. using CLAP), specifically of sites not recapitulated by other datasets.

Our response:

We thank the reviewer for the constructive feedback.

In response to the reviewer's comment, we performed a more comprehensive overlap analysis with three most recent bisulfite-based methods (BID-seq, RBS-seq, PRAISE) and a sensitive

CMC-based chemical pull-down method (CeU-seq). Our data showed substantial overlap: 40.5% with BID-seq, 49% with RBS-seq, 53% with PRAISE, and 32% with CeU-seq (Figure S14a). Overall, our sites overlap with 1,448 of the 3,818 sites (37.9%) collectively identified by PRAISE, BID-seq and CeU-seq in HEK 293T cells. These overlapping sites account for 17.8% of all sites (8255) identified by ELAP-seq in HEK293T; the seemingly low percentage of overlap is largely due to a high number of new candidate sites uniquely identified by our method. We will provide more evidence to support the validity of these new sites as below.

With a more accurate estimation of modification level (please see response to question #2), we categorized candidate modifications sites into various modification levels (page 21 and Figure S13). We found that the newly identified sites are predominantly lowly modified compared to the ones overlapping well with other methods (Figure S14h). Similarly, sites uniquely identified by other methods (e.g., PRAISE) also showed lower modification levels and read coverage compared to the rest sites in their datasets (Figure S14e). This suggests that discrepancies between different methods mainly stem from sites with low modification levels or expression levels, and these sites are intrinsically more difficult to detect.

To validate the new sites we identified, first, we performed a negative control experiment using modification-free IVT RNA generated from cDNA of HEK293T mRNA (Figure 4g and S9). These RNA samples represent the mRNA transcriptome of HEK293T cells but lack any modification. ELAP-seq on these samples yielded much less sites, which also had minimum overlap with sites detected from native biological RNAs (Figure 49). This supports that most signals we identified in native RNAs are true modifications, rather than artifacts from the labeling or sequencing process.

In addition, as suggested by the reviewers, we performed a knockdown experiment of **DKC1**, which was reported to affect a large fraction of mRNA modification sites (Zhang et al, Nature Chemical Biology, 2023, 19, pages1185–1195). To ensure technical consistency during pull-down and subsequent steps, we employed internally barcoded RNA adapters and pooled control and knockdown samples during library preparation. ELAP-seq analysis of knockdown and control samples identified **1014 sites as DKC1-responsive** (p-value < 0.05 and enrichment level decrease > 2-fold), including **882 new sites** reported here (Figure 4h, S10 and Supplementary Table 5). While this experiment confirms a substantial number of new sites, we acknowledge the limitation of our method's sensitivity in detecting all subtle stoichiometric changes at lowly modified sites, and therefore not all new sites could be experimentally verified through knockdown.

Nevertheless, by analyzing the raw signals of the new sites in the BID-seq dataset, we found that **55.7% of the new sites showed deletion ratios higher than 0.05** in BID-seq dataset but below the 10% cutoff set by BID-seq; thus, these sites demonstrate positive signals despite "not" identified by BID-seq. There are **additional 26% of new sites showing deletion ratios above 0** in BID-seq data. **Only 17.8% of the new sites have no deletion signals in BID-seq** and these sites exhibited significantly lower reads coverage in the BID-seq dataset compared to the overlapping sites, suggesting that **they were likely missed due to low gene expression or they are intrinsically less confident** (Figure S14i-k).

Finally, to facilitate the interpretation of individual site's reliability, we rationally categorized candidate Ψ sites detected by ELAP-seq into different confidence levels (page 18) based on the RT stop signal strength and repeatability of identification. There are 3947 sites categorized as highest-confidence, 2714 as higher-confidence, and 1595 as lower-confidence (Figure S12). We found that sites well overlapping with other methods were mainly found in the highest- and higher-confidence groups (Figure S14b), supporting the validity of this classification. We encourage caution in interpreting sites from the lower-confidence category and recommend further experimental validation. Additionally, we discuss potential sources of false positives and false negatives in our dataset (page 30-31) to guide readers in understanding the method and the resulting data.

2. The authors claim that 1/3 of sites have over a 50% modification rate, much higher than the average mRNA modification rates estimated from other methods such as BID-Seq, and inconsistent with their claim that the massive number of new sites are from enrichment of lowly-modified positions. The authors must more rigorously demonstrate that their estimation of modification occupancy is accurate, by correlating their estimates with estimates from mutational-profiling methods such as BID-Seq, and by demonstrating correlation with many more than 2 sites estimated by CLAP. Also, the CLAP vs ELAP-Seq comparison is difficult to see in figure 4h and should have its own figure panel (with the additional comparisons).

Our response:

As requested by the reviewer, we analyzed the correlation of estimates by ELAP-seq with more sites quantified by CLAP. So far, modification stoichiometry for 10 mRNA modification sites have been determined by CLAP, including one quantified by us during this revision (Figure S13d). Our method detected all 10 sites, and notably, we observed a strong correlation between ELAP-seq and CLAP quantification results (r = 0.83) (Figure 4j). All these 10 mRNA sites are located in non-ΨU sequence contexts, supporting reliable semi-quantitative estimation by EALP-seq for modifications in these sequence contexts. We have created a new figure panel for this correlation for better illustration (Figure 4j).

Additionally, we correlated quantification results from ELAP-seq with those from PRAISE, considering a higher number of overlapped modification sites between the two methods. The correlation was strong for sites within ΨU contexts (r = 0.74) and moderate for non-ΨU contexts (r = 0.43-0.56) (Figure S13e).

These results, combined with the observation that highly modified rRNA sites are distributed at the upper end of the quantification spectrum (Figure 4i and S13c), demonstrate the semi-quantitative nature of ELAP-seq.

Our method could only discriminate relative modification levels, and needs to rely on correlation with absolute quantification by other methods to provide semi-quantitative estimations for all sites. Such analyses allowed us to make a more confident conclusion that over 75% of sites are characterized as lowly modified (with estimated modification ratios <30%) (Figure S13f), which

aligns more closely with conclusions drawn from other studies (Dai et al., Nat Biotechnol. 2023, 41(3):344-354 and Zhang et al, Nature Chemical Biology, 2023, 19, pages1185–1195).

We acknowledged the limitation in the quantitative capacity, as it relies on absolute quantification made by other datasets, and the quantification of closely located sites can be skewed by one another. We discussed this limitation in the manuscript and proposed to further improve the quantification capacity by improving the enzyme's sequence compatibility and optimizing reverse transcription conditions for non-mutually intervening signatures such as mutations (page 30).

With the improved quantification accuracy, we re-examined enriched GO terms for genes containing moderately and highly modified Ψ, and found a notable enrichment of categories related to mitochondrial electron transport and ATP synthesis (Figure 5e). This finding aligns with the report that PUS1 is associated with mitochondrial myopathy and calls for future investigation into the roles of Ψ in mitochondrial function and associated diseases

3. The language explaining the rational for cutoffs and filtering is vague. Please provide specific details with statistical justification for the following: "We optimized cutoffs for stop ratio and reads coverage and allow for gradients of coverage for different ranges of stop ratios" [275-276] "We required higher reads coverage for lower levels of stop ratios to decrease false positives" [685-686] "We allowed slightly lower reads coverage for sites in the out-group" [688-689] "…we screened cutoffs for stop ratios and coverage to find a balance between the number of sites identified and percentage of U" [689-691]

Our response:

In this revised version, we simplified the analysis pipeline by applying uniform cutoffs for stop ratio (a minimum of 0.1) and reads coverage (a minimum of 10-read coverage and a minimum of 3 stopped reads). This change was made based on the realization that a lower stop ratio will automatically lead to a higher read coverage if certain cutoffs for the minimum read coverage and number of stopped reads are set. Therefore, the pipeline can be simplified this way without sacrificing its rigor. The cutoff we now apply is based on the criteria used in another pull-down based approach (Li et al., Nature Chemical Biology, 2015, 11, pages592–597). With this simplified cutoff, the number of candidate sites decreased down to ~8000 in both cell lines. Using the fraction of non-U sites as an indicator of false positives, we confirmed that the cutoff does not lead to a higher rate of false positives. The cutoffs we implement set the baseline to identify all candidate sites, and we further increase the threshold of stop ratios to define more confident sites, as discussed on page 18.

We have modified the Materials and Methods section accordingly on page 42.

4. For the IFN dataset it is mentioned that "the interferon stimulated samples (IFN+) resulted in ~40% less reads compared to untreated samples (IFN-), thus we allowed lower reads coverage cutoff for the IFN+ samples."[715-719] How does this not increase the false positive rate for the INF+ samples – if the read cutoffs were set to minimize false positive rate? Intuitively this cutoff

should not be determined by read depth. This comparison would be more accurate with the INF- samples randomly sub-sampled down to the same depth as INF+, using identical read cutoffs.

Our response:

We agreed that the different cutoffs may result in different levels of false positives. In this revised manuscript, we applied a uniform cutoff, resulting in the identification of 7271 and 3649 candidate sites from IFN- and IFN+ samples, respectively (Figure 6b). Recognizing that the discrepancy in the number of identified sites could have resulted from differences in sequencing depth (which we also acknowledged in the revised manuscript), we randomly sub-sampled reads to the same depth for IFN- and IFN+ samples, and repeated the analysis. We still observed a 40~50% reduction in the number of identified sites in IFN+ samples compared to IFN- samples (Figure S16c). We also re-analyzed differential modification with the original sequencing data and randomly sub-sampled reads with the same depth. In both scenarios, we observed a higher percentage of sites that are down-regulated than up-regulated (Figure 6c), supporting a trend of Ψ downregulation following interferon treatment, which aligns with the observation made using LC-MS/MS.

Minor critiques:

1. “a significant proportion of Ψ sites in mRNA appear in the 5’-ΨU-3’ context…” [203-204] what proportion exactly?

Our response: Based on the paper by Li et al. (Li et al., Nature Chemical Biology, 2015, 11, pages592–597), ~30% of Ψ sites appear in the 5’ ΨU 3’ context. We have added this information to the main text.

2. “30 – 60% of Ψ in cellular mRNA can be labeled by this enzyme” [205-206] Do you expect 30-60% of Ψ positions are being labeled – or each site is being labeled at a 30-60% rate?

Our response:

The 30-60% ratio refers to the m1Ψ/Ψ percentage determined by LC-MS/MS analysis of digested RNAs, thus it is the overall labeling rate for all Ψ on mRNA. Due to the sequence preference of the enzyme, we expect that all Ψ positions are labeled, with each site being labeled to a varying degree, which together give an overall labeling efficiency of 30-60% across all Ψ sites.

We have modified our main text to clarify this: “The overall 33-60% $m^1\Psi/\Psi$ ratio suggests that a considerable proportion of Ψ modification sites were successfully labeled.”

3. Why is there a stop in untreated RNA at the expected psi position in figure 2e?

Our response:

We thank the reviewer for the comment. We realize that our original manuscript didn't clearly describe the experimental design. We have incorporated additional details in the legend of Figure 2 and in the Materials and Method section to clarify this. A detailed explanation is as below:

In our assay, we used a synthetic 33-mer RNA harboring a Ψ modification as the template. A fluorescently labeled RT primer targets the position immediately downstream of the modification site. The RT primer and template were mixed at a 1:10 ratio to drive most primers to hybridize with the template. Without any treatment, the modification site can be read through by reverse transcriptase, producing a full-length RT product (upper FL band). When labeled with propargyl-Ψ adduct, the modification impeded the elongation of the RT primer, resulting in a significant RT arrest signal (lower Primer band in Figure 2e). The stop signatures in treated RNA align precisely with the expected Ψ position.

The presence of a small amount of un-extended RT primer in untreated RNAs is likely attributed to a low level of unhybridized RT primer (Figure 2e, lane 2 and lane 6). Notably, the stop signals in treated RNAs are much stronger than those in untreated RNAs (Figure 2e, lane 3-5 compared to lane 2, and lanes 7-9 compared to lane 6), demonstrating that the introduction of bulkier groups at the modification site significantly increased the pausing rate of reverse transcription.

4. How do the NNpsiNN pools perform in the standard library prep? E.g. are there any RT-stop or ligation biases due to sequence context?

Our response:

We appreciate the reviewer's insightful question.

When assessing the sequence preference of the Ψ methyltransferase using the NNpsiNN pools, we employed a protocol designed to avoid generating RT stops, so that we could comprehensively evaluate the sequence contexts on both sides of the modification site. Specifically, following on-bead pull-down, we eluted RNAs from the beads by heat-denaturing the streptavidin-biotin interaction. Subsequently, we employed Superscript IV with 0.5 mM final dNTP to facilitate full readthrough at the modification site. Additional details about this methodology have been incorporated into the Materials and Methods section for clarification.

We want to emphasize that, in our library preparation workflow, we do not perceive sequence context-dependent biases in ligation. While previous studies have reported sequence biases for 3' RNA adapter ligation using T4 RNA ligase 2 truncated KQ, we mitigated this bias by employing RNA adapters with multiple Ns on the 5' end, as recommended (Nucleic Acids Research, 2011, Nov; 39(21):e141). We also do not perceive sequence context-dependent biases in RT-stop either. Our analysis of input libraries using the same pipeline revealed no enrichment for uracil,

demonstrating that both ligation and reverse transcription were not biased by the sequence context.

5. It is not described how the wobble pair analysis was performed, please add more detail in the methods or main text.

Our response:

Thank the reviewer for pointing this out. We have now provided a more detailed description of the analysis in the Materials and Methods section. We have also deposited the data analysis pipelines and the in-house python transcripts to GitHub.

"RNA sequences of 100 nt surrounding all Ψ sites located in different mRNA subregions (5' UTR, 3' UTR and CDS) with 50 nt upstream and 50 nt downstream were used for RNA secondary structure prediction. The program viennaRNA was used with the command of 'RNAfold -p < input.fa > output.res'. The resulting file was cleaned up to keep the information of the RNA sequence and the most confident prediction (the one with lowest ΔG values) for each site. Then, we used an in-house python script to extract the information of the base pairing status of the modification site and the location of its matched base if it is base paired. We then determined the identity of the matched base for each base-paired modification site using the 'MID' function in excel. Next, we calculated the frequency of the modification site residing in a mismatched/UG wobble status versus in UA matched status. Control U sites were chosen at both the -100 position and +100 position of Ψ sites and analysis were performed the same way as for Ψ sites. mfold were used to draw the secondary structures of 100 nt surrounding the chosen Ψ sites."

6. Enrichment of Ψ at splice junctions is only informative with a comparison against the background distribution of U. Plot both distributions from your dataset and demonstrate that the difference in Ψ distribution is significant.

Our response:

We thank the reviewer for the valuable insights.

We randomly selected 10,000 U sites from the transcriptome and conducted a parallel analysis. To our disappointment, these randomly chosen sites were also found to be located within 100 nt of the nearest splice sites. To delve deeper into this observation, we computed the average distance to splice sites within 800 nt, 400 nt, 200 nt, and 100 nt windows, but still, no substantial differences were observed between the Ψ group and the control U group. We regret the oversight that led to an incorrect conclusion in the previous version of the manuscript. We have removed the erroneous figure and texts that depicted the correlation of Ψ with splicing.

Reviewer #3 (Remarks to the Author):

The manuscript by Wang et al developed ELAP-seq to enrich and map Ψ at single-base resolution. This new Ψ sequencing technology leveraged enzymatic strategy to label and enrich Ψ-modified transcripts. They have identified approximately 10, 000 candidate Ψ sites in HEK293T and HeLa cell lines, a significantly larger number compared to all previous CMC-based and bisulfite-based methods. Based on this approach, the authors found differential Ψ modification upon type-I interferon stimulation in the glioblastoma cell line. Overall, the manuscript provides a new sequencing method and applies it to detect Ψ modification in the human transcriptome. Unfortunately, the major concerns remain non-specificity, unclear false positives, and unclear false negatives. Some weaknesses need to be addressed before this manuscript would be suitable for publication:

Major:

1. The authors reported around ten thousand candidate Ψ sites from HeLa and HEK 293T cell lines. However, more than 9000 sites currently lack validation through existing datasets. Before asserting their claims of high sensitivity, the authors have to verify the specificity of ELAP-seq instead of just providing the ambiguous Ψ lists. The authors should perform perturbation of PUS proteins, such as employing PUS knockdowns or knockouts in these cells, to assign the majority of the newly identified Ψ sites to specific enzymes. This assignment would validate the specificity of ELAP-seq and contribute to functional insights.

Our response:

We appreciate the reviewer's insightful comment.

Before applying ELAP-seq to mRNA, we extensively characterized the method's specificity: 1) The enzymatic labeling reaction showed high specificity towards Ψ over U; 2) the pull-down-qPCR experiment on synthetic RNAs showed high signal-to-noise ratios across a wide-range of input amounts; 3) there was high concordance between ELAP-seq and MALDI results on the enzyme's sequence context preference; 4) known rRNA sites were successfully identified (less effective for clustered sites, see response to question #4). Additionally, when applied to mRNA, ELAP-seq preferentially enriched U over other nucleobases by 64-fold. This enrichment was not observed in input RNA or IVT RNA controls (see below), further supporting the specificity of the method.

In this revision, we applied a simplified cutoff (please see response to question #3 of reviewer #1), which decreased the number of candidate sites down to ~8000. Among the 8255 sites detected in HEK 293T cells, 1488 sites overlap with previously reported sites (Figure S14a) while 6767 sites were newly identified. With a more accurate estimation of modification level (please see response to question #2), we categorized candidate modifications sites into various modification levels (page 21 and Figure S13). We found that the newly identified sites are predominantly lowly modified compared to the ones overlapping well with other methods (Figure S14h). Similarly, sites uniquely identified by other methods (e.g., PRAISE) also showed lower modification levels and read coverage compared to the rest sites in their datasets (Figure

S14e). This suggests that discrepancies across methods primarily arise from sites with inherently low stoichiometry or low expression, rather than from methodological artifacts.

To validate the new sites we identified, first, we performed a negative control experiment using modification-free IVT RNA generated from cDNA of HEK293T mRNA (Figure 4g and S9). These RNA samples represent the mRNA transcriptome of HEK293T cells but lack any modification. ELAP-seq on these samples yielded much less sites, which also had minimum overlap with sites detected from native biological RNAs (Figure 49). This supports that most signals we identified in native RNAs are true modifications, rather than artifacts from the labeling or sequencing process.

In addition, as suggested by the reviewers, we performed a knockdown experiment of **DKC1**, which was reported to affect a large fraction of mRNA modification sites (Zhang et al, Nature Chemical Biology, 2023, 19, pages1185–1195). To ensure technical consistency during pull-down and subsequent steps, we employed internally barcoded RNA adapters and pooled control and knockdown samples during library preparation. ELAP-seq analysis of knockdown and control samples identified **1014 sites as DKC1-responsive** (p-value < 0.05 and enrichment level decrease > 2-fold), including **882 new sites** reported here (Figure 4h, S10 and Supplementary Table 5). While this experiment confirms a substantial number of new sites, we acknowledge the limitation of our method's sensitivity in detecting all subtle stoichiometric changes at lowly modified sites, and therefore not all new sites could be experimentally verified through knockdown.

Nevertheless, by analyzing the raw signals of the new sites in the BID-seq dataset, we found that **55.7% of the new sites showed deletion ratios higher than 0.05** in BID-seq dataset but below the 10% cutoff set by BID-seq; thus, these sites demonstrate positive signals despite "not" identified by BID-seq. There are **additional 26% of new sites showing deletion ratios above 0** in BID-seq data. **Only 17.8% of the new sites have no deletion signals in BID-seq** and these sites exhibited significantly lower reads coverage in the BID-seq dataset compared to the overlapping sites, suggesting that **they were likely missed due to low gene expression or they are intrinsically less confident** (Figure S14i-k).

Finally, to facilitate the interpretation of individual site's reliability, we rationally categorized candidate Ψ sites detected by ELAP-seq into different confidence levels (page 18) based on the RT stop signal strength and repeatability of identification. There are 3947 sites categorized as highest-confidence, 2714 as higher-confidence, and 1595 as lower-confidence (Figure S12). We found that sites well overlapping with other methods were mainly found in the highest- and higher-confidence groups, supporting the validity of this classification (Figure S14b). We encourage caution in interpreting sites from the lower-confidence category and recommend further experimental validation. Additionally, we discuss potential sources of false positives and false negatives in our dataset (page 30-31). These meaningfully decrease the ambiguity of the reported sites and helps readers to better interpret the data and the method in this study.

2. A more comprehensive comparison should be performed between the Ψ dataset sourced from HEK293T and HeLa cells in ELAP-seq and their previously published human mRNA pseudouridine maps (Dai, Q. et al. Nat Biotechnol. 2023). They should analyze the raw deletion signal for ELAP-seq-unique Ψ sites in BID-seq classify the authenticity of these sites and provide a clear explanation for their lack of detection in BID-seq.

Our response: We thank the reviewer for the constructive suggestion. This suggestion was highly helpful to validate the authenticity and address the discrepancy. We have conducted a detailed analysis of ELAP-seq-unique sites in BID-seq, and presented the results in Figure S14i-k and a discussion on page 21-23.

Of the 5410 ELAP-seq-only “higher-confidence” and “highest-confidence” sites (sites not found in neither BID-seq nor PRAISE, stop ratio > 0.3 in two replicates or detected in all three replicates) for HEK 293T, 3,017 sites showed deletion ratio of higher than 0.05 in at least one replicate of BID-seq, thus these sites demonstrate modification signals in BID-seq as well, although they are below the deletion ratio cutoff used by the method (Figure S14i-j). Among the rest sites, 1,429 sites have deletion ratio of between 0.0 and 0.05 in BID-seq with 82% of them having average deletion ratio among three replicates of between 0.02 to 0.05 (Figure S14i-j). There are 964 sites having deletion ratio of 0 in BID-seq data, and these sites exhibited substantially lower reads coverage than sites detected by BID-seq (Figure S14k), suggesting that these sites were missed by BID-seq due to low gene expression. This further validates the authenticity of sites identified by ELAP-seq.

3. In L187, the strong bias for ΨU context might even underestimate the GUΨC context modified by TRUB1 protein which has the highest modification activity, which indicates the severe false negative of ELAP-seq. The authors need to demonstrate they could detect all these TRUB1-dependent sites from previous Ψ datasets.

Our response:

Good point. Our method detected 220 (68%) of the 324 TRUB1-dependent sites reported by PRAISE (see Figure S14f). In comparison, BID-seq detected only 110 (34%) of the same 324 sites. Most of the TRUB1-dependent sites not overlapping with sites detected by our method exhibited signals in at least one replicate of our dataset, but did not meet the cutoff criteria or were present in only one replicate. These sites also exhibited notably lower coverage and modification levels in the PRAISE dataset compared to the rest Ψ sites (see Figure S14g), suggesting that they are intrinsically more challenging to detect due to the lower modification and expression levels. Notably, PRAISE employed a sequencing depth approximately 10 times greater than ours, which likely enabled the detection of these sites that our protocol missed. It is possible that our method could also detect these sites with deeper sequencing.

We discussed that false negatives could originate from the enzyme's sequence bias, thereby our method could miss sites in non-ΨU contexts especially when their expression or modification levels are low (page 29-30). We propose that this limitation could be addressed by higher sequencing depth, and a future endeavor of protein engineering to diminish the sequence preference issue (page 29-30).

4. The authors claim the high sensitivity and low background of ELAP-seq; however, they have yet to provide substantial supporting evidence for this assertion. (1) For Ψ in rRNA, they only identified 70 out of 104 known Ψ sites in rRNA (L250), since Mj1640 the strong preference for 5'-ΨU-3' sequence context in oligo RNA, they should analyze these 34 missed known Ψ sites, and then address whether these omissions were a result of the sequence context instead of being clustered within specific rRNA regions. (2) For Ψ in mRNA, although the author can filter out these highly modified Nm sites through bioinformatic cutoff, they cannot exclude the existence of false-positive Ψ sites derived from Nm modifications that vary with differential modification levels in mRNA, hence, the reliability of ten thousand of Ψ is questionable. The comparison and overlap analysis of Ψ sites identified via ELAP-seq with Nm sites identified through Nm-seq should be performed (Dai, Q., et al Nat Methods, 2017).

Our response:

This is a great comment. We claimed that our method has high sensitivity and low background based on the following observations and reasonings: 1) the enzymatic labeling is highly specific for Ψ (complete labeling on synthetic Ψ-RNA in 5 min and no labeling on U-RNA even with extended incubation) (Figure 1b); 2) the synthetic RNA pull-down experiment exhibited high signal-to-noise ratio (Figure 2b); and 3) compared to methods not involving enrichment, our method enriches modified RNAs and signified RT stop signatures, which in principle facilitates the detection of lowly expressed and modified species with shallower sequencing depth.

We acknowledge that the method is less sensitive for clustered sites, which is evident in the case of clustered sites in rRNA. By scrutinizing the 34 rRNA sites missed by EALP-seq, we confirmed that this was due to the site clustering (Figure S5c). We have discussed this limitation in the main text (page 29)

Regarding the potential Nm sites, we clarify that those sites were filtered based on the presence of a stop signature at the same location in the input sample. If the modification level is too low in the input to be filtered out, it would not be detected in the pull-down sample either, as the signal wouldn't be magnified during pull-down, unless the modification co-occurs with Ψ, which has not been supported by the literature. We performed a comparison with Nm sites reported in the literature (Dai et al, Nature Methods and Li, et al Cell research) and found 7 overlapping sites for HeLa and 1 overlapping site for HEK 293T. These overlapping sites have been removed from the final list.

5. In L267 and Figure 4a, how does the mitochondrial mRNA Ψ result compare to the previous Ψ modification map obtained by BID-seq? The comparison of mitochondrial Ψ sites should be performed for both HEK293T and HeLa cell lines.

Our response: In our analysis, we identified 17 candidate Ψ sites in HeLa mitochondria mRNA and 25 in HEK mitochondrial mRNA. This set of sites encompassed all five mitochondria mRNA sites for HEK 293T (chrM: 6293, 7329, 9905, 11154 and 14868) and three for HeLa (chrM: 6293, 7329, and 11154) collectively identified by BID-seq and PRAISE. Notably, our

data show that 15 mRNA modification sites were shared between the two cell lines, including sites 9905 and 14868, which were previously identified only for HEK 293T cell line (figure below, S15). It's worth noting that most of the mitochondria mRNA sites uniquely detected by ELAP-seq are characterized as lowly expressed or lowly modified, suggesting potential reasons why they were not missed by the other two methods. We have included the mitochondria sites in the supplementary Tables 1-4.

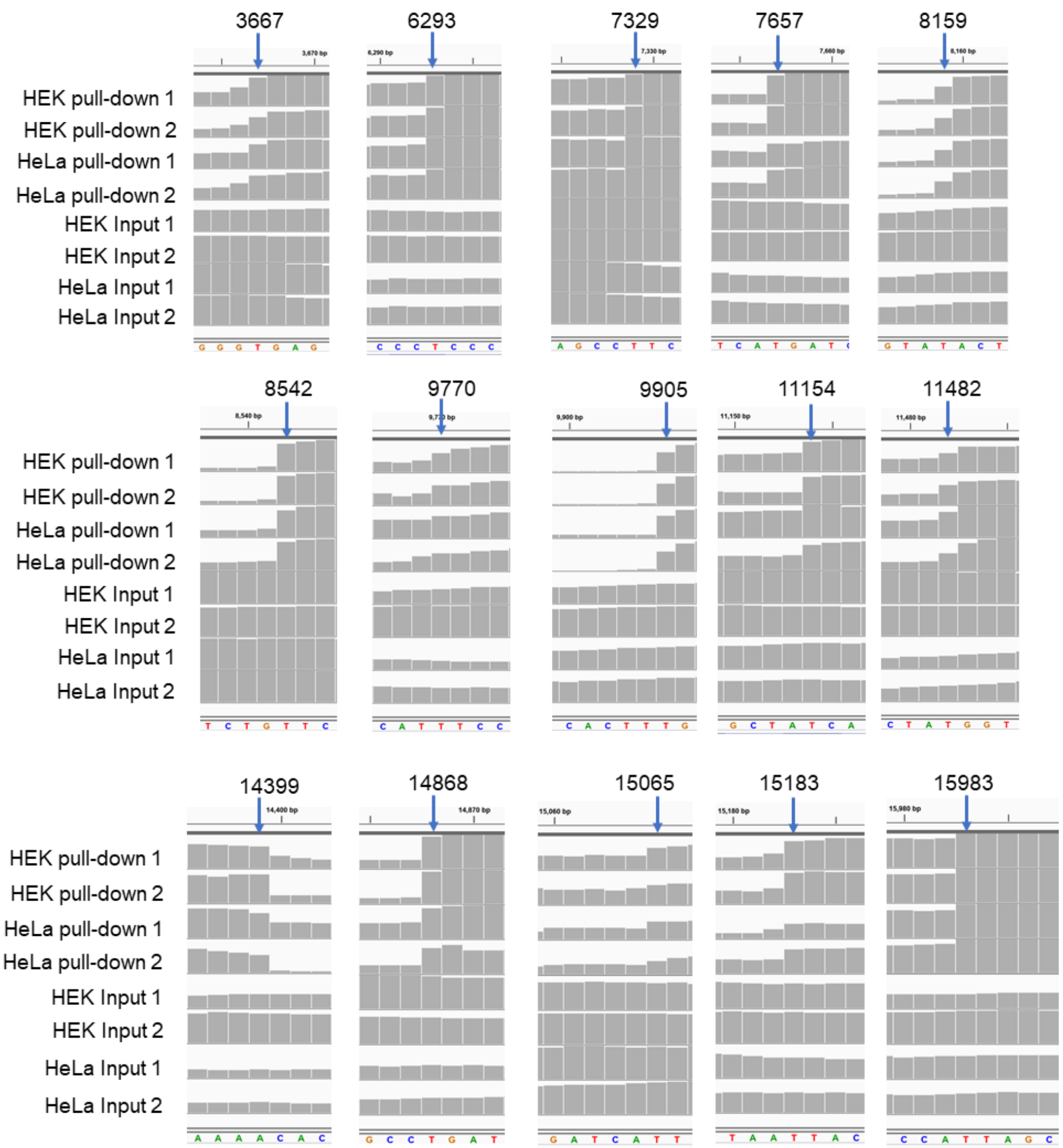

6. In L328, the authors claim “excellent” repeatability for Ψ detection in mRNA. They should provide mRNA Ψ site numbers for three biological replicates and hence the ratio of overlapping sites.

<u>Our response:</u>

We appreciate the reviewer's valuable suggestion and have added a third biological replicate for each cell line as requested.

We first examined enriched peaks called by a routine peak-calling tool MACS2, and found 50-80% peak overlap between every two replicates (Figure S8b), supporting good overlap. However, we didn’t require the existence of a site under an enriched peak to define a candidate

site, as they would be dominated by sites in the ΨU contexts due to sequence preference. Instead, we looked at sites both inside and outside the enriched peaks. This would decrease repeatability due to some non-specifically pulled-down species.

We identified 25945, 25871 and 22759 stopped sites at U in each of the three biological replicates, respectively (Figure S8b). To minimize noise from non-specific pull-down of randomly fragmented RNAs, we required that a candidate be reproducibly detected in at least two biological replicates, following a criterion implemented by CeU-seq (Li et al., Nature Chemical Biology, 2015, 11, pages592–597). The three replicates repeatedly identified 5138 sites in HEK293T cells (Figure S8b).

Moreover, correlation analysis of pull-down RPM levels showed high consistency across replicates, with a Pearson correlation coefficient above 0.98 between two replicates (Figure S8a) and a coefficient of variation between 0 and 0.25 across three replicates (Figure S8c), further supporting the consistency across replicates of ELAP-seq.

7. In L432, the authors need to provide more experimental and bioinformatic analysis for 92 downregulated Ψ sites upon interferon-α treatment, for instance, they can analyze the motif and investigate whether they were modified by PUS3 protein. The authors could also detect the protein level of relevant PUS enzymes between IFN- and IFN+ samples to explain why Ψ was downregulated.

Our response:

Great suggestion. Upon scrutinizing the sequence context of the downregulated sites, we observed a consistent pattern that aligned with the overall sequence preference of the enzyme, which emphasized a preference for U on both sides. This finding suggests that the sequence context might be influenced by the enzyme's inherent preferences, limiting its ability to provide substantial insights into whether these sites are PUS3 targets.

We acknowledged in the main text that the inability to characterize sequence motifs of the identified sites is a limitation of the method (page 30).

To delve further, we performed Western blot analyses to examine the expression levels of relevant PUS enzymes—PUS1, PUS3, TruB1, PUS7, PUS10, and DKC1—during IFN stimulation. However, no discernible changes were observed in the expression levels of any PUS proteins (Figure 6e). This led us to infer that the downregulation may stem from regulatory mechanisms acting on Ψ or Ψ synthases, as seen with other types of modifications, such as m6A and inosine (Zhou et al., Nature, 2015, 526, 591-594 and Hong et al., Biosci Rep, 2015, 35(2):e00182).

Minor:
1. In L55, the statement regarding quantitative mapping methods is incorrect, RBS-seq is not a quantitative approach.

Our response: We have fixed our main text: "Examples include recently developed methods RBS-seq, BID-seq and PRAISE, which rely on bisulfite treatment and allow qualitative or quantitative mapping of Ψ transcriptome-wide"

2. **In L130, data was not shown for the control U-RNA.**
Our response: We have added the data for the control U-RNA in figure 1c.

3. In L146, the authors should provide experimental data to demonstrate the capacity of the **Mj1640 enzyme to label the synthetic 8-mer oligo using propargyl-SeAm.**

Our response: We have presented MALDI data demonstrating Mj1640's capability to label a synthetic 8-mer RNA oligo using propargyl-SeAm (see Figure S1d). The reason we characterized the labeling efficiency on the 8-mer RNA oligo using SAM was to show that methylation remains efficient on a completely single stranded RNA oligo, thereby further demonstrating that the labeling is not dependent on the secondary structure of its native tRNA substrate in archaea. However, the labeling efficiency on the 8-mer RNA oligo using proSeAm was lower than reactions involving SAM or on the synthetic 17-mer oligo (see Figure S1). We reasoned that this reduced efficiency is attributed to a combined effect of the suboptimal length of the 8-mer oligo for Mj1640 to effectively bind and the weakened Mj1640 binding to the sterically bulkier propargyl-SeAm. Nevertheless, we do not anticipate this to pose an issue with biological RNAs, as we typically fragment them into 60-200 nt. In fact, we consistently observe efficient labeling on both cellular RNAs and the 17-mer oligo when using propargyl-SeAM.

4. In L158, the **size distribution of untreated mRNA should be provided.**

Our response: We have included the size distribution of untreated mRNA in Figure S3a. Following enzymatic treatment, a slight shift to lower RNA sizes was observed. Importantly, the degradation was mild as the average RNA size was ~ 1000 nucleotides post treatment and there was a high RNA recovery rate ranging from 60% to 80%.

We thank the reviewers for their thoughtful comments that helped us substantially improve this manuscript and strengthen the practical value of ELAP-seq. In this revision, we have:

- Included additional biochemical characterization data (Figure S4) and control spike-in data (Figure S5), further validating the sensitivity and specificity of the technology.
- Improved reproducibility assessment with additional data analysis, addressing the discrepancies across replicates.
- Refined and benchmarked the data processing pipeline and applied it uniformly across all datasets, making the analysis consistent and the interpretation of false positive rate more reliable.
- Provided further clarification of input requirements, quantification capabilities, and enzyme accessibility considerations for ELAP-seq.
- Refined the discussion of potential applications to avoid overstating in-cell applicability and to include additional applications suggested by new data.
- Responded to other comments raised by the reviewers, including clarifying specific points of the experimental design and data analysis workflow.

Below we address each comment point-by-point.

Reviewer #3 (Remarks to the Author):

During revision, the authors have incorporated a substantial number of bioinformatics analyses. These analyses encompass overlap studies with the PRAISE, BID-seq, and CeU-seq methodologies. Additionally, the authors have performed DKC1 knockdown experiments to identify PUS-dependent sites.The potential false positives and false negatives associated with the technology have been thoroughly discussed in the discussion section. However, two minor concerns remain to be addressed:

1. The authors should provide data from NNUNN spike-in experiments to further validate the specificity of the technology.

Our response: We have conducted ELAP-seq on the NNUNN spike-in, added a new figure (Figure S5) to report the result and incorporated this result in the main text. Importantly, no specific sequence was enriched for the NNUNN spike-in, in clear contrast to the enrichment pattern observed for NNΨNN spike-in. Moreover, compared with the NNΨNN libraries, both the NNUNN and its click control (mock click chemistry) libraries were built with overamplification. Altogether, these results suggest that there is no specific labeling or enrichment of the NNUNN spike-in oligos.

The high specificity of the enzymatic labeling assay was also demonstrated by the new data added in Figure S4 comparing labeling on modified vs unmodified RNAs (in the NNΨ/UNN context) in dot blot assays.

2. In Figure S8b, despite the authors having performed three replicates, the reproducibility of the modification sites is only approximately 20%. A detailed explanation and clarification of this discrepancy are required.

Our response: Thanks to the reviewers for this insightful question. We noticed that some sites with low read coverage may lack any coverage in other replicates simply due to technical variation, leading to low overlap. To remove the effect of technical variation at low-coverage sites and better evaluate reproducibility in identifying modification-specific signals, we focused on sites covered by at least five reads in at least one other replicate (within our filtering criterion of ≥5 stopped reads) and then applied our standard filter criteria for calling RT stop sites. This updated analysis yielded markedly improved reproducibility: approximately 64% of stop sites in each replicate were detected in at least one other replicate, and 32–39% of sites were shared across all three replicates (Figure S10c).

To further address the remaining discrepancies, we examined RT stop sites identified in only one replicate. Because RT stop sites are defined mainly based on the number of stopped reads and the stop ratio, we compared the number of stopped reads between sites unique to a single replicate and those detected in multiple replicates. For example, sites identified only in replicate 1 (Rep1-only sites) exhibited substantially fewer stopped reads than sites also detected in other replicates, and most fell below the detection threshold in the other replicates (Figure S10d). This indicates that the discrepancies mainly arise from sites with near-threshold stopped reads counts. In this study, we define candidate modification sites as those detected in at least two independent replicates, which effectively enriches for sites with robust signals.

These updated results are now shown in Figure S10b-d. We have added this explanation to the main text (page 16, lines 317-322). The associated code was also updated in Github (code availability section).

Reviewer #4 (Remarks to the Author):

In this manuscript, Wang et al. present a new transcriptome-wide pseudouridine (Ψ) site profiling method (ELAP-seq) using the Mj1640 enzyme to modify Ψ sites with a propargyl group and click-chemistry for biotin labeling. The authors achieved enrichment of Ψ by streptavidin pulldown and single nucleotide resolution by harnessing

the RT-stop signature. For the most part, the authors have addressed concerns voiced by Reviewer 1 and Reviewer 3. The most appealing finding in this current manuscript is the detection of many more Ψ sites compared to previous methods, with some sites further validated by orthogonal methods. However, the potential strength of this enzyme-based method appears to be underdeveloped, and the inconsistency of cutoffs and filtering makes it difficult to follow. These concerns, combined with the relative simplicity, quantitativeness, and cost-effectiveness without need for commercially unavailable enzyme of other methods (particularly bisulfite-Ψ adduct methods PRAISE for transcriptome-wide Ψ profiling and BID-Seq for targeted Ψ profiling) severely limit the utility of ELAP-seq to the Ψ research community. As such, the current version of this manuscript is not well suited for publication in this journal, in our opinion. The manuscript would be more useful as either a resource of novel Ψ sites (which would require a lot more validation, particularly for lower confidence sites) or a revelation of novel biology that existing methods cannot capture.

We thank the reviewer for the constructive comments. We would like to address the reviewers' concerns as below:

Utility of ELAP-seq: We have successfully applied the method to multiple cell lines and conditions, reproducing many previously reported Ψ sites and uncovering new ones, many of which have been directly or indirectly validated. These newly detected sites will provide a valuable resource and can guide future targeted investigations using low-throughput assays (e.g., CMC-based primer extension assays). Notably, 10% of ELAP-seq identified sites are of low confidence, thus the majority of sites are reliable.

While enrichment-based approaches are semi-quantitative, they have been highly informative for RNA modification studies, such as the MeRIP-seq method for profiling N6-methyladenosine. Based on the existing data and new data presented in Figure S4 and S5, ELAP-seq shows improved specificity and sensitivity and will outperform previous chemical- or antibody-based enrichment strategies.

Mj1640 is highly stable and can be readily prepared in most molecular biology or biochemistry laboratories in a few days without requiring repeated purification. Alternatively, institutional core facilities and commercial providers (e.g., GenScript, Thermo Fisher Scientific, OriGene, Novus Biologicals) can supply purified protein at modest cost. To support accessibility, we have deposited the Mj1640 expression plasmid to Addgene.

Many widely used biochemical modification-mapping methods similarly make use of purified enzymes (e.g., SAC-m^6A-seq (PMID:35288668) and eTAM-seq for m^6A (PMID 36593412)).

Advantages of ELAP-seq: 1) a high signal-to-noise ratio; 2) low sequencing depth and computational demand compared to non-enrichment-based methods, making it cost- and time-efficient; and 3) requiring a lower RNA input compared to earlier enrichment-based Ψ mapping strategies.

Broad value of this study to the Ψ community: In addition to developing ELAP-seq, this study establishes an efficient and specific enzymatic labeling assay for pseudouridine. The labeling reaction, combined with click chemistry, offers a versatile platform for potential future applications such as: 1) sensitive detection of Ψ for diagnosis purposes (based on new data in Figure S4 and page 7, lines 142-157); 2) enrichment of modified RNAs for interactome studies; and 3) compatibility with future low-input or in-cell adaptations.

Major comments:

1. The authors claim that ELAP-seq could be used in live cells for Ψ profiling, but in the ELAP-Seq protocol they denature fragmented isolated RNA prior to enzymatic labeling. This is a contradiction, because RNA secondary structure is influenced by conditions unique to cells not present in denatured fragmented isolated RNA, including long-range RNA-RNA interactions and RNA-binding proteins. For this reason, it is not obvious to the reader that in situ ELAP-Seq would work. Certainly in vivo ELAP-Seq, as hinted at in Supplementary Fig. S3, would not work.

Our response: We thank the reviewer for this insightful comment. We agree that the current ELAP-seq protocol involves denaturation of fragmented, isolated RNA prior to enzymatic labeling, and that such conditions differ from those in live cells. In our study, we observed that when the enzyme is expressed in mammalian cells, it can label endogenous modifications with approximately 10% efficiency. The apparently lower labeling efficiency in cells compared to that *in vitro* (Figure 1) may be due to factors affecting accessibility of the enzyme to RNA such as protein protection, RNA tertiary structure, and local enzyme versus substrate concentrations.

We agree that the current protocol is not yet optimized for in situ or in vivo mapping, and we have now toned this down in the main text to avoid overclaiming. Future optimizations may enable these applications, such as fusing it with helicase or strand-displacement domains, to transiently unwind structured RNAs for better labeling.

Despite this, our data show that the enzyme is catalytically active in cells and thus has potential for in-cell labeling of pseudouridine. We envision that this system could be developed further as an in-cell site-directed RNA modification tool, where a guide RNA will be used to disrupt local RNA structure, similar to other RNA-guided modification or

editing systems. Since pseudouridine modification is irreversible, no current method exists to specifically remove it at defined sites to study its biological function. We propose that our approach could be used to perturb this modification and investigate its functional role. We highlight this as a potential future application in the discussion section (page 29, lines 586-591).

2. In the Introduction, the authors emphasize that many other methods for profiling Ψ sites require a high level of RNA input and/or deep sequencing. Although the authors showed the detection sensitivity of this assay on synthesized oligos in Figure 2b, the detection limit of this assay on real mRNA samples from cells is unclear (based on the Methods section, ELAP-seq appears to be carried out with 200-1000ng of RNA, but it is unclear if it is total RNA or isolated mRNA). In this aspect, it remains uncertain whether ELAP-seq can be used for rare cell populations that other methods could hardly approach.

Our response: In the Introduction, we referred specifically to CeU-seq, which functions similarly to ELAP-seq as an enrichment-based Ψ mapping method, that requires much higher RNA input (10 µg of mRNA, approximately 50-fold higher) than our method. Similarly, another enrichment-based method using antibody also requires 10 µg of mRNA (Campos et al., PMID: 34376564). For other existing methods, our discussion focused primarily on their need for deep sequencing, rather than their RNA input requirements. We have further clarified this in the Discussion, under the section of Advantages and Limitations of ELAP-seq (page 27, line 532).

As stated in the materials and method section, 200–1000 ng of RNA (mRNA) was used for the initial fragmentation step, with a typical yield of ~80-100%. From this fragmented RNA, we aliquoted ~200 ng for downstream library preparation. Therefore, ELAP-seq currently requires ~250 ng mRNA input and is comparable to PRAISE in input requirements (500 ng mRNA). These details were included in the original version and are now further clarified in the revised Materials and Methods section (page 34, lines 692-693).

Enrichment-based methods generally require higher RNA input than non-enrichment-based approaches, due to loss of materials during binding and washing. ELAP-seq uses mild labeling conditions, which preserves RNA, and the streptavidin-biotin pair, which provides high binding affinity and can withstand stringent washes. These features make the required RNA input lower than previous enrichment-based methods and comparable to that of recent non-enrichment-based methods.

We clarify that the current implementation is not intended for rare-cell applications. It is noteworthy that the use of a low dNTP concentration in our library preparation pipeline

necessitates a starting material of 250 ng of mRNA. We are actively developing a next-generation, low-input version that does not require low dNTP or enrichment, enabling substantially lower input requirements and reduced background.

3. In lines 234-236 the authors decided to use data generated with Superscript III for detecting Ψ sites, while in lines 287-289 the authors decided to combine Superscript III and Superscript IV data to yield better signal. The inconsistency of data analysis makes it difficult to follow. It might be better to fix a filtering and cutoff strategy with rRNA benchmarking and then stick to the same filtering so that the level of false positives can be more easily estimated.

Our response: We appreciate the reviewer's comments about the inconsistency in data analysis. We have now explained the rationale for using Superscript III and IV data. We also applied refined and fixed filtering criteria throughout the analysis. These criteria were first benchmarked using rRNA to ensure robustness before being applied to mRNA data. This new analysis changed the numbers of identified candidate modification sites to 5,366 and 5,741 for HeLa and HEK293T poly(A) RNAs, respectively. We have updated the manuscript throughout accordingly.

We have modified the main text to clarify the usage of Superscript III and IV (page 15-16, lines 291-306):

"While Superscript III with low dNTP produces prominent RT stop signature, we noticed that the coverage at some sites in mRNA is below the reads coverage cutoff although both replicates share strong stop pattern. To achieve higher read coverage, we also used superscript IV with the commercially recommended concentration of dNTP (0.5 mM final) to generate libraries in parallel from the same pull-down product. Interestingly, superscript IV also generated stop signatures at Ψ, although to a lesser extent (Figure S7a), suggesting that the on-bead RT reaction used in our library construction protocol further facilitated the generation of the stop signature (Figure S7a). To rescue additional sites by increasing the reads coverage, we combined data from superscript IV and III for further analysis. We first tested this analysis strategy on rRNA and verified that, with slightly more stringent cutoff in stop ratio (See Materials and Methods section), this strategy did not introduce additional false positives. Additionally, this analysis enabled the identification of the two modification sites in 5.8S rRNA, which were previously missed due to low coverage (Figure S7e).

Applying the same analysis on mRNA identified an additional 1,628 and 2,410 candidate sites from HeLa and HEK293T poly(A) RNA, respectively, yielding a total of 5,366 candidate sites for HeLa and 5,741 for HEK (Figure 4d, Figure S9b). Of these, 2, 531 candidate Ψ sites are shared between HEK293T cells and HeLa cells, accounting for

~46% of all candidate sites in each cell line (Figure 4f). We note that some sites passing the filter in one cell line but found in only one replicate in the other cell line were not included. Therefore, the actual number of shared sites between the two cell lines may be higher than reported here."

We have updated and moved the description of the filter criteria to the Materials and methods section:

On page 39-40, lines 813-823: "A candidate site in rRNA is defined when meeting the following criteria: 1) the stop ratio at the site in the pull-down library is > 0.05 in both replicates; 2) stop ratio in the pull-down library – stop ratio in the input library is > 0.05 in both replicates; 3) the number of stopped reads in the pull-down library is >=5 in both replicates; 4) the average value of stop ratio * stopped reads between the two pull-down replicates is >=1.5 (this imposes a more stringent requirement on the number of stopped reads when the stop ratio in the pull-down sample is lower than 30%); and 5) the stop event occurs at uridine (U). Finally, we further remove sites meeting the following criteria: 1) the stop ratio in the input library is > 0.1 and > 1/3 of the stop ratio in the pull-down library, and 2) number of stopped reads in input is > =3 in any replicate. When using superscript III and IV data together, we follow the same criteria except for requiring that the stop ratio in the pull-down library- stop ratio in the input library is > 0.1."

We have added more details about the library preparation:

On page 36, lines 730-733: "Beads after pull-down were divided to two parts for the downstream reverse transcription step, with three-quarters (corresponding to 150 ng starting RNA) used for SuperScript III RT and one-quarter (corresponding to 50 ng starting RNA) for SuperScript IV RT."

4. In lines 278-280 and a few cases later in the manuscript, as well as in Fig. 4c-e, the authors detected Ψ sites at U, G, A, C with different proportions, while when describing their filter strategy (lines 246-247) they only include sites for which "the stop event occurs at uridine (U)." It is unclear why these G, A, C sites were still included with their pipeline, unless the authors really mean ΨU, ΨG, ΨA, and ΨC.

Our response: We appreciate the opportunity to clarify this point. The figures in Fig. 4c–e show the distribution of reverse transcription (RT) arrest sites at A, U, C, and G, rather than the final calls of pseudouridylation. Only U positions exhibiting RT-stop signatures are subsequently defined as candidate Ψ sites. Our analysis pipeline records RT stops at all four nucleotides (A, U, C, G), but eventually filters for stop events occurring at U to identify potential Ψ modification sites. To be clear, we do not infer pseudouridylation at

G, A, or C; these positions are presented solely to illustrate background RT-arrest frequencies and to assess specificity, as elaborated below.

For mRNA, where modification sites are largely unknown, evaluating stops across non-U nucleotides provides a measure of specificity and potential false positives. This is based on the rationale that, if no specific labeling and enrichment of pseudouridine occurs, RT stop would occur randomly with similar frequencies at A, U, C and G. Instead, we observed strong U-specific enrichment (>95% for SuperScript III and >90% for SuperScript IV) of RT stop signatures in the pull-down samples whereas input samples showed no such enrichment, confirming that detected stops are specific to pseudouridine, rather than resulting from RNA structure or sequencing artifacts. Similar specificity analyses have been used in prior studies (Zhang *et al.*,PMID 26075521 and Cui *et al.*, PMID 33313824).

In contrast, for rRNA, we omitted reporting results at non-U because the well-characterized Ψ modification sites on rRNA can be directly used for specificity validation, and we don't need to use the data at non-U sites for this analysis. For rRNA, only two non-U stops were detected (Supplementary table 5), consistent with the very low false positive rate and supporting the reliability of using non-U stops to assess specificity in mRNA.

In summary, non-U positions are included in the pipeline for specificity assessment in mRNA. Only the U sites are further defined as Ψ modification sites. We have now further clarified this in the main text (page 15, lines 277-290).

5. In the paragraphs to study Ψ sites in response to IFN treatment, the phenotype was not well explained. By LS-MS/MS, there seemed to be a 20-25% reduction of global Ψ, while by ELAP-seq there seemed to be 41-52% fewer sites. The authors should explain this discrepancy. Additionally, since the protein level of PUS enzymes did not change, it is unclear how robust this assay was. The authors should either demonstrate Ψ change with a different method or identify other enzymes/regulators to explain the phenotype.

Our response: We appreciate the reviewer's insightful comments regarding the discrepancy between LC-MS/MS and ELAP-seq results. The discrepancy likely arises from the different nature of the two assays: LC-MS/MS quantifies the total Ψ content at the nucleotide level, whereas ELAP-seq detects site-specific Ψ modifications, which depend on detection thresholds. As shown in the figure below, the sites that become undetectable after IFN treatment exhibit substantially lower read coverage than sites that remain detectable. Thus, a modest reduction in modification level could cause many low-coverage sites to fall below the ELAP-seq detection threshold, resulting in a larger

apparent decrease in the number of detectable sites (~41–52%) than in total Ψ abundance (~20–25%).

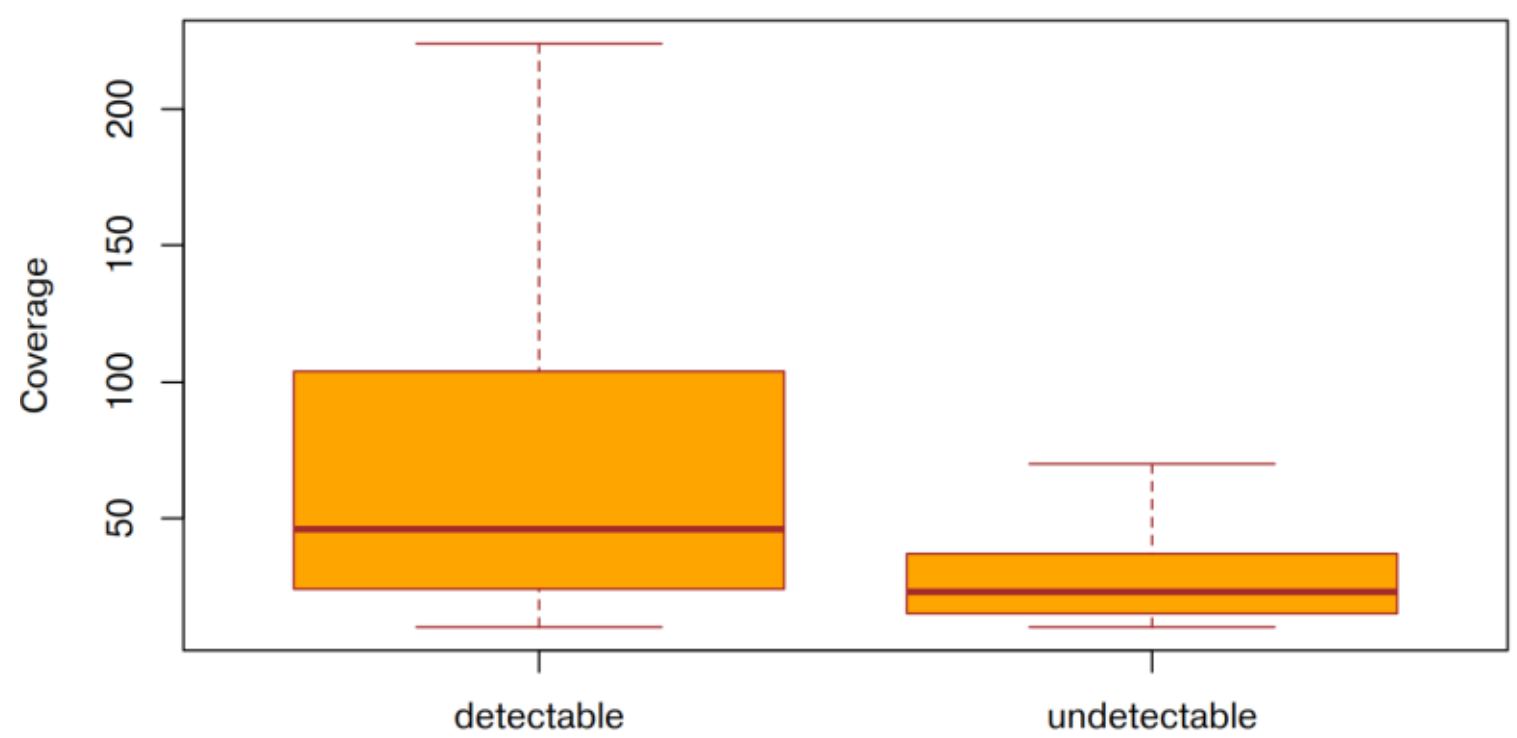

We agree with the reviewer that the study of the IFN treatment is incomplete due to the unresolved mechanism underlying Ψ downregulation phenotype. Fully resolving the mechanism would require further investigation beyond PUS protein abundance, potentially involving post-translational regulation, protein subcellular localization, substrate availability, or unknown RNA-binding regulators. As this mechanistic clarification is beyond the scope of the present study, we have removed the IFN treatment data from this manuscript (previously Figure 6 and S17) and updated the text accordingly. We are actively continuing this line of research as part of a follow-up study.

Importantly, this removal does not affect the robustness of ELAP-seq for differential modification analysis, which has been demonstrated in the DKC1 knockdown experiment. In the DKC1 knockdown experiment, the DKC1-dependent sites determined by PRAISE are also predominantly downregulated in our dataset, supporting the reliable detection of site-specific differential modification using our method.

In addition, this removal does not affect the core findings of the paper, which center on (1) the biochemical characterization of Mj1640, (2) the development of ELAP-seq, and (3) the validation of Ψ detection across transcriptomes.

In the revised version, we have strengthened the manuscript by adding new comparisons with antibody-based detection methods (Figure S4, page 7 lines 142-157) and control spike-in validation data (Figure S5), refining the ELAP-seq analysis pipeline, and expanding discussion of future applications.

6. Pull-down of both enzymatic and control reactions in ELAP-seq means that the methodology will likely be even less quantitative than other semi-quantitative methods for lower RNA inputs. It is hard to imagine that spike-ins mentioned in lines 564-565 would alleviate this concern.

Our response: We thank the reviewer for this comment.

Pull-down of the control group is used solely as quality control during library preparation. Under the same conditions, only modified RNAs in the experimental group are expected to be successfully pulled down and yield detectable libraries, whereas the control group is expected to produce no DNA in the end (Figure S9a), unless being overamplified relative to the experimental group. Therefore, the control reaction does not proceed to sequencing and is not required for data analysis.

In our analysis pipeline, quantification starts with calculating the enrichment level at each site, defined by normalizing the RPM at that site in the pull-down sample by that in the input sample to account for gene expression difference. This enrichment value is further normalized to account for sequence context difference, generating the normalized enrichment. The relative values of normalized enrichment reflect the relative modification levels across different sites. Importantly, our measurements showed moderate to high correlations with other quantitative methods at overlapping sites (Figure 4j and S15e). Based on these correlations, we determined the correlation between the values of normalized enrichment and absolute modification levels, and then accordingly assigned remaining non-overlapping sites to various modification level categories (Figure S15f and the Materials and Methods section).

We envision that including barcoded spike-ins in various sequence contexts with defined modification stoichiometries can improve the current quantification strategy. These spike-ins will undergo the same enzymatic labeling and pull-down treatments as biological RNAs, providing calibration curves that allow direct estimation of modification levels in various sequence contexts. Such spike-in calibration methods have been adopted by many studies (e.g., BACS (PMID:39349603) and PRAISE (PMID:36997645)).

We also note that reads coverage can influence quantification accuracy: mRNA sites in the ΨU context, rRNA sites and mRNA sites in the non-ΨU context readily detectable by CLAP, which generally have high read coverage, correlate better with PRAISE quantification than mRNA sites in non-ΨU context, which generally have low read coverage (Figure 4i and S15e). Therefore, optimizing enzyme sequence preference will also help to address concerns about quantification accuracy. We have now updated the discussion section to further clarify this (page 27, lines 536-544).

Minor comments:

1. In the rebuttal, the authors write that, "Performed knockdown experiment for one of the pseudouridine writers, DKC1, which led to the identification of 1014 sites confidently downregulated due to DKC1 depletion, including 882 sites newly identified by ELAP-seq (Figure 4h and the new supplementary Figure S10)." The authors should analyze how much of this change is due to transcript expression changes leading to lower confidence in calling Ψ sites vs. bona fide Ψ downregulation.

Our response: Our method to measure differential RNA modification uses changes in the enrichment level, defined as the RPM in the pull-down library normalized by that in the input library (please see main text at page 20, lines 380-381 and Material and Method section at page 42, lines 874-876), which already takes into account the change in the transcriptional expression level. Therefore, these are bona fide downregulation, instead of false positives due to a decrease in the gene expression.

2. In line 114, the authors write, "data not shown." They should show the data or remove/restate the sentence.

Our response: Thank the reviewer for pointing this out. Since these negative data were not systematically documented for inclusion in the manuscript, we have now removed this sentence.

3. In line 232-234, the authors do not show Superscript IV data.

Our response: This data has now been shown in figure S7a.

4. The authors should state the precise statistical test used to generate the p-value stated in line 241.

Our response: Please see the updated filtering and cutoff strategy in the Materials and Method section (page 39-40, lines 813-823).

5. In line 466, Fig. 5f should be Fig. 5e.

Our response: This has been fixed.

6. In the Discussion in lines 604-605, the authors speculate that Mj1640 could be used to selectively introduce m1Ψ to mRNA vaccines. It is hard to imagine that such a strategy would be productive, given that such a change would inherently increase immunogenicity and reduce protein translational efficiency.

Our response: We originally proposed such an application based on the hypothesis that the modification may work through decorating a structurally important region in RNA (Nance and Meier, PMID 34075344). However, a recent publication revealed that the modification inhibits TLR7 and TLR8 activation through blocking degradation by RNAse T2 (Bérouti et al., PMID 40580950), making our original hypothesis less likely.

Therefore, we have deleted this from the discussion. We have added other potential applications for our method in the discussion section (page 29-30, lines 583-598).

Reviewer #3 (Remarks to the Author):

Regarding the first point, while the inclusion of the spike-in results in Figure S5 is appreciated, the color scheme requires adjustment as it is currently visually harsh. Additionally, the figure legend needs to be expanded to provide more detailed descriptive information.

Our response: We thank the reviewer for the helpful suggestion. Figure S5 has now been updated with a different color scale, and the legend has been expanded with more details.

Reviewer #3 (Remarks on code availability):

The provided code is user-friendly and the documentation is sufficient. I was able to replicate the installation and execution processes seamlessly.

We thank the reviewer for the positive feedback.

Reviewer #4 (Remarks to the Author):

In their updated manuscript, Wang et al. substantially improve their report on the enzymebased pseudouridine detection method ELAP-Seq. Beyond demonstrating enhanced sensitivity of the method, they have removed incomplete data and unsupported claims. With a few minor changes, their work could be suitablel for publication in Nature Communications.

We thank the reviewer for the positive comments of our revised manuscript.

1. *The authors should add accessibility information on the Addgene-deposited Mj1640 expression plasmid to the Materials and Methods.*

Our response: We have added the deposit ID of the plasmid in the Materials and Methods paper (page 30). The plasmid will be released after the publication of this manuscript.

2. *The authors should remove references to the interferon treatment experiment from the Materials and Methods.*

Our response: The section about interferon treatment has been removed from the Materials and Methods section.

3. I am not convinced by the authors' rebuttal to my previous minor comment 1.

a. For reference, I originally wrote: "In the rebuttal, the authors write that, "Performed knockdown experiment for one of the pseudouridine writers, DKC1, which led to the identification of 1014 sites confidently downregulated due to DKC1 depletion, including 882 sites newly identified by ELAP-seq (Figure 4h and the new supplementary Figure S10)." The authors should analyze how much of this change is due to transcript expression changes leading to lower confidence in calling Ψ sites vs. bona fide Ψ downregulation."

b. The authors respond: "Our method to measure differential RNA modification uses changes in the enrichment level, defined as the RPM in the pull-down library normalized by that in the input library (please see main text at page 20, lines 380-381 and Material and Method section at page 42, lines 874-876), which already takes into account the change in the transcriptional expression level. Therefore, these are bona fide downregulation, instead of false positives due to a decrease in the gene expression."

c. The specific problem I have with this response is what the authors write in the previous paragraph of their rebuttal: "We also note that reads coverage can influence quantification accuracy: mRNA sites in the ΨU context, rRNA sites and mRNA sites in the non-ΨU context readily detectable by CLAP, which generally have high read coverage, correlate better with PRAISE quantification than mRNA sites in non-ΨU context, which generally have low read coverage (Figure 4i and S15e)."

d. There appears to be a clear contradiction—if lower read coverage results in lower quantification accuracy, presumably it also results in lower confidence of called Ψ sites. If this is true, then decreased gene expression can indeed lead to an increase in sites confidently downregulated. As is common in RNA enrichment method benchmarking, the authors should perform additional analysis of their RNA-seq data (Fig. S12a). Specifically, they should assess the degree to which genes harboring 717 (previously 1014—I am not sure what caused the change) Ψ sites confidently downregulated are themselves downregulated in gene expression. I propose that the authors first generate a differential gene expression analysis volcano plot of all RNA-Seq genes with the genes harboring these 717 Ψ sites plotted in a different color. Additionally, they should quantify the distribution in fold-change of the Ψ site-harboring genes relative to the fold-change of all genes. Such analysis will suitably resolve this open question.

Our response: We thank the reviewer for the insightful comments and apologize for not previously fully addressing the concern. We now understand that the key issue is whether reduced gene expression upon DKC1 knockdown could lead to insufficient starting material

for efficient pull-down and thereby cause apparent downregulation rather than true modification level change. Our analyses below indicate that the degree of gene expression change is insufficient to affect the confident detection of downregulated Ψ site, which are now incorporated into Figure S12d–g.

When evaluating modification level changes, we used enrichment level at the modification site, which is defined as the local read coverage per million mapped reads (RPM) in the pull-down library normalized by that in the input library, which accounts for changes in expression levels (a strategy similarly applied in analyzing differential modification in MeRIP-seq data). To evaluate how much of this observed change is due to transcript expression changes leading to lower confidence in calling Ψ sites versus bona fide Ψ downregulation, we performed the analysis described below:

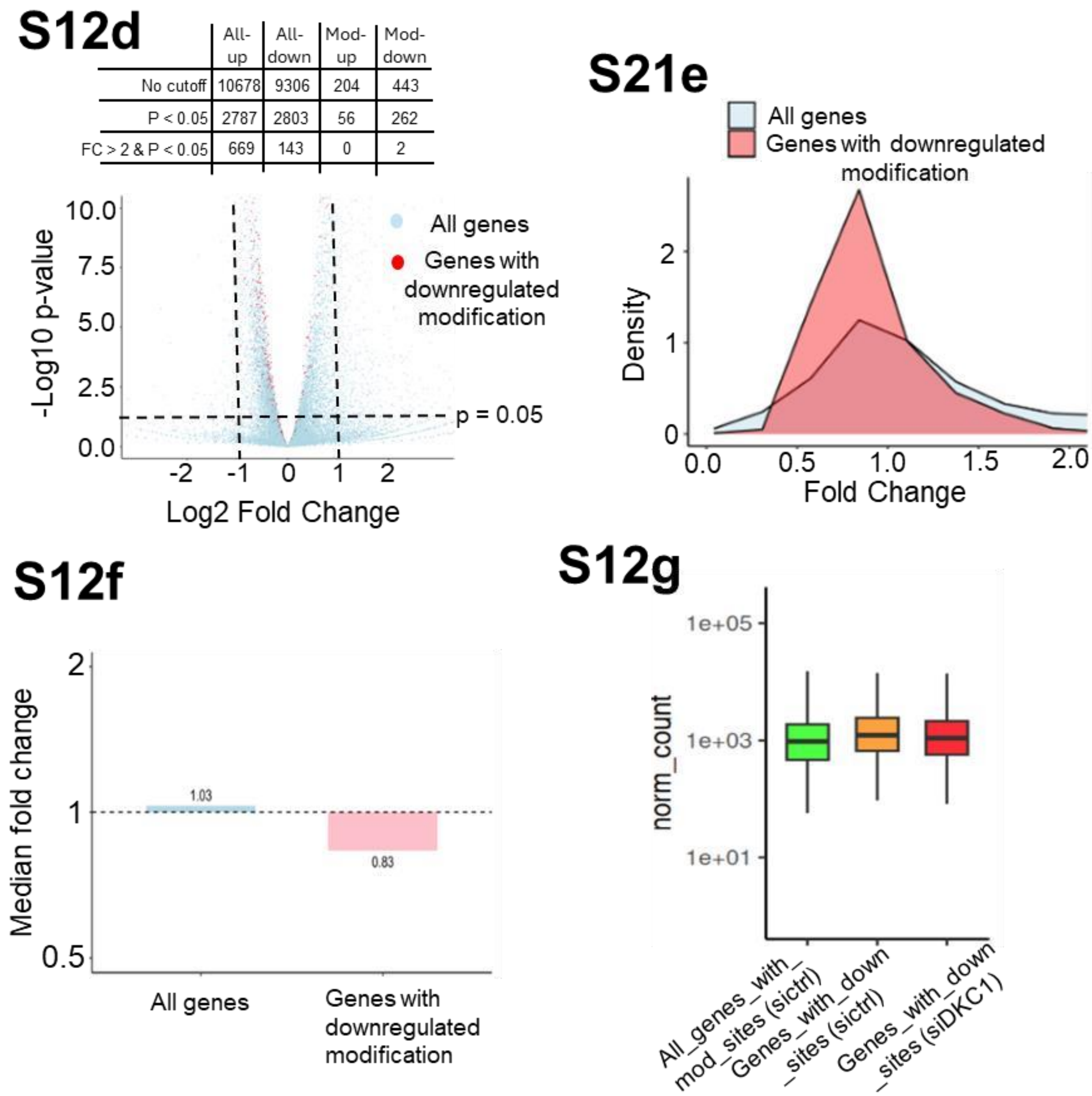

	All-up	All-down	Mod-up	Mod-down
No cutoff	10678	9306	204	443
P < 0.05	2787	2803	56	262
FC > 2 & P < 0.05	669	143	0	2

1. We analyzed differential gene expressions for genes harboring the downregulated modification sites and for all genes detected by RNA-seq using data from the input libraries (prior to pull-down), using DESeq2. We first looked at overall trends of gene expression changes without applying cutoffs for p-value or fold change, and the results are presented in figure S12d-f. The results showed that there is an equal distribution of upregulation and downregulation for all genes (Figure S12d-e), whereas there are more genes being downregulated than being upregulated for genes harboring the downregulated modification sites (Figure S12d-e). The median

values of gene expression fold-changes are 1.03 for all genes and 0.83 for genes harboring the downregulated modification sites (Figure S12f). When applying a p-value cutoff of 0.05, only 262 genes harboring the downregulated modifications are confidently downregulated, and when additionally applying a fold-change cutoff of 2, only 2 genes cross the thresholds (Figure S12d). These overall suggest that although the expression of genes harboring the downregulated modification sites tend to be overall downregulated upon DKC1 knockdown, the extent of that downregulation is mild. Given that modification level changes were assessed by normalizing pull-down-RPM values by input-RPM values to account for gene expression changes, and that stringent cutoffs (p-value < 0.05 and fold change > 2) were applied to define downregulated sites, the mild gene expression changes are unlikely to affect the accuracy of the differential modification analysis.

2. To further evaluate whether the reduced gene expression after DKC1 knockdown could prevent efficient pull-down in the absence of true modification changes, we performed an additional analysis. Genes harboring Ψ sites detected before knockdown represent transcripts with sufficient expression levels to support reliable pull-down and modification detection. Under the assumption that Ψ modification levels remain unchanged, transcripts whose post-knockdown expression levels remain within this range should still be efficiently pulled down. To test this, we compared the distribution of normalized gene-level read counts for genes harboring downregulated Ψ sites after DKC1 knockdown with the distribution for all genes harboring Ψ sites prior to knockdown. We found that these distributions are highly comparable (Fig S12g), suggesting that despite modest expression decreases, the post-knockdown expression levels remain sufficient for efficient pull-down (Fig 12g).

   Based on these results, we updated the main text on page 17: *"The changes in expression of genes harboring these downregulated sites are mild and unlikely to substantially affect the efficiency of pull down of this modification or the reliability of the detected Ψ downregulation events (Figure S12d-g)."*

3. To further increase the confidence in calling downregulated Ψ sites, we applied additional conservative filtering in this revision. Specifically, from the list of downregulated sites, we removed sites with average pull-down RPM values < 1.2 before treatment (corresponding to at least 10× local read coverage at the modification given the current sequencing depth of (6-10M mapped reads)), as such low-coverage sites are more susceptible to technical variation or subtle decrease in

gene expression. This filtering reduced the number of confidently downregulated Ψ sites to 654. The updated results are shown in Fig. 4h.

Additionally, to focus on genes with considerable expression levels and to enable more reliable quantification of enrichment fold change, readers can apply a cutoff of 1 for Input-RPM under both siCtrl and siDKC1 conditions (which corresponds to at least 10x local read coverage at the modification under the current sequencing depth (10-20 M mapped reads) (PMID: 31870409)). Notably, 81% of the downregulated sites are above this cutoff, suggesting high reliability for most sites. This cutoff can be user-defined based on their sequencing depth and research needs.

These changes have been incorporated into the Materials and Methods section: *"For downregulated sites, we further excluded sites with average pull-down RPM values < 1.2 before treatment (corresponding to approximately 10× local read coverage given the current total mapped reads of 6-10M), as their enrichment measurements are more susceptible to technical variation. For more reliable fold-change quantification, readers can apply a cutoff of 1 for Input-RPM under both siCtrl and siDKC1 conditions, which corresponds to at least 10x local read coverage given the current total mapped reads of 10-20 M. Such cutoff can be user-defined based on their sequencing depth and research needs"* (page 44)

4. For clarity, the reduction in the number of downregulated Ψ sites across manuscript revisions reflects the cumulative application of increasingly stringent analysis criteria, rather than changes in the underlying biological conclusions. During the last revision, we applied an improved and more stringent analysis pipeline which has decreased the total number of confident modification sites detected before treatment and consequently reduced the number of sites identified as downregulated upon DKC1 knockdown. The additional conservative filtering applied in the current revision further refined this list.
